# A Structure-Programmable Palette of Compact Cationic Dyes Enables Predictable Organelle Addressing and Multicolor Mitochondrial Imaging

**DOI:** 10.1021/cbmi.6c00056

**Published:** 2026-06-08

**Authors:** Jean C. Neto, Xavier J. Salom-Roig, Eva Falomir, Rosa de Llanos, Juan F. Miravet, Francisco Galindo

**Affiliations:** † Departamento de Química Inorgánica y Orgánica, 16748 Universitat Jaume I de Castellón, Avda Vicente Sos Baynat, s/n, 12071 Castellón de la Plana, Spain; ‡ 27037 Institut des Biomolécules Max Mousseron. Université de Montpellier, CNRS, ENSCM, 1019, route de Mende, 34293 Montpellier, France; § Unidad Predepartamental de Medicina, Universitat Jaume I de Castellón, Avda Vicente Sos Baynat, s/n, 12071 Castellón de la Plana, Spain

**Keywords:** fluorescent probes, Katritzky salts, mitochondrial targeting, multicolor bioimaging, structure−property relationships, live-cell fluorescence microscopy, cancer cell imaging

## Abstract

Cationic pyrylium/pyridinium (Katritzky-type) frameworks have a long history in dye chemistry and have been repeatedly adapted as compact, modular fluorophores for bioimaging. Building on this foundation, we assembled a 20-member library of diversifiable pyrylium/pyridinium salts accessible in one to two steps from readily available precursors, providing a multicolor palette that spans the visible-to-far-red region while maintaining high absorptivity, large Stokes shifts, and bright emission in organic media. In live A549 cancer cells at nanomolar loading, most derivatives rapidly accumulate in mitochondria and show strong colocalization with commercial mitochondrial markers. Importantly, by systematically relating the side-chain structure to calculated lipophilicity, we uncover a practical and predictive localization map: mitochondrial staining is maximized within a narrow cLogP window (ca. 4–6), whereas increased hydrophobicity promotes aqueous aggregation and progressively shifts uptake toward endo/lysosomal compartments. This lipophilicity-governed “address switch” enables organelle destination to be programmed without changing the fluorophore core, providing a practical design guideline for building next-generation chemical imaging tools and functional reporters on pyrylium/pyridinium scaffolds. In addition, the probes can be used for mitochondrial staining in the pathogenic yeast *Candida albicans*, extending their applicability beyond mammalian cells.

## Graphical abstract

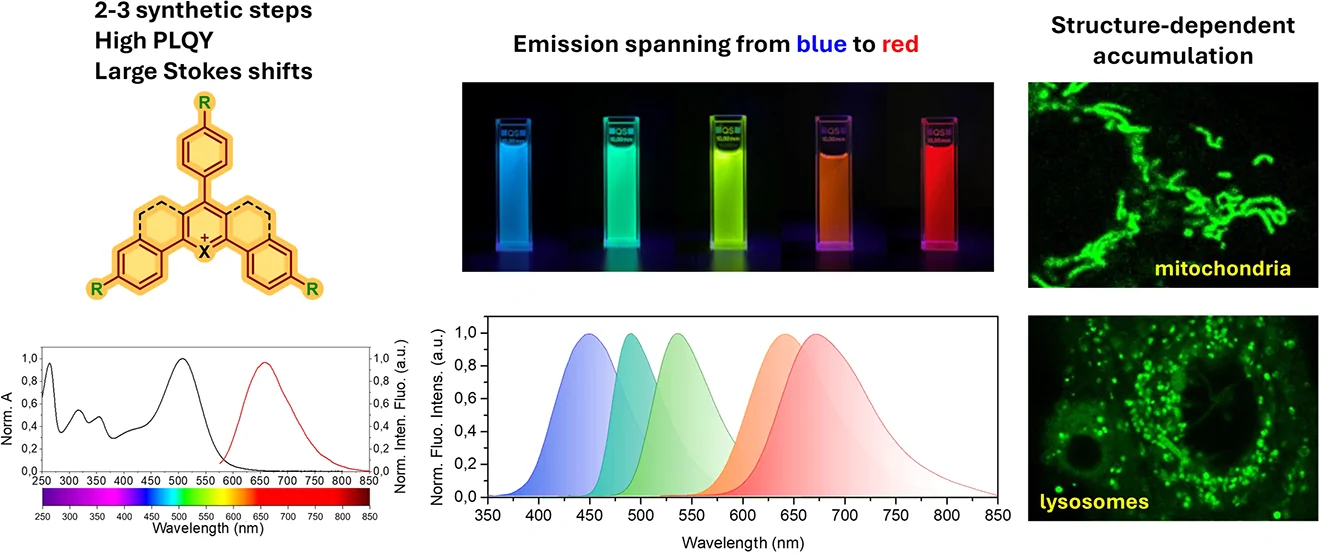

## Introduction

A handful of well-known dye families dominate the bioimaging and phototherapeutic landscape: xanthenes,^1,2^ coumarins,^3^ oxazines,^4^ BODIPYs,^5^ and cyanines^6^ (Figure 1a). The number of publications focused on enhancing the performance of these dye families has increased notably in recent years.^7–13^ This growing interest is driven by the wide range of biomedical applications in which these dyes have found utility. Cutting-edge technologies, such as super-resolution microscopy, advanced phototherapies, and image-guided cancer surgery, would not be possible without the precise tuning of the properties of the photoactive molecules involved.^14–17^ Despite the numerous fluorophores reported to date, several issues remain unresolved. Common drawbacks include poor photostability under high-energy laser irradiation, resulting in photobleaching^18^ and photobluing,^19^ and significant light scattering due to the close proximity of excitation and emission bands, a consequence of small Stokes shifts.^20–22^ Beyond these spectroscopic limitations, an often-overlooked aspect is the synthetic route used to obtain molecules with the desired properties. This issue is far from trivial, as many high-performance dyes used in bioimaging require complex synthesis and purification procedures, which often hinder their practical translation into real-world applications. In this context, synthetic protocols involving only one or two steps are highly sought after.^23–25^

**Figure 1:**
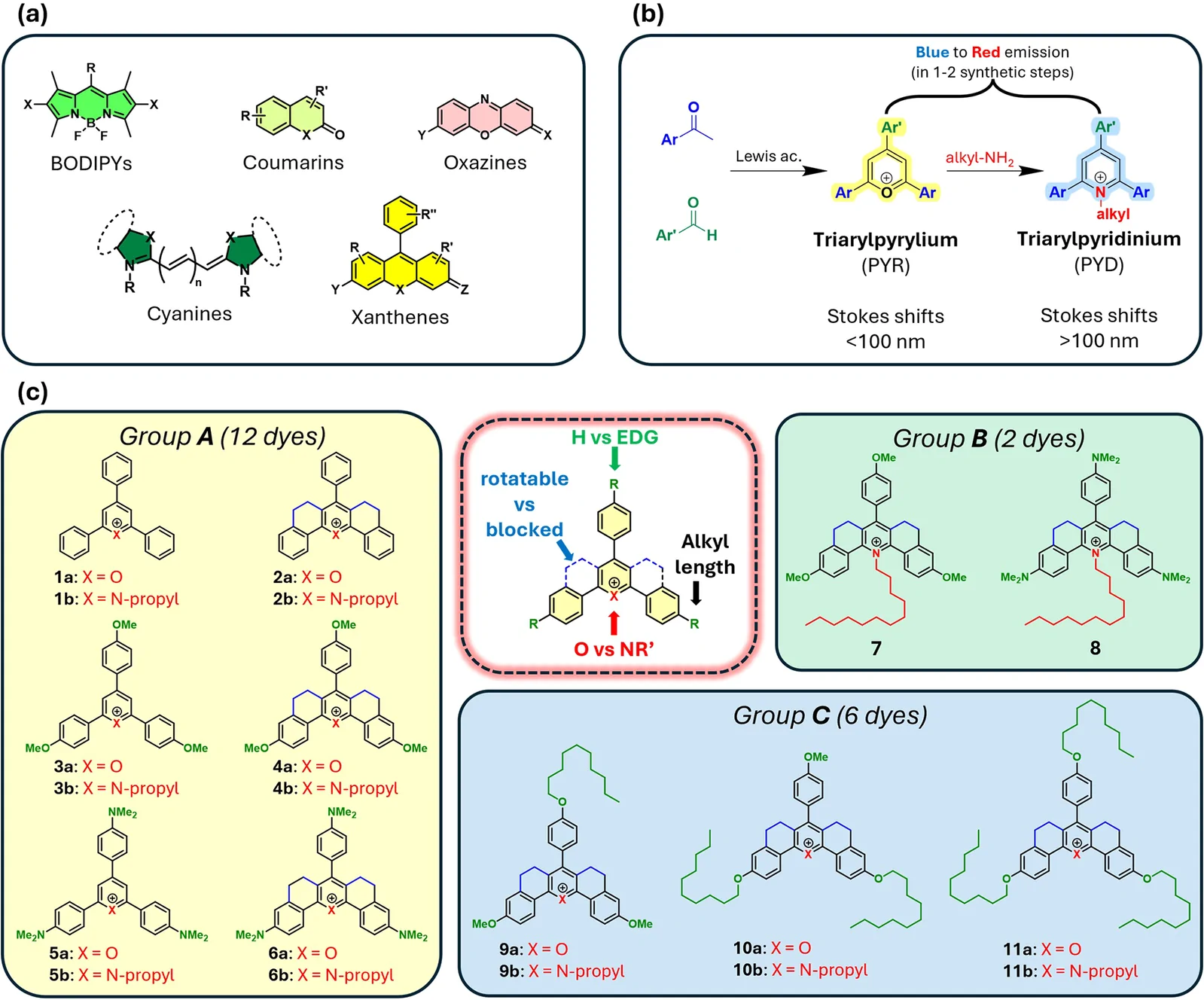
(a) Representative families of classical fluorophores commonly used in bioimaging and phototherapeutic applications; (b) simplified synthetic approach leading to the triarylpyrylium (PYR) and triarylpyridinium (PYD) cations; (c) molecular library of pyrylium and pyridinium cations studied in this work, divided into three groups, **A–C** (BF_4_^–^ is the common anion in all the cases).

Among the dye families with promising potential for bioimaging and phototherapeutic applications are, for instance, cationic aminofluorenes,^26^ thiazolothiazoles,^27^ boronic-acid-derived salicylidenehydrazone complexes (BASHYs),^28^ benzothiadiazoles,^29^ aminonaphthalimides,^30^ imidazo­[1,2-*a*]­pyridinium triflates (PyrAtes),^31^ tetraphenylethylene derivatives (exhibiting aggregation-induced emission, AIE),^32^ single-ring fluorophores bearing various substitution patterns,^33–35^ pyrido­[3,2-*b*]­indolizines,^36^ ECX^37^/BM^38^ dyes, and arylpyrylium cations.^39^ This last group of molecules has been the subject of our research in recent years, including triarylpyrylium and diarylstyrylpyrylium dyes, particularly in various areas such as their application as fluorescent sensors,^40,41^ imaging agents,^42–44^ mitochondrial carriers,^45^ photocatalysts,^46^ solid-state emitters^47,48^ and antimicrobial agents.^49^

As a family of dyes, triarylpyrylium cations are far from new, with descriptions of their optical properties dating back to the middle of 20th century, notably in the pioneering works of A.T. Balaban.^50^ Since then, pyrylium cations bearing various aryl substituents have found applications in a surprisingly wide range of light-related contexts, from laser dyes^51^ and multiphoton absorbing materials,^52^ to chemosensors,^53^ bioimaging probes,^54–57^ and photosensitizers.^58,59^ One of the main advantages of these dyes is their synthetic accessibility, with preparation typically requiring only one or two steps and straightforward purification (Figure 1b). A related family derived from triarylpyrylium salts is the triarylpyridinium compounds, in which the central oxygen atom is replaced by a nitrogen bearing an alkyl substituent. These compounds exhibit greater stability toward hydrolysis and reduction compared to the former, while maintaining similar optical properties. Despite the large number of publications on triarylpyrylium salts over the past 60 years, reports on the applications of their pyridinium counterparts remain relatively scarce. Several research groups have explored di- and triaryl derivatives, reporting imaginative fluorescent systems of interest, especially the groups of A. Samanta and A. Ajayaghosh in the field of biological sensing.^60–65^ Reports of photocatalytic^66^ and electrocatalytic^67^ applications are also frequent in the synthetic literature. However, a systematic study of their absorption and emission properties, and the influence of hydrophobicity, is still lacking, particularly in the bioimaging context. This paper aims to broaden the utility of 2,4,6-triarylpyrylium and, especially, 2,4,6-triarylpyridinium salts (Figure 1c) as versatile tools for biophotonics, given their straightforward synthesis and purification (typically in one or two steps from commercially available products), emission tunability from blue to red, large Stokes shifts, and high photostability. The group of 2,4,6-triarylpyridinium salts is traditionally known as “Katritzky salts”, in recognition of the pioneering contributions of A. Katritzky, who extensively investigated their preparations and synthetic applications.^68^

## Experimental Section

Details on reagents, instrumentation, synthetic procedures, spectroscopic characterization, photophysical measurements, cell treatments, confocal imaging conditions, cLogP calculations, chloroquine-mediated uptake assays, and DLS and TEM analyses are provided in the Supporting Information. Synthetic procedures and full spectroscopic characterization data, including ^1^H/^13^C NMR and HRMS spectra, are provided in Figures S1–S21. Additional photophysical data are summarized in Tables S1 and S2 and Figures S22–S35. Additional CLSM studies can be found in Figure S36. Toxicity assays can be found in Figures S37–S39.

## Results and Discussion

### Synthesis and Optical Properties

The pyrylium salts studied in this work were prepared according to well-established procedures involving the condensation of aromatic aldehydes and ketones, using BF_3_·OEt_2_ as a catalyst.^68^ In a single reaction step, nine triarylpyrylium salts (**1a–6a** and **9a–11a**, hereafter PYR) were obtained from commercially available reagents or from aldehydes/ketones that were readily prepared. A subsequent reaction step, involving the previously synthesized PYR dyes and alkyl amines (N-propyl or N-dodecyl), afforded 11 pyridinium salts (**1b–6b**, **7**, **8**, and **9b–11b**, hereafter PYD). All 20 compounds are shown in Figure 1c. All synthesized compounds were characterized by ^1^H and ^13^C NMR spectroscopy and high-resolution mass spectrometry (HRMS). Full experimental details and spectroscopic data are provided in the Supporting Information. First, the study of group **A** molecules will be addressed, namely, the simplest ones, lacking long alkyl chains. The absorption spectra of PYR cations in group **A** (Figure 2) are characterized by the presence of one or two bands, with molar extinction coefficients ranging from ca. 20,000 to 60,000 M^–1^·cm^–1^, strongly influenced by the substitution pattern on the peripheral rings. The appearance of two bands has been attributed to the presence of two chromophoric systems within the same molecule, aligned along horizontal and vertical axes (*x*- and *y*-chromophores, as described by Haucke and co-workers).^69^ When only a single band is observed, the most plausible explanation is the near-overlap of both bands. Indeed, some absorption bands exhibit a distorted shape, which could be due to this phenomenon. The fluorescence spectra of PYR dyes are characterized by an intense emission band, the position of which also depends on the substituents. The absorption and emission spectra of pyrylium dyes show minimal solvatochromism; however, the fluorescence quantum yield is highly dependent on the polarity of the medium, as shown in Table 1, which compiles all spectroscopic data for group **A** dyes.

**Figure 2:**
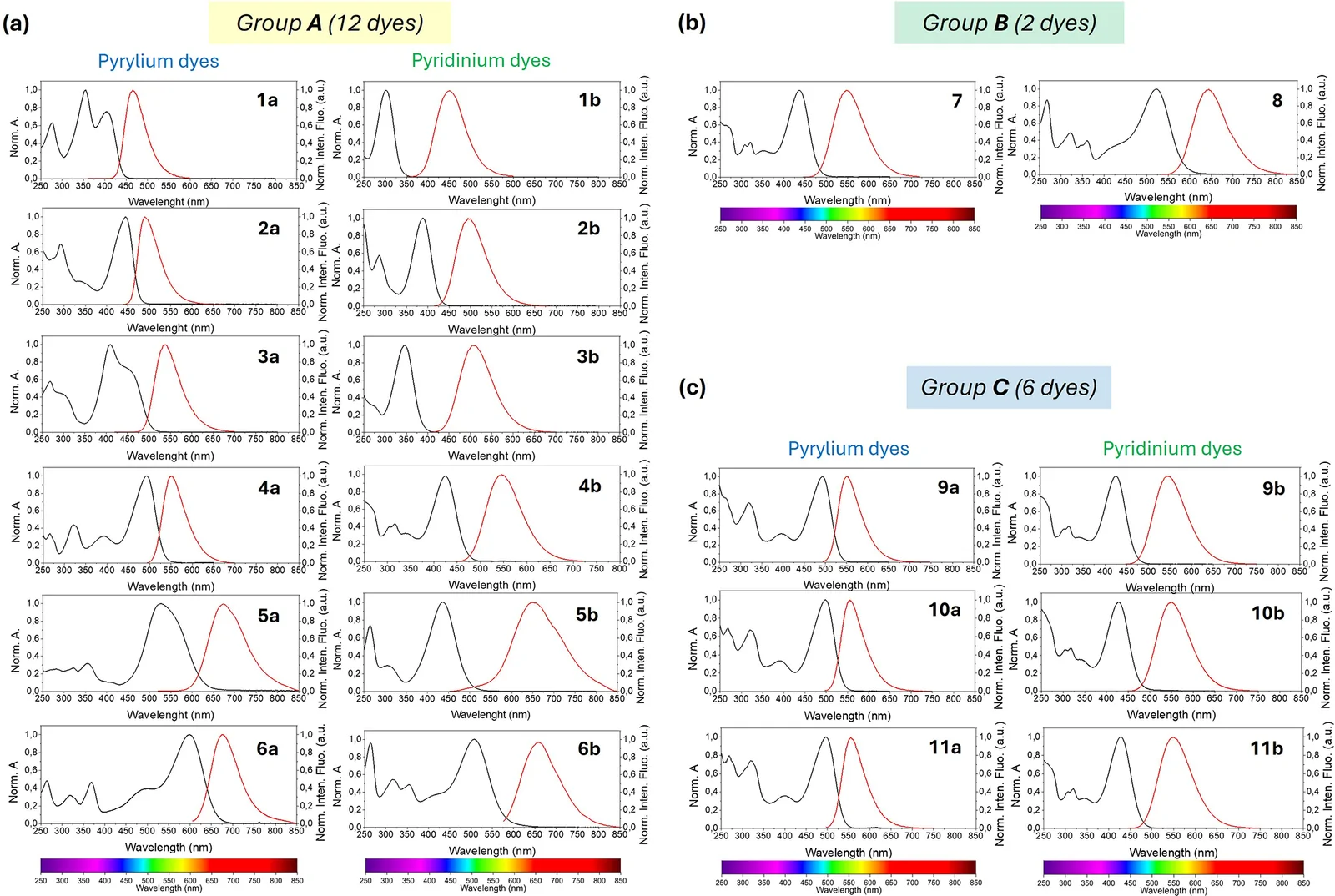
Normalized absorption and emission spectra of triarylpyrylium and triarylpyridinium dyes in acetonitrile. (a) Group **A** includes 12 dyes (**1a**–**6a** and **1b**–**6b**) comprising pyrylium and pyridinium derivatives lacking long alkyl chains; (b) group **B** includes two pyridinium dyes (**7** and **8**) bearing long alkyl chains directly attached to the central nitrogen; (c) group **C** is formed by three pyrylium and three pyridinium dyes (**9a**–**11a** and **9b**–**11b**, respectively) bearing long-chain substituents in the outer rings.

**Table 1:** Photophysical Properties of Compounds of Group **A** (**1a**–**6a** and **1b**–**6b**)­^b^

COMP. (cLogP)^a^	S	λ_abs_ (nm)	log ε	λ_fluo_ (nm)	Stokes shift (cm^–1^)	Stokes shift (nm)	Φ_f_	τ_f_ (ns)	*k*_r_ (10^7^ s^–1^)	*k*_nr_ (10^7^ s^–1^)
**1a** (4.25)	A^(b)^	405	4.54	466	3232	61	0.57	1.90	30	23
	D^(c)^	418	4.26	464	2371	46	0.34	1.96	17	34
	P^(d)^	404	4.45	465	3247	61	0.28	<1.00	NC	NC
**1b** (5.19)	A	303	4.45	450	10,781	147	0.15	<1.00	NC	NC
	D	312	4.49	446	9629	134	0.17	<1.00	NC	NC
	P	300	4.45	447	10,961	147	0.09	<1.00	NC	NC
**2a** (4.85)	A	443	4.39	490	2165	47	0.82	3.75	22	5
	D	459	4.56	494	1543	35	0.86	4.75	18	3
	P	443	4.34	487	2039	44	0.72	4.60	15	6
**2b** (5.88)	A	388	4.31	495	5571	107	0.62	5.47	11	7
	D	395	4.34	499	5276	104	0.54	5.58	96	82
	P	388	4.32	495	5571	107	0.60	5.79	10	7
**3a** (4.14)	A	408	4.56	539	5957	131	0.72	5.41	13	5
	D	425	4.77	529	4626	104	1.00	4.68	21	<1
	P	408	4.56	541	6026	133	0.36	3.89	10	16
**3b** (5.10)	A	345	4.50	507	9261	162	0.58	5.39	11	8
	D	362	4.56	497	7503	135	0.51	4.02	13	12
	P	339	4.51	512	9967	173	0.34	4.59	7	14
**4a** (4.76)	A	493	4.57	551	2135	58	0.83	5.22	16	3
	D	512	4.68	549	1316	37	1.00	4.94	20	<1
	P	486	4.51	548	2328	62	0.63	4.28	15	9
**4b** (5.75)	A	424	4.13	546	5270	122	0.76	6.14	12	4
	D	436	4.49	548	4688	112	0.89	5.61	16	2
	P	419	4.41	543	5450	124	0.70	5.84	12	5
**5a** (4.29)	A	527	4.09	673	4116	146	0.02	ND	NC	NC
	D	536	4.17	645	3152	109	0.63	4.17	15	9
	P	504	4.02	716	5875	212	<0.01	ND	NC	NC
**5b** (5.20)	A	435	4.28	645	7484	210	0.02	<1.00	NC	NC
	D	453	4.39	614	5788	161	0.87	4.63	19	3
	P	431	4.08	536	4542	105	<0.01	<1.00	NC	NC
**6a** (4.87)	A	599	4.66	676	1902	77	0.03	ND	NC	NC
	D	614	4.75	662	1181	48	0.46	3.47	13	16
	P	587	4.33	685	2437	98	0.01	ND	NC	NC
**6b** (5.85)	A	507	4.54	658	4526	151	0.02	<1.00	NC	NC
	D	520	4.57	646	3751	126	0.74	4.40	17	6
	P	497	4.41	663	5038	166	0.01	<1.00	NC	NC

a Notes: (a) cLogP calculated using SwissADME;^70^ (b) acetonitrile; (c) dichloromethane; (d) PBS (10 mM, pH 7.4); ND: not detected; NC: not calculated.

b λ_abs_ and λ_fluo_: absorption and emission maxima, respectively; log ε: logarithm of the molar absorption coefficient; Stokes shift expressed in wavenumbers and nanometers; Φ_f_: fluorescence quantum yield; τ_f_: fluorescence lifetime; *k*_r_ and *k*_nr_: radiative and non-radiative rate constants, respectively

Since this class of molecules has already been extensively studied (see in particular the work of Haucke and co-workers),^69^ no further discussion will be provided here. Furthermore, the primary objective of this paper is to explore the bioanalytical applications of PYR and PYD dyes as organelle markers, with particular emphasis on the structure–property relationships (especially lipophilic character).

When examining the subset of PYR dyes, similar trends are observed, namely, limited solvatochromism and a strong dependence of absorption and emission properties on the substitution pattern. As shown in Figure 2a (left column), the six synthesized PYR molecules cover the entire visible spectrum, with fluorescence ranging from blue to red, simply by tuning the R-substituent and the molecular rigidity. A similarly broad color gamut is obtained with the six synthesized PYD dyes (Figure 2a, right column, and Figure 3c). The colors of the solutions can be seen in Figure 3a under visible- and UV-light illumination.

**Figure 3:**
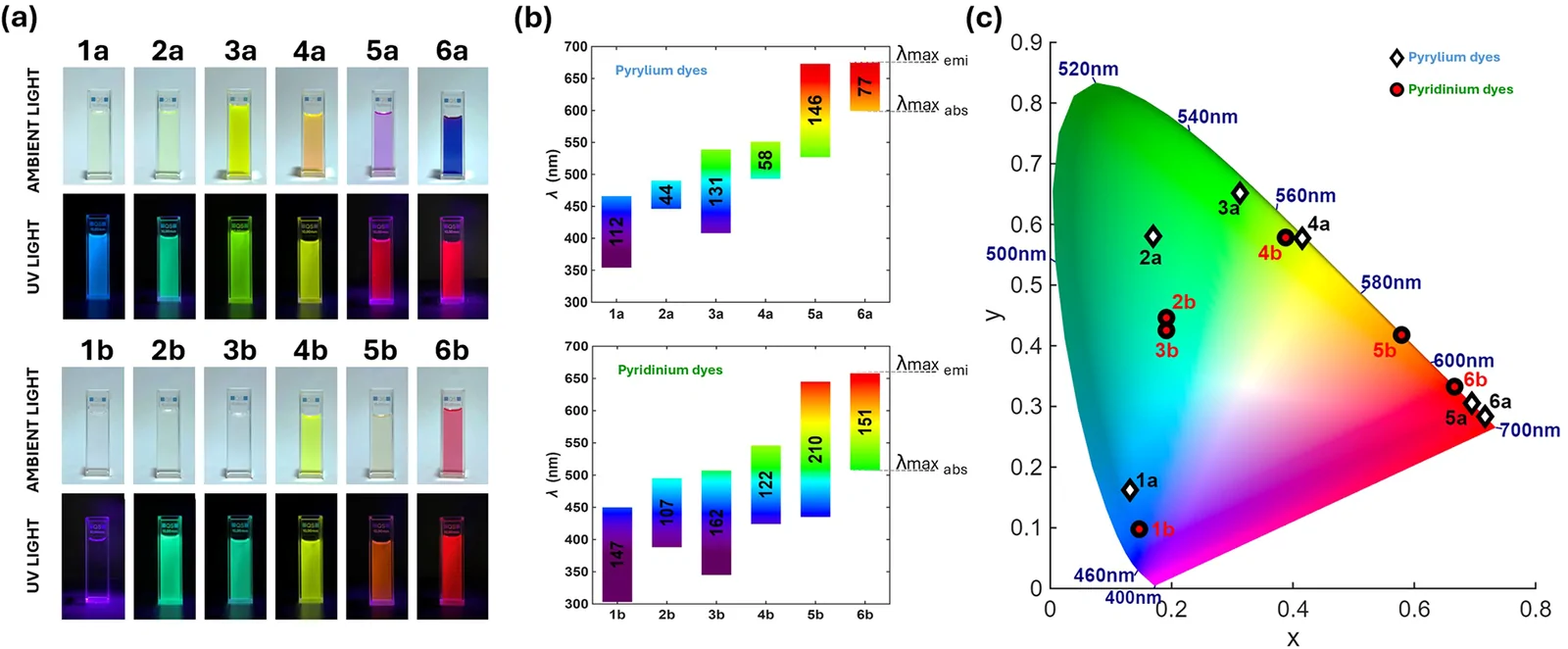
Optical properties of triarylpyrylium (**1a**–**6a**) and triarylpyridinium (**1b**–**6b**) dyes recorded in acetonitrile. (a) Photographs of dye solutions under ambient light (top rows) and UV 395 nm illumination (bottom rows) [Note: compound **1b** shows no visible detectable emission under the experimental conditions, as its absorption maximum (∼300 nm) lies well below the excitation wavelength of the UV lamp (395 nm)]; (b) Stokes shifts of compounds **1a**–**6a** and **1b**–**6b** in acetonitrile; (c) Commission Internationale de l’Éclairage (CIE) 1931 chromaticity diagram displaying the emission coordinates of the dyes **1a**–**6a** and **1b**–**6b** in acetonitrile.

Despite the similarities described thus far, a significant difference can be noted between the two sets of dyes: for PYD dyes, the observed Stokes shifts are markedly larger than those of the PYR group. In general, Stokes shifts for PYD systems exceed 100 nm, with one example (molecule **5b**) surpassing 200 nm (in acetonitrile). For PYR dyes, the Stokes shift values are highly dependent on the molecular rigidity, a factor that appears to have less influence on PYDs. Freely rotatable PYR dyes typically display Stokes shifts of approximately 110–150 nm, whereas rigidified PYR cations exhibit considerably smaller shifts, around 40–80 nm.

This latter phenomenon could be attributed to more pronounced geometric changes between the ground and excited states in the flexible PYRs compared to their more constrained analogues. Twisting in the excited-state geometry has long been proposed as a mechanism for explaining bathochromic shifts in fluorescence bands. However, a transition from a twisted or nonplanar geometry in the ground state to a more symmetric structure in the excited state has also been proposed, most recently by Sissa and co-workers,^71^ to account for the large Stokes shifts observed in certain cyanine dyes.^20^ The current observations for PYR dyes are consistent with this model: the greater the rigidity of the dye, the lower the likelihood of substantial geometric rearrangement upon excitation, and consequently the smaller the Stokes shift. For example, rigid dye **6a** shows a Stokes shift of 77 nm in acetonitrile, whereas freely rotatable dye **5a** exhibits a shift of 146 nm in the same solvent.

To explain the difference in Stokes shifts between the PYR and PYD dyes, the role of the alkyl chain at the nitrogen atom in the pyridinium ring must be considered. The presence of a propyl group hinders the planarity of the x-chromophore, inducing a more distorted ground-state geometry. This distortion contributes to larger Stokes shifts even in rigid systems. For instance, compare the rigid PYR dye **6a** (Stokes shift = 77 nm in acetonitrile) to its PYD counterpart **6b** (Stokes shift = 151 nm in acetonitrile). The difference becomes even more pronounced in freely rotating systems: PYR dye **5a** shows a Stokes shift of 146 nm, while the analogous PYD dye **5b** reaches 210 nm, both in acetonitrile. The propyl substituent prevents complete coplanarization of the chromophore, which likely accounts for the systematically larger Stokes shifts observed in PYD dyes, consistently exceeding 100 nm, while PYR dyes exhibit Stokes shifts ranging from moderate to high values depending primarily on molecular rigidity (see Figure 3b for a visual representation).

Insights into these differences in planarity between PYR and PYD systems can be gained from X-ray crystallographic data available in the literature^72^ or from computational studies of structurally similar PYD dyes,^73^ which emphasize the impact of molecular flexibility and rigidity on geometry and photophysical behavior. Special attention must be paid to the pyridinium systems reported by Samanta and co-workers, used in mitophagy studies, where DFT calculations revealed a notable planarization upon transition from the ground to the excited state.^63^

In conclusion, if large Stokes shifts are desired, then pyridinium dyes are a preferable choice over pyrylium dyes. The alkyl chain at the center of the PYD structure distorts the ground-state planarity, and this effect becomes especially significant in systems with minimal conformational restrictions. While rigid dyes may be favored in specific bioapplications (for instance, due to higher hydrolytic stability), maximizing the Stokes shift is more effectively achieved by allowing rotational freedom in all of the rings.

Regarding photoluminescence quantum yields (PLQY), it is worth noting that both PYR and PYD dyes bearing methoxy substituents exhibit high values in both polar and nonpolar solvents. This is exemplified by compounds **3a,b** and **4a,b**, which show PLQY above 0.5 in rigid systems (sometimes approaching unity). In contrast, their counterparts with dimethylamino groups (**5a,b** and **6a,b**) display high PLQYs only in apolar solvents such as dichloromethane, while in polar solvents like acetonitrile or PBS, the values drop sharply. This behavior is consistent with the strong charge-transfer character typical of donor–acceptor–donor (D–A–D) systems.^7^

Although low PLQY values in aqueous environments may initially seem to limit the biological applicability of these dyes, this is not necessarily the case. Many fluorophores preferentially accumulate in hydrophobic regions such as organelle membranes.^74^ Therefore, low fluorescence in aqueous media does not preclude their use in bioimaging, as will be demonstrated later in this study.

One compound that stands out is **4b**, a PYD cation that combines a high PLQY and large Stokes shifts across solvents of varying polarity. This performance is attributed to the synergistic effects of its pyridinium core, molecular rigidity, and the presence of moderately electron-donating methoxy groups. This observation may have significant implications for the future design of biotools based on the PYD scaffold.

Groups **B** and **C** comprise molecules bearing long alkyl chains either on the central nitrogen atom (group **B**) or on the peripheral aromatic rings (group **C**). Given the goal of this research, to evaluate the suitability of PYR/PYD systems for bioimaging, the hydrophobicity of the dyes was introduced as a key parameter. In many cases, the design of new fluorophores for bioimaging focuses solely on their optical properties, neglecting potential aggregation phenomena, which are common for fluorescent probes in aqueous environments. To address this, structural modifications of the parent dyes **4a**, **4b**, and **6b** were made by incorporating long alkyl chains to modulate hydrophobicity. The calculated cLogP values for all dyes are presented in Table 1 (group **A**) and Tables S1 (group **B**) and S2 (group **C**). Dyes from Group **A** exhibit cLogP values in the range of approximately 4–6. In contrast, dyes with a single dodecyl substituent on nitrogen (compounds **7** and **8**) show increased values of 8–9. PYR and PYD systems with multiple long alkyl chains (group **C**) exhibit progressively higher hydrophobicity, with approximate cLogP values of 7.5–8.5 (one chain), 10.2–11.2 (two chains), and 13–14 (three chains).

Spectroscopic analysis in dichloromethane (DCM), acetonitrile (ACN), and phosphate-buffered saline (PBS) reveals that in organic solvents, whether polar or nonpolar, the absorption and emission characteristics of the modified dyes are similar to those of the parent chromophores with lower hydrophobicity (e.g., **4a**, **4b**, and **6b**). However, in PBS, all dyes containing long alkyl chains exhibit significant shifts in absorption maxima along with a marked decrease in PLQY. These changes are consistent with the aggregation-induced quenching. A notable exception is compound **7**, which retains a high PLQY of 0.93 in PBS. Figure S23 shows a comparison of selected dyes in organic vs aqueous environments.

The reduced emission observed for the most highly hydrophobic dyes should not preclude their application in bioimaging. Although aggregation in aqueous media can initially suppress fluorescence, disaggregation within the intracellular environment, particularly following partitioning into hydrophobic domains such as membranes, can restore emission.^74^ This behavior is further demonstrated in subsequent sections.

### Confocal Laser Scanning Microscopy (CLSM) Studies

After completing the photophysical characterization of these pyrylium and pyridinium salts in solution, we turned to biological systems to determine how these properties translate into imaging performance. Using standardized acquisition settings, we examined the subcellular localization of all cationic probes in the library while accounting for systematic structural variations. This approach enabled a direct structure–property–localization analysis, revealing how substituents, increasing hydrophobicity, and the number and position of side chains govern colocalization and staining patterns.

We began the cellular assays with the structurally simplest subset (group **A**), which allowed us to dissect how key variables govern cell permeability and organelle targeting. These variables include (i) conversion of the central heteroatom from O in PYR cations to N-alkylated nitrogen in PYD derivatives (N-propyl or N-dodecyl), (ii) the electronic nature and hydrophobicity of the *para*-R substituent, and (iii) conformational rigidity.

Group **A** compounds were evaluated in A549 cells (human lung adenocarcinoma), with the exception of **1b**, whose absorption maximum lies below 405 nm and therefore cannot be efficiently excited under our confocal microscope settings. Cells were incubated with each probe (100 nM) for 30 min. Cell culture conditions, incubation parameters, confocal microscope acquisition settings, and data-processing procedures are provided in the Supporting Information. The concentration and incubation time are consistent with those commonly recommended for benchmark mitochondrial probes (MitoTracker dyes), which are widely used due to their high selectivity and robust imaging performance. For colocalization analysis, cells were coincubated with MitoTracker Deep Red (MTDR, 100 nM) when testing compounds **1a,b–4a,b**, and with BioTracker 405 (BT405, 100 nM) for compounds **5a,b–6a,b**.

CLSM images (Figure 4) showed rapid and efficient cellular uptake of group **A** compounds, producing intense fluorescence and high contrast even at low concentrations (100 nM, 30 min). The fluorescence appeared as punctate and/or filamentous structures distributed throughout the cytoplasm, often elongated, branched, or ovoid (Figure 4; magnified regions, probe channel). Such tubular or fragmented, perinuclear/reticular patterns are commonly associated with mitochondrial morphology in CLSM studies.^75^ Beyond this morphological agreement, the cationic and lipophilic nature of these compounds provides a mechanistic rationale for mitochondrial accumulation. Lipophilic cations (LCs) preferentially accumulate in mitochondria driven by the negative inner-membrane potential (Δψm).^76,77^

**Figure 4:**
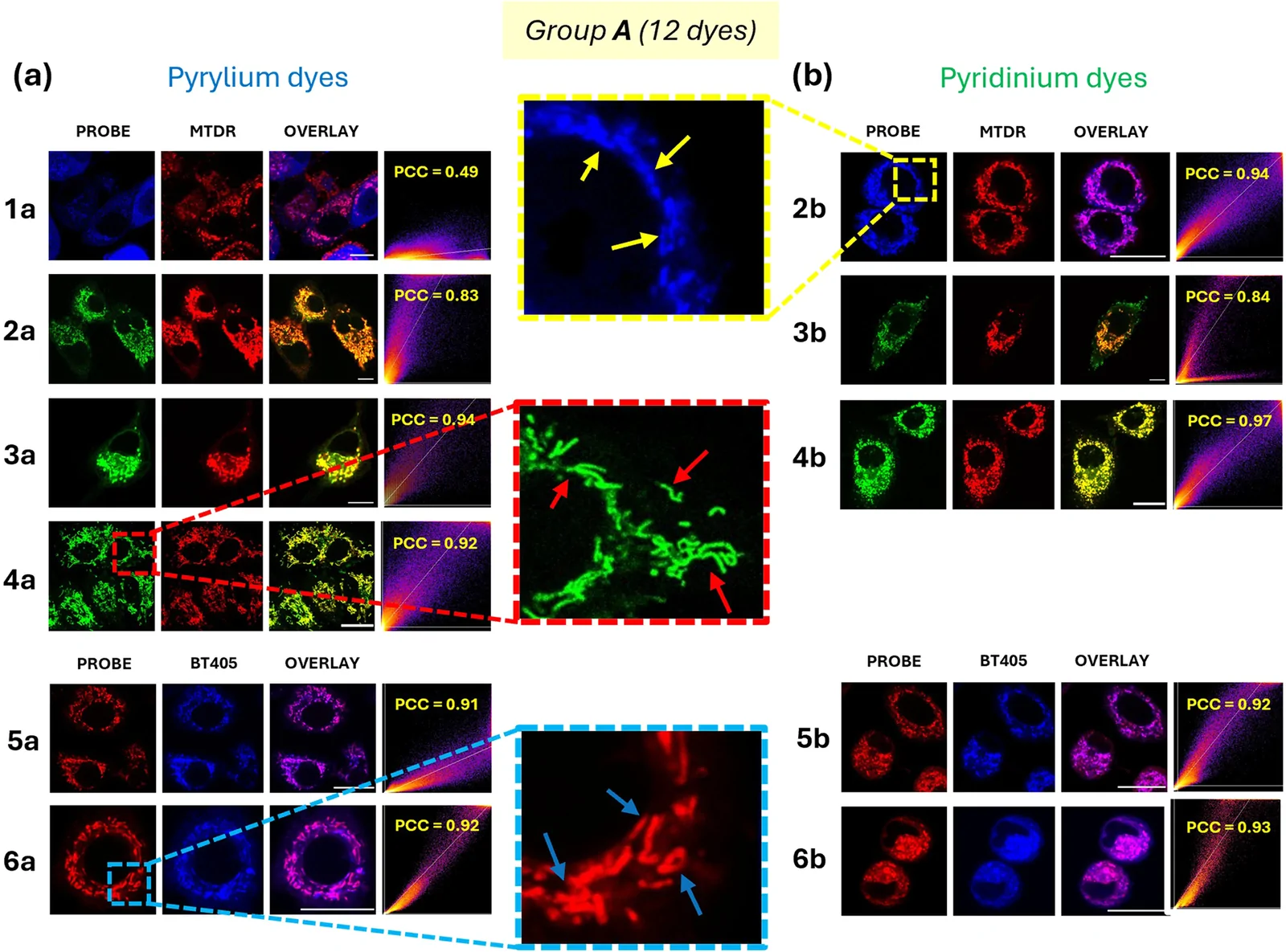
Confocal laser scanning microscopy (CLSM) images of A549 cells incubated with 100 nM (a) pyrylium dyes **1a–6a** or (b) pyridinium dyes **1b–6b**, MitoTracker Deep Red (MTDR, 100 nM), or BioTracker 405 (BT405, 100 nM) for 30 min at 37 °C. Probe excitation and emission settings were individually optimized for each to prevent spectral overlap: BT405 (405 nm) and MTDR (633 nm). Overlay images correspond to the colocalization of the probe fluorescence with the respective commercial mitochondrial tracker. Pearson’s correlation coefficient (PCC, mean ± SD from 10 individual cells) values and corresponding scatter plots are shown for each condition. Magnified views of selected regions of interest are shown for compounds **4a**, **6a**, and **2b**, highlighting intracellular details. Arrows indicate punctate and/or filamentous mitochondrial patterns. Scale bar: 20 μm.

Mitochondrial colocalization with MTDR or BT405 is apparent in the overlay panels of Figure 4, where the probe signal (blue/green/red) overlaps with the mitochondrial tracker signal (red/blue). Regions of spatial coincidence appear as **yellow** pixels (green + red) or **magenta** pixels (blue + red), consistent with concurrent accumulation in the same organelle. To complement this qualitative assessment, we calculated Pearson’s correlation coefficient (PCC) for each condition, which showed a high correlation between probe and tracker channels. PCC is a well-established pixel-wise metric for colocalization that quantifies the linear correlation of fluorescence intensities between two channels. In particular, all group **A** compounds, except **1a**, displayed PCC values between 0.83 and 0.97, consistent with near-complete colocalization. PCC values in this range are generally interpreted as evidence of strong spatial correlation between two fluorescence signals. Taken together with the observed morphology, these results corroborate efficient mitochondrial labeling and support the hypothesis that the probes’ lipophilic, cationic nature drives preferential mitochondrial accumulation, propelled by the negative inner-membrane potential (Δψm), at least for compounds with moderate lipophilicity.

Incorporation of long alkyl chains tunes the lipophilicity–solvation balance, which in turn controls membrane partitioning and dictates the subcellular distribution of fluorescent probes.^78–80^ In group **B** (N-dodecyl substitution), both analogs **7** and **8** showed predominantly mitochondrial labeling, with **8** performing particularly well (PCC = 0.88; Figure 5a). In contrast, **7** gave a lower PCC (0.76) and displayed only partial overlap with the MTDR signal, along with distinct nonoverlapping green-fluorescence regions (Figure 5a, upper inset). This pattern is consistent with partial localization outside mitochondria, potentially in lysosomes or the cytosol.

**Figure 5:**
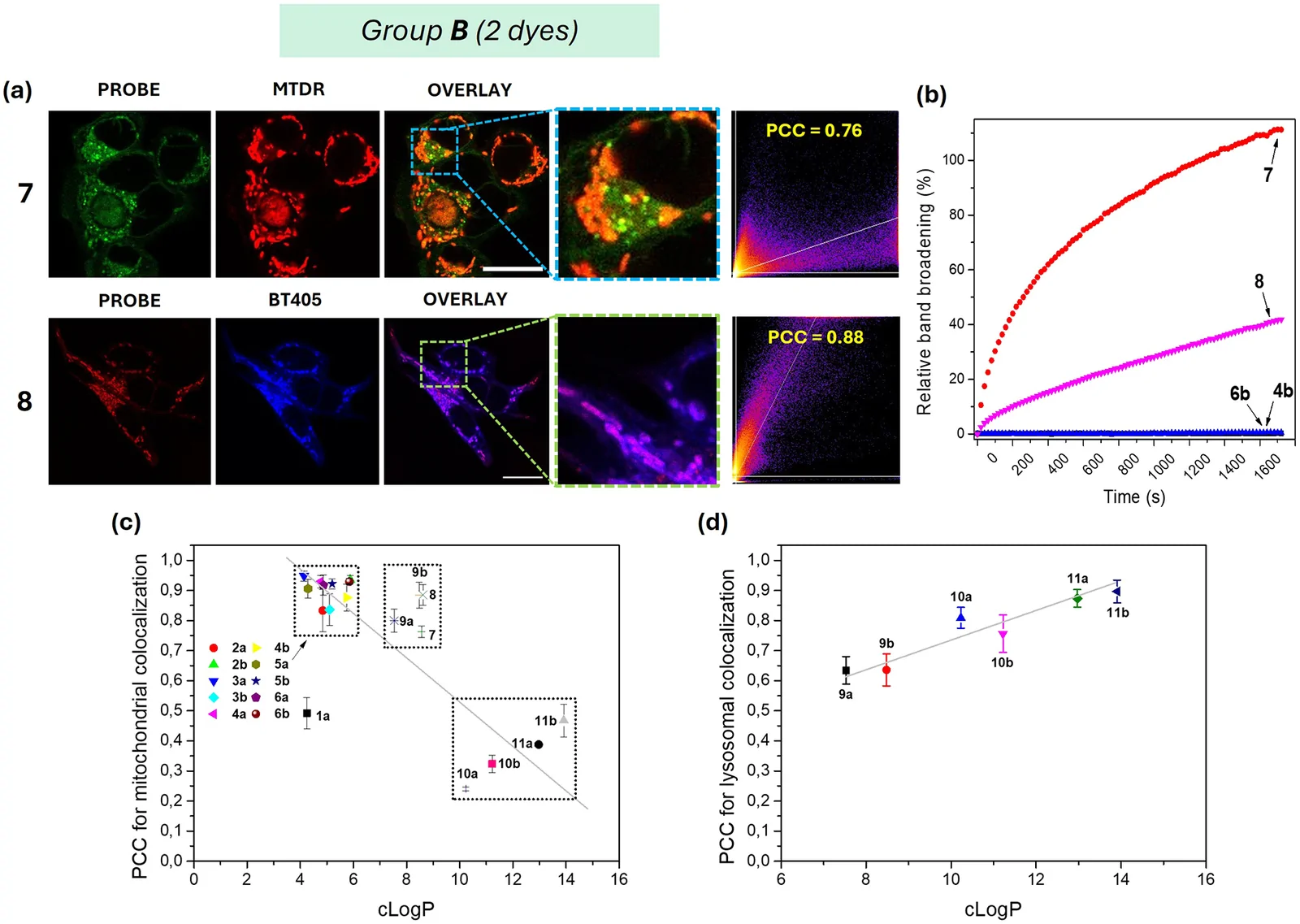
(a) CLSM images of A549 cells incubated with probes **7** or **8** (100 nM) and MitoTracker Deep Red (MTDR) or BioTracker 405 (BT405) for 30 min at 37 °C. Overlay images and magnified regions illustrate mitochondrial colocalization, quantified by Pearson’s correlation coefficients (PCC, mean ± SD from 10 individual cells). Scale bar: 20 μm. (b) Time-dependent aggregation behavior of compounds **4b**, **6b**, **7**, and **8** in PBS. (c) Correlation between mitochondrial colocalization (PCC MTDR or BT405) and calculated lipophilicity (cLogP) for the probe series. (d) Correlation between lysosomal colocalization (PCC) and cLogP for selected compounds.

A working hypothesis to account for the distinct behaviors of groups **A** and **B** is that the N-dodecyl chain promotes aggregation via enhanced self-association in aqueous media. Such aggregation could generate partially aggregated species that, rather than being driven exclusively to mitochondria by Δψm, are internalized by endocytosis and preferentially retained in lysosomal compartments.^81^ This behavior could redirect a fraction of the probe signal away from mitochondria. To test this hypothesis, we examined aggregation in phosphate-buffered saline (PBS) using compounds **4b** and **6b** and their N-dodecyl analogs **7** and **8**, monitoring time-dependent band broadening relative to the isosbestic point (Figure 5b). The N-dodecyl derivatives (**7** and **8**) showed a pronounced increase in bandwidth, most notably **7**, which exhibited an approximately 3-fold larger increase than **8**, when compared with the corresponding N-propyl analogs (**4b** and **6b**). These results indicate faster and more extensive aggregate formation in PBS for the N-dodecyl series. The absorption spectra used for this analysis are provided in the Supporting Information. Collectively, the data support the notion that side-chain architecture (length and substitution site) governs not only aqueous dispersibility but also intracellular compartmentalization.

This hypothesis is further supported by the behavior of group **C**. The strongest mitochondrial colocalization was observed for compounds bearing a single side chain with PCC values of 0.79 for **9a** and 0.88 for **9b** (Figure 6a). By contrast, increasing the number of side chains to two (**10a,b**) or three (**11a,b**) led to a marked decrease in mitochondrial PCC, with the correlation dropping progressively as additional chains were introduced. This trend suggests that a higher number of side chains promotes nanoaggregate formation in aqueous media and, consequently, endocytic uptake, thereby favoring accumulation in endolysosomal compartments. We evaluated this lysosomal redistribution hypothesis using a lysosomal marker (LysoTracker Deep Red, LTDR); the observed colocalization (Figure 6b) is consistent with this interpretation.

**Figure 6:**
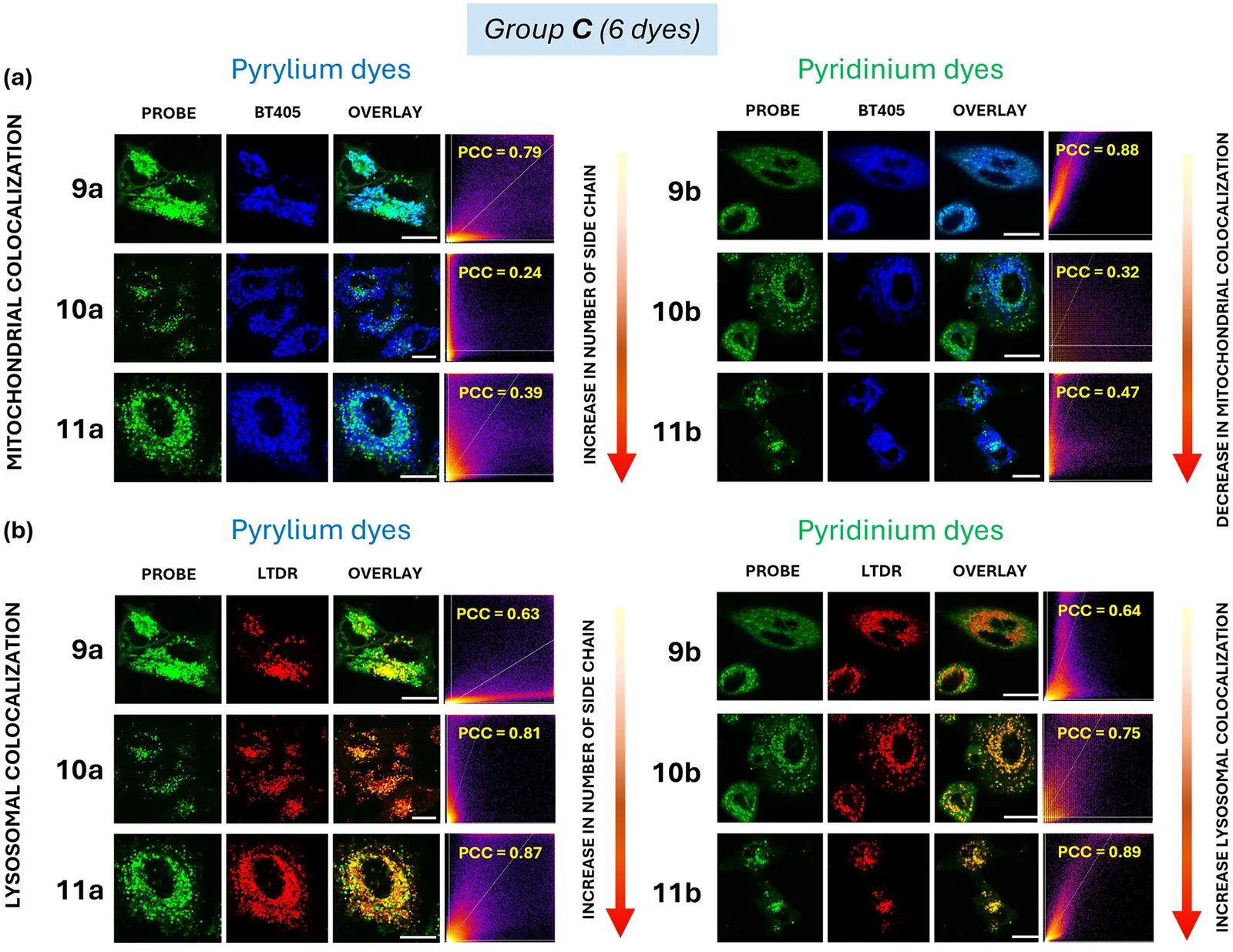
Effect of side-chain number on mitochondrial and lysosomal localization of group **C** dyes. (a) CLSM images of A549 cells incubated with pyrylium (**9a–11a**, left panels) or pyridinium (**9b–11b**, right panels) dyes and BioTracker 405 (BT405) at 100 nM for 30 min at 37 °C. (b) CLSM images of the same dyes coincubated with LysoTracker Deep Red (LTDR, 50 nM) under identical conditions. Scale bar: 20 μm.

The extended series of compounds from groups **A**, **B**, and **C** suggests a hydrophobicity-dependent relationship in subcellular targeting, in good agreement with the aggregation scenario discussed above. For mitochondrial colocalization (Figure 5c), almost all derivatives of group **A**, **2–6a** and **2–6b**, fall within a cLogP window of ca. 4–6 and display PCC values close to 1. At intermediate lipophilicity (cLogP ca. 7–9), group **B** (**7** and **8**) and **9a,b** (group **C**, bearing only one long alkyl chain) fall in a transition region, displaying still-high mitochondrial colocalization (PCC ca. 0.7–0.9) compared with the marked decrease observed for the most hydrophobic derivatives. Moreover, the further increase in lipophilicity arising from the introduction of longer and/or multiple alkyl side chains (**10–11a,b**; cLogP > 10–14) is associated with a systematic decrease in the mitochondrial PCC (ca. 0.25–0.45), whereas the lysosomal PCC (Figure 5d) increases almost linearly with cLogP, reaching values ca. 0.9 for the most hydrophobic derivatives. Therefore, the cLogP windows identified here should be regarded as operational values for this pyrylium/pyridinium series rather than as universal thresholds for all cationic fluorophores.

To further examine whether the highly lipophilic derivatives form supramolecular species under aqueous conditions, compounds **9a,b**–**11a,b** were analyzed by TEM and DLS. TEM imaging of samples prepared from 50 μM solutions of the probes revealed large assemblies with rounded, irregular, wrinkled, and partially collapsed morphologies (Figure 7a). Because TEM samples were dried prior to imaging, these structures should be regarded as dried-state representations of soft colloidal aggregates rather than direct images of fully solvated species in PBS. Nevertheless, the observed morphologies support the formation of supramolecular assemblies rather than isolated molecular probes.

**Figure 7:**
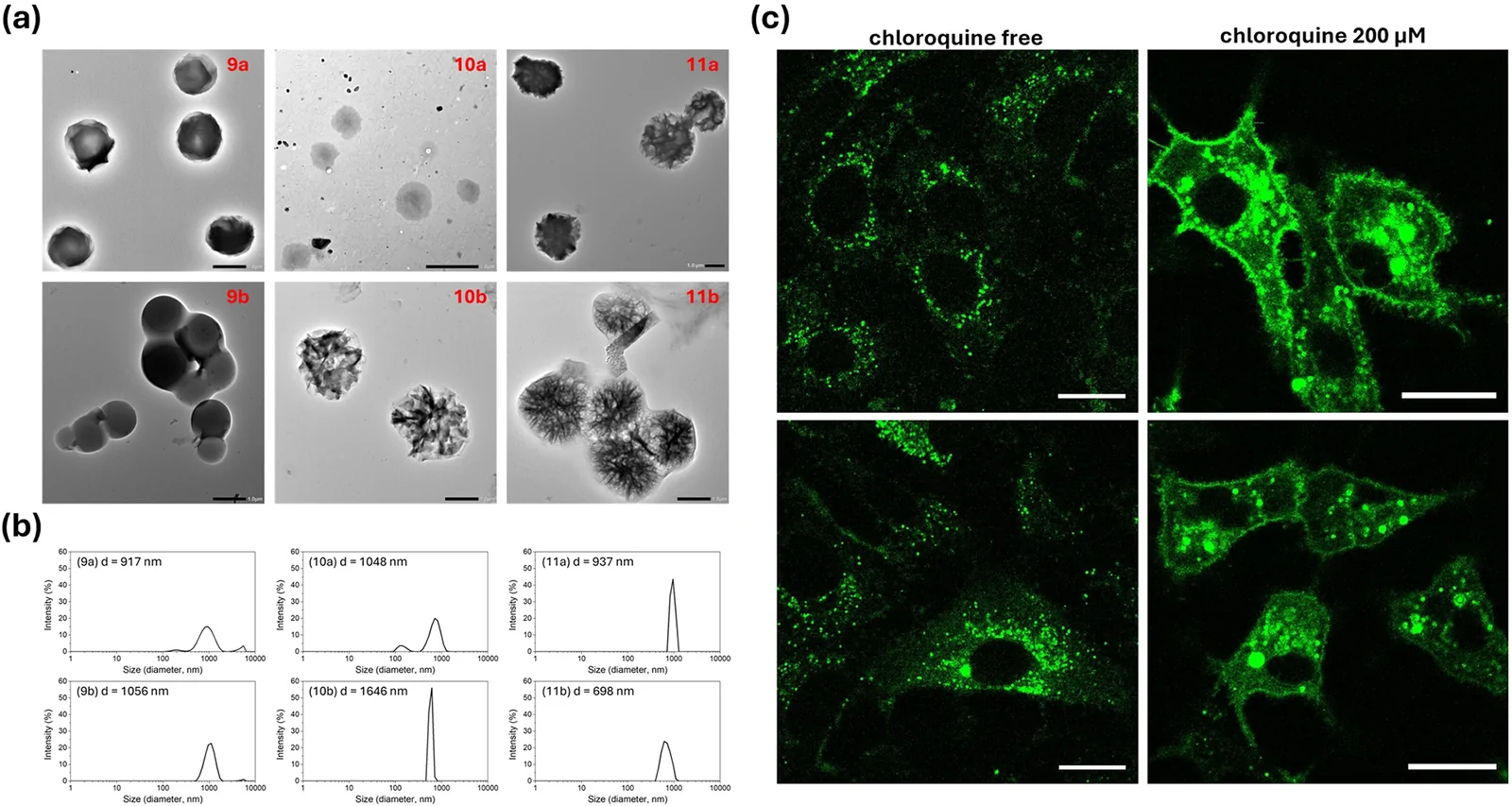
(a) TEM images of aggregates formed by compounds **9a,b–11a,b** in PBS 10 mM, pH 7.4; scale bar: 1 μm. (b) DLS intensity size distributions of compounds **9a,b–11a,b**, with average hydrodynamic diameters. (c) CLSM images of A549 cells incubated with compound **11b** in the absence or presence of chloroquine 200 μM. Scale bar: 20 μm.

Along the **9–11** series, the number of long aliphatic chains increases, while the cationic aromatic core is retained. This amphiphile-like architecture is expected to enhance self-association in aqueous media. In agreement with this view, **9a** and **9b** mainly produced rounded and relatively compact assemblies, whereas **10a,b** and **11a,b** gave more heterogeneous structures with rough surfaces, irregular contours, and wrinkled or collapsed internal textures. These differences are consistent with the increasing hydrophobic contribution imposed by the additional alkyl chains.

DLS measurements performed in 10 mM PBS at pH 7.4 and 50 μM probe concentration further confirmed the formation of colloidal species, with average hydrodynamic diameters in the ca. 700–1600 nm range (Figure 7b). In contrast to less lipophilic analogues such as **4a** and **4b**, which lack long aliphatic chains, the alkylated derivatives **9a,b**–**11a,b** are therefore not present exclusively as monomeric species under these conditions. This supramolecular behavior provides a plausible explanation for the progressive loss of mitochondrial colocalization and the enhanced endo/lysosomal retention observed for the most hydrophobic derivatives.

To probe whether the intracellular distribution of these aggregates is connected to endolysosomal trafficking, compound **11b** was selected for a chloroquine pretreatment experiment. In untreated A549 cells, **11b** displayed a punctate intracellular fluorescence pattern (Figure 7c, left), consistent with vesicle-like structures and with the strong LysoTracker colocalization observed above. After pretreatment with chloroquine (200 μM), the fluorescence pattern was markedly altered, showing increased peripheral/cytoplasmic signal and larger vacuolar-like structures (Figure 7c, right). Chloroquine is known to perturb lysosomal function and endolysosomal organization.^82–85^

Accordingly, these results should not be interpreted as direct proof of a specific internalization pathway but rather as evidence that the uptake and/or intracellular routing of **11b** is sensitive to endolysosomal perturbation. Taken together, the TEM, DLS, LysoTracker colocalization, and chloroquine data support a model in which increasing side-chain hydrophobicity promotes aqueous self-assembly, thereby favoring endocytic/endolysosomal handling over classical mitochondrial accumulation of monomeric lipophilic cations.

Given the structural diversity and subcellular targeting potential of these probes, it is essential to validate colocalization not only with global metrics (e.g., Pearson’s correlation coefficient, PCC) but also with local analyses. Fluorescence intensity line profiles (Figure S36a) provide a direct readout of spatial coincidence, which is particularly useful in complex cellular architectures and for detecting differential distribution among organelles. For compounds **2b**, **4b**, and **6b**, the fluorescence line profiles (Figure S36a) show clear overlap between probe and mitochondrial marker signals, with coincident peaks along the sampled lines, indicating robust spatial colocalization. Complementarily, lambda scanning (λ-scan) (Figure S36b) confirms, with spectral resolution, that the probes’ cellular emission remains compatible with the detection channels used. This approach is especially valuable when working with novel fluorophores or environment-sensitive probes, as it mitigates misinterpretation arising from emission shifts caused by changes in polarity, pH, or membrane interactions.

To extend the scope of our study, we further investigated the probes in nonmammalian cells. Mitochondrial-targeted fluorescent probes are widely used to support fluorescence-based bioanalysis (e.g., microscopy and flow cytometry), but most platforms have been optimized in mammalian systems,^86,87^ whereas mitochondrial imaging in fungi has received comparatively less attention. In pathogenic yeasts such as *Candida albicans*, this translation is not always straightforward because probe delivery and retention can be affected by a thick fungal cell wall (containing chitin, β-glucans, and mannoproteins)^88^ and by drug-efflux transporters^89^ that can expel xenobiotics, including fluorochromes. Figure 8 extends the applicability of the PYR and PYD dyes by evaluating mitochondrial colocalization in *C. albicans* at low concentrations (100 nM, at 30 min) and against standard commercial trackers.

**Figure 8:**
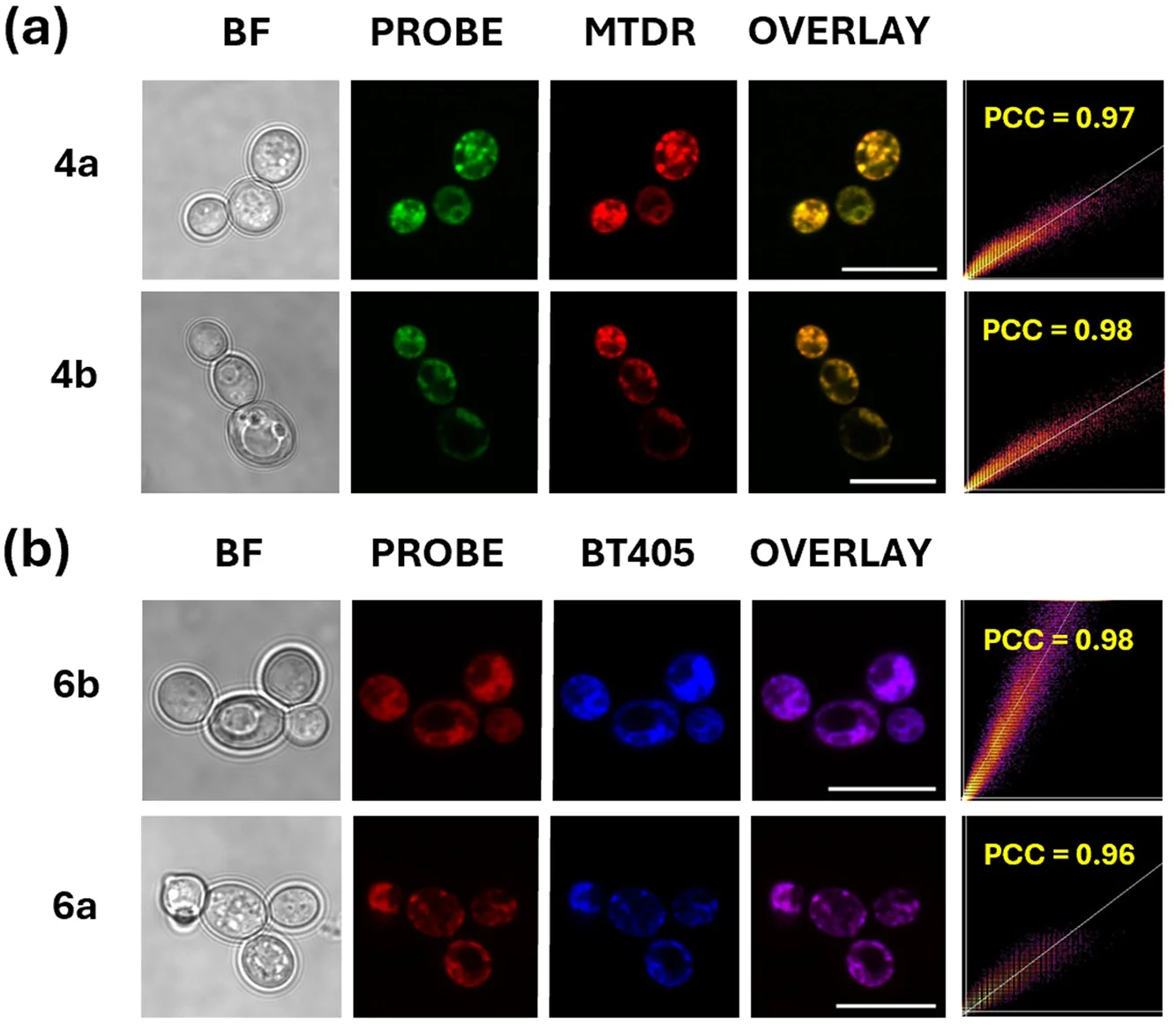
CLSM images of *C. albicans* (CECT 1394) incubated with probes (100 nM) and commercial trackers (100 nM) for 30 min at 37 °C. Images are displayed as bright field (BF), probe channel, tracker channel, merged overlay, and Pearson’s correlation coefficient (PCC). (a) Compounds **4a** and **4b** costained with MTDR. (b) Compounds **6a** and **6b** costained with BT405. For each probe, excitation and emission windows were individually selected based on the probe’s spectra to minimize spectral overlap with the commercial tracker. Scale bar: 10 μm.

As shown in Figure 8, the tested compounds **4a,b** and **6a,b** display clear intracellular fluorescence and strong overlap with the mitochondrial trackers (MTDR or BT405), yielding high Pearson’s correlation coefficients (PCC = 0.96–0.98). These results suggest that the compounds successfully traverse the fungal cell wall barrier and achieve intracellular accumulation, while remaining retained long enough to yield a stable mitochondrial staining pattern, even under conditions in which efflux activity can constrain the accumulation and persistence of many cationic fluorochromes. These findings extend the scope of this probe series beyond human-cell imaging and support its suitability for fungal mitochondrial bioimaging workflows that require robust, quantifiable colocalization with commercial standards.

## Conclusions

In summary, triarylpyrylium and triarylpyridinium (Katritzky-type) salts constitute a well-established class of compact, highly tunable cationic dyes that have been leveraged by many groups across synthetic and bioimaging contexts. In this work, we extend that body of knowledge by moving beyond qualitative targeting descriptors and providing a systematic structure–property–localization framework for a multicolor family of closely related derivatives, in live A549 cancer cells. The library offers flexible spectral coverage for standard confocal imaging, but its central advance is a simple rule set that connects molecular design to intracellular fate: a narrow lipophilicity regime (cLogP ca. 4–6) favors efficient mitochondrial accumulation at low (nanomolar) dosing, while further increases in hydrophobicity enhance aggregation in aqueous media and redirect cellular trafficking toward endo/lysosomal compartments. Practically, this establishes a programmable organelle “address switch” controlled by side-chain engineering rather than core replacement, enabling rational selection of mitochondrial vs lysosomal staining within a common scaffold. Although demonstrated here for the pyrylium/pyridinium platform, this side-chain/cLogP-based strategy may be relevant to other cationic amphiphilic fluorophores; however, its quantitative boundaries are expected to be scaffold-dependent and require experimental validation for each dye family. Selected members also retain robust mitochondrial colocalization in the pathogenic yeast *C. albicans*, supporting applicability beyond mammalian systems. We anticipate that this design map will accelerate the development of functional, environment-responsive probes (including mitochondria-targeted reporters) by providing a predictable starting point for balancing brightness, spectral placement, and subcellular destination.

## Supplementary Material



## Notes

Reagents and instrumentation; synthetic procedures; compound characterization data, including ^1^H/^13^C NMR and HRMS spectra; photophysical characterization methods and data; fluorescence quantum yield and lifetime measurements; cLogP calculations; cell culture, yeast staining, confocal microscopy, PCC analysis, and chloroquine-perturbation protocols; DLS and TEM methods; and additional absorption/emission spectra, solution photographs, lifetime traces, CLSM/line-profile/λ-scan images, MTT cytotoxicity assays, and supplemental references (PDF)

## Abbreviations

ACN: acetonitrile
AIE: aggregation-induced emission
BASHY: boronic-acid-derived salicylidenehydrazone
BF: bright field
BF_4_^–^: tetrafluoroborate
BT405: BioTracker 405
cLogP: calculated partition coefficient
CLSM: confocal laser scanning microscopy
DCM: dichloromethane
D–A–D: donor–acceptor–donor
Δψm: mitochondrial membrane potential
HRMS: high-resolution mass spectrometry
LCs: lipophilic cations
LTDR: LysoTracker Deep Red
MTDR: MitoTracker Deep Red
NMR: nuclear magnetic resonance
PBS: phosphate-buffered saline
PCC: Pearson’s correlation coefficient
PLQY: photoluminescence quantum yield
PYL: pyrylium dyes
PYD: pyridinium dyes
ROI: region of interest
ROS: reactive oxygen species
RNS: reactive nitrogen species
Sr-TICT: steric repulsion-induced twisted intramolecular charge transfer
λ_abs_: absorption maximum
λ_fluo_: fluorescence maximum
Φ_f_: fluorescence quantum yield
τ_f_: fluorescence lifetime
*k*_r_: radiative rate constant
*k*_nr_: nonradiative rate constant
ND: not detected
NC: not calculated.
