# Supplementary material for: A Structure-Programmable Palette of Compact Cationic Dyes Enables Predictable Organelle Addressing and Multicolor Mitochondrial Imaging
Source: Chem Biomed Imaging. 2026 Jun 8;4(10):2269–82. doi: 10.1021/cbmi.6c00056 (PMC13715354; doi:10.1021/cbmi.6c00056)
Supplement: Supplementary file 1 [file im6c00056_si_001.pdf]

A Structure-Programmable Palette of Compact Cationic Dyes Enables Predictable Organelle Addressing and Multicolor Mitochondrial Imaging

Jean C. Neto,[a] Xavier Salom-Roig,[b] Eva Falomir,[a] Rosa de Llanos,[c] Juan F. Miravet,[a] Francisco Galindo*[a]

[a] *Universitat Jaume I de Castellón, Departamento de Química Inorgánica y Orgánica, Avda. Vicente Sos Baynat s/n, 12071 Castellón de la Plana, Spain.*

[b] *Institut des Biomolécules Max Mousseron. Université de Montpellier, CNRS, ENSCM. 1019, Route de Mende, 34293 Montpellier, France.*

[c] *Universitat Jaume I, Unidad Predepartamental de Medicina, Avda. Vicente Sos Baynat s/n, 12071 Castellón de la Plana, Spain.*

* E-mail: francisco.galindo@uji.es

Table of Contents

Reagents and Instruments 4

Figure S1. Molecular library of pyrylium and pyridinium cations studied in this work. 5

Synthesis of 6-(decyloxy)-3,4-dihydronaphthalen-1(2H)-one. 6

Synthesis of Compounds 1a-4a. 6

Synthesis of Compounds 1b-4b. 6

Synthesis of Compounds 5a and 6a 7

Synthesis of Compounds 5b an 6b 7

Synthesis of Compounds 7 and 8. 7

Synthesis of Compounds 9-11a. 7

Synthesis of Compounds 9-11b. 8

Photophysical Characterization of the Compounds. 13

Fluorescence Quantum Yields Measurements (ϕF) 13

Time-resolved fluorescence measurements 14

Toxicity assays 14

Yeast strains and staining. 15

Cell culture and IBIDI preparation. 15

Confocal Microscopy. 15

Calculation of cLogP values. 16

Chloroquine-mediated inhibition of uptake. 16

Dynamic Light Scattering (DLS). 16

Transmission Electron Microscopy (TEM). 17

Figure S2. ^{1}H NMR, ^{13}C NMR (CD_3CN) and mass spectra of compound 1a. 18

Figure S3. ^{1}H NMR, ^{13}C NMR (CD_3CN) and mass spectra of compound 1b. 19

Figure S4. ^{1}H NMR, ^{13}C NMR (CD_3CN) and mass spectra of compound 2a. 20

Figure S5. ^{1}H NMR, ^{13}C NMR (CD_3CN) and mass spectra of compound 2b. 21

Figure S6. ^{1}H NMR, ^{13}C NMR (CD_3CN) and mass spectra of compound 3a. 22

Figure S7. ^{1}H NMR, ^{13}C NMR (CD_3CN) and mass spectra of compound 3b. 23

Figure S8. ^{1}H NMR, ^{13}C NMR (CD_3CN) and mass spectra of compound 4a. 24

iFigure S9. ^{1}H NMR, ^{13}C NMR (CD_3CN) and mass spectra of compound 4b. 25

Figure S10. ^{1}H NMR, ^{13}C NMR (DMSO-d_6) and mass spectra of compound 5a. 26

Figure S11. ^{1}H NMR, ^{13}C NMR (DMSO-d_6) and mass spectra of compound 5b. 27

Figure S12. ^{1}H NMR, ^{13}C NMR (DMSO-d_6) and mass spectra of compound 6a. 28

Figure S13. ^{1}H NMR, ^{13}C NMR (DMSO-d_6) and mass spectra of compound 6b. 29

Figure S14. ^{1}H NMR, ^{13}C NMR (DMSO-d_6) and mass spectra of compound 7. 30

Figure S15. ^{1}H NMR, ^{13}C NMR (methanol-d_4) and mass spectra of compound 8. 31

Figure S16. ^{1}H NMR, ^{13}C NMR (CD_3CN) and mass spectra of compound 9a. 32

Figure S17. H NMR, ^{13}C NMR (CD_3CN) and mass spectra of compound 9b. 33

Figure S18. ^{1}H NMR, ^{13}C NMR (CD_3CN) and mass spectra of compound 10a. 34

Figure S19. ^{1}H NMR, ^{13}C NMR ($CDCl_3$) and mass spectra of compound 10b. 35

Figure S20. ^{1}H NMR, ^{13}C NMR (CD_3CN) and mass spectra of compound 11a. 36

Figure S21. ^{1}H NMR, ^{13}C NMR (CD_3CN) and mass spectra of compound 11b. 37

Table S1. Photophysical properties of compounds in Group B. 38
Table S2. Photophysical properties of compounds in Group C. 38
Figure S22. Time-dependent UV–Vis absorption spectra of compounds 4b, 6b, 7 and 8. 39
Figure S23. Comparative absorption and fluorescence emission of compounds 4b, 6b, 7, and 8 recorded in different solvents. 40
Figure S24. Stokes Shifts of groups A and C. 41
Figure S25. Fluorescence lifetime measurements of compounds 1–6a and 1–6b in dichloromethane. 42
Figure S26. Fluorescence lifetime measurements of compounds 10 and 11 in dichloromethane 43
Figure S27. Fluorescence lifetime measurements of compounds 9-11a and 9-11b in dichloromethane. 43
Figure S28. Photographs of dyes 7 and 8 in DCM under ambient light and UV illumination. 44
Figure S29. Photographs of dyes 9-11a and 9-11b in DCM under ambient light and UV illumination. 44
Figure S30. Normalized absorption and emission of triarylpyrylium and triarylpyridinium dyes (1-6a and 1-6b) in DCM. 45
Figure S31. Normalized absorption and emission of triarylpyrylium and triarylpyridinium dyes (1-6a and 1-6b) in PBS. 46
Figure S32. Normalized absorption and emission of compound 7 in DCM and PBS 47
Figure S33. Normalized absorption and emission of compound 8 in DCM and PBS 47
Figure S34. Normalized absorption and emission of triarylpyrylium and triarylpyridinium dyes (9-11a and 9-11b) in DCM. 48
Figure S35. Normalized absorption and emission of triarylpyrylium and triarylpyridinium dyes (9-11a and 9-11b) in PBS. 48
Figure S36. CLSM, line-profile analysis, and λ-Scan of compounds 2a, 4a, and 6b in A549 cells. 49
Figure S37. MTT cell viability assay performed in A549 cells after 24 h exposure to compounds of group A (1a–6b). 50
Figure S38. MTT cell viability assay performed in A549 cells after 24 h exposure to compounds of group B (7 and 8) 51
Figure S39. MTT cell viability assay performed in A549 cells after 24 h exposure to compounds of group C (9a-11b). 52
Supplemental references 53

Reagents and Instruments. All reagents and solvents were purchased from commercial suppliers and used as received without further purification; solvents employed in spectroscopic measurements were of spectroscopic grade. Reactions requiring anhydrous conditions were carried out in flame-dried glassware under an argon atmosphere using standard syringe techniques. ^{1}H and ^{13}C NMR spectra were recorded on Bruker Avance III HD spectrometers, typically at 400 MHz for ^{1}H and 101 MHz for ^{13}C and, in some cases, at 300 MHz for ^{1}H and 75 MHz for ^{13}C, using $CDCl_3$, CD_3CN or DMSO-d_6 as solvents. Chemical shifts (δ) are reported in ppm relative to residual solvent peaks ($CDCl_3$: 7.26 ppm for ^{1}H and 77.16 ppm for ^{13}C; CD_3CN: 1.94 ppm for ^{1}H and 1.32, 118.26 ppm for ^{13}C; DMSO-d_6: 2.50 ppm for ^{1}H and 39.52 ppm for ^{13}C), and data are presented as follows: chemical shift, multiplicity (s = singlet, d = doublet, t = triplet, q = quartet, m = multiplet), coupling constants (Hz), and integration. Analytical TLC was performed on silica gel 60 F_{254} plates, and column chromatography was carried out on silica gel 60 (63–200 µm) under air pressure. High-resolution mass spectra (HRMS) were acquired by electrospray ionization (ESI) on a Waters Q-Tof Premier mass spectrometer. UV–vis absorption spectra were measured on a Cary 60 UV–vis spectrophotometer using 1 cm path-length quartz cuvettes with a 3 mL sample volume. Microwave-assisted reactions were performed in a Biotage Initiator 60 EXP® reactor, with the temperature monitored by an IR sensor on the outer surface of the reaction vial. Cellular images were collected on a Leica TCS SP8 inverted confocal laser-scanning microscope, and image analysis was performed using ImageJ software.

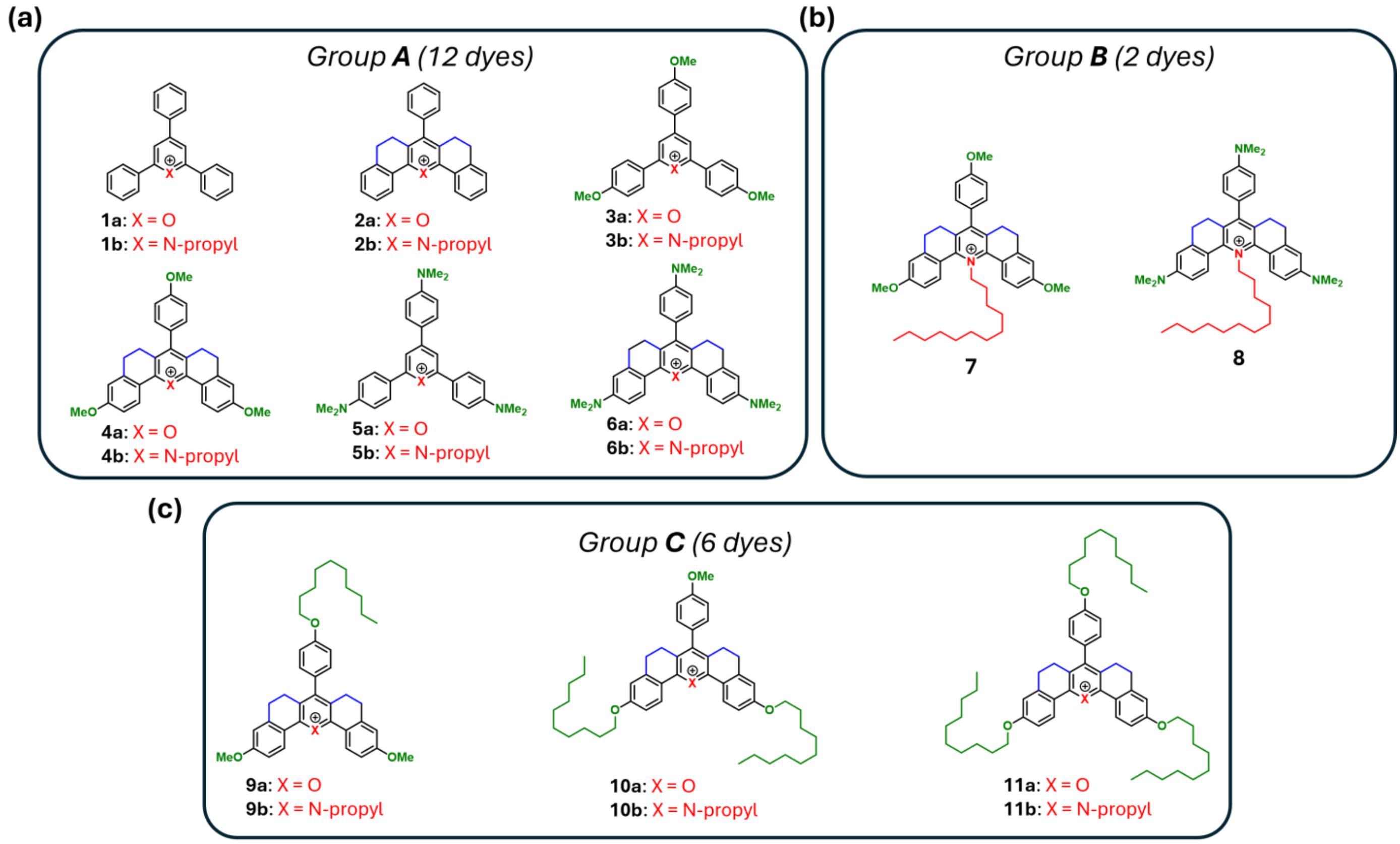

Figure S1. Molecular library of pyrylium and pyridinium cations studied in this work. (a) group **A**, 6 dyes **1-6a** and **1-6b**. (b) group **B**, 2 dyes **7** and **8**. (c) group **C**, 6 dyes **9-11a** and **9-11b.** (BF_4^- is the common anion in all the cases)

Synthesis of 6-(decyloxy)-3,4-dihydronaphthalen-1(2H)-one.

To a suspension of 6-hydroxy-1-tetralone (2.0 g, 12.3 mmol, 1 equiv) and K_2CO_3 (3.41 g, 24.6 mmol, 2 equiv) in acetone (90 mL) was added 1-bromodecane (3.3 ml, 13.6 mmol, 1.1 equiv). The reaction was heated under reflux for 15 hours. Upon completion, the reaction was filtered through a pad of celite and concentrated in vacuo. A purification by column chromatography on silica gel using dichloromethane as the eluent gave 6-(decyloxy)-3,4-dihydronaphthalen-1(2H)-one (3.33 g, 89%) as a white solid. ^{1}H NMR (400 MHz, $CDCl_3$): δ = 7.99 (d, 1H, *J* = 8.7 Hz), 6.80 (dd, 1H, *J* = 8.7, 2.5 Hz), 6.69 (d, 1H, *J* = 2.4 Hz), 4.0 (t, 2H, *J* = 6.6 Hz), 2.91 (t, 2H, *J* = 6.1 Hz), 2.60 (t, 2H, *J* = 6.5 Hz), 2.11 (quint, 2H, *J* = 6.4 Hz), 1.79 (quint, 2H, *J* = 6.7 Hz), 1.48 – 1.41 (m, 2H), 1.36 – 1.27 (m, 12H), 0.88 (t, 3H, *J* = 6.9 Hz). ^{13}C NMR (101 MHz, $CDCl_3$): δ = 197.3, 163.3, 147.0, 129.7, 126.2, 113.5, 113.2, 68.3, 39.0, 32.0, 30.3, 29.6, 29.4, 29.4, 29.2, 26.1, 23.5, 22.8, 14.2. HRMS (ESI+): m/z calcd for $C_{20}H_{31}O_2$: 303.2319; found: 303.2317.

Synthesis of Compounds 1a-4a. Compound **4a** was synthesized according to the previously reported procedure.[1] A mixture of the corresponding para-substituted benzaldehyde (1.0 equiv) and the respective substituted acetophenone or tetralone (2.0 equiv) was added to dry toluene under a nitrogen atmosphere. Then, boron trifluoride diethyl etherate ($BF_3 \cdot Et_2O$, 2.5 equiv) was added dropwise. The reaction mixture was stirred vigorously and heated at 100 °C for 6 hours. After completion, the solvent was removed under reduced pressure, and the crude product was precipitated by addition of diethyl ether, washed with ether, and purified by column chromatography on silica gel using CH_2Cl_2/MeOH (100:1) as eluent to afford the corresponding compound **1a–4a**.

Synthesis of Compounds 1b-4b. Compound **1b** was synthesized according to the previously reported procedure.[1] A solution of the respective compound **1a–4a** (1.0 equiv) in absolute ethanol was treated with *n*-propylamine (1.1 equiv), and the reaction mixture was refluxed for 4 hours. After completion, the solvent was removed under reduced pressure using a rotary evaporator, and the resulting mixture was poured into cold diethyl ether under stirring. The precipitate formed was collected by filtration, washed with diethyl ether, and dried under vacuum to afford compounds **1b–4b**.

Synthesis of Compounds 5a and 6a. A mixture of 4-dimethylaminobenzaldehyde (1.0 equiv) and either 4-dimethylaminoacetophenone or 6-(dimethylamino)-3,4-dihydronaphthalen-1(2H)-one (2.0 equiv) was dissolved in concentrated sulfuric acid (20 equiv) under a nitrogen atmosphere. The reaction mixture was stirred and heated at 100 °C for 4 hours. After completion, the heating was stopped and the reaction was allowed to cool to approximately 50 °C before being quenched with water until a precipitate formed. The temperature was then maintained at 70–80 °C, and a supersaturated aqueous solution of sodium tetrafluoroborate was added to exchange the counterion, which led to a visible color change in both the solution and the precipitate. The mixture was stirred for 30 minutes, cooled to room temperature, and filtered through a porous glass plate. The resulting solid was washed with water to remove excess salts, dissolved in the minimum amount of dichloromethane, and precipitated dropwise into diethyl ether. The solid was collected by filtration and washed repeatedly with diethyl ether to yield the final product, **5a** and **6a**.

Synthesis of Compounds 5b and 6b. A solution of the respective compound **5a** or **6a** (1.0 equiv) in absolute ethanol was treated with *n*-propylamine (10 equiv), and the reaction mixture was refluxed for 4 hours. After completion, the solvent was removed under reduced pressure using a rotary evaporator, and the resulting mixture was poured into cold diethyl ether under stirring. The precipitate formed was collected by filtration, washed with diethyl ether, and purified by flash column chromatography on silica gel using CH_2Cl_2/MeOH (100:1) as eluent to afford compounds **5b** and **6b**.

Synthesis of Compounds 7 and 8. A solution of the respective precursor 4a or 6a (1.0 equiv) in absolute ethanol was treated with N-dodecylamine (10 equiv), and the reaction mixture was refluxed for 4 hours. After completion, the solvent was removed under reduced pressure using a rotary evaporator, and the resulting residue was poured into cold diethyl ether under stirring. The precipitate formed was collected by filtration, washed with diethyl ether, and purified by flash column chromatography on silica gel using CH_2Cl_2/MeOH (100:1) as eluent to afford compounds **7** and **8**.

Synthesis of Compounds 9-11a. To a dry flask under argon atmosphere, a mixture of the substituted 1-tetralone derivative (2.0 equiv) and the corresponding para-substituted benzaldehyde (1.0 equiv) was added. Then, boron trifluoride diethyl etherate ($BF_3 \cdot Et_2O$, 2.4 equiv) was added dropwise. The reaction mixture was

stirred vigorously and heated at 100 °C for a period of 6 or 22 hours depending on the substrates (22 hours for compounds **9a** and **10a**, and 6 hours for compound **11a**). After completion, the reaction mixture was cooled to room temperature and purified by flash column chromatography on silica gel using CH_2Cl_2/MeOH (100:1) as eluent. The resulting solid was precipitated in diethyl ether and washed with acetone.

Synthesis of Compounds 9-11b. A mixture of the corresponding precursor compound (**7a**, **8a** and **9a**, 1.0 equiv) and *n*-propylamine (12 equiv) in 2 mL of ethanol was heated in a microwave oven at 80 °C for 1 hour. After completion, the solvents were evaporated under reduced pressure. The crude reaction mixture was purified by flash column chromatography on silica gel using a mixture of CH_2Cl_2/MeOH (100:1) as eluent. The resulting solid was precipitated in diethyl ether to obtain the final compound.

Compound 1a. Starting materials: 1.42 g of benzaldehyde, 3.22 g of acetophenone. After precipitation: yellow solid, 2.50 g, yield of 47%. ^{1}H NMR (400 MHz, CD_3CN) δ 8.72 (s, 2H), 8.43 (d, *J* = 7.2 Hz, 4H), 8.31 (d, *J* = 7.2 Hz, 2H), 7.90 – 7.84 (m, 3H), 7.81 – 7.74 (m, 6H). ^{13}C NMR (101 MHz, CD_3CN) δ 171.94, 167.46, 136.39, 136.26, 133.88, 131.05, 131.04, 130.59, 129.99, 129.72, 116.78. HRMS (ESI+): m/z calcd for $C_{23}H_{17}O^+$: 309.1274; found: 309.1285.

Compound 1b. Starting materials: 1.00 g of **1a**, 0.164 g of *n*-propylamine. After precipitation: white solid, 0.90 g, yield of 83%. ^{1}H NMR (400 MHz, CD_3CN) δ 8.18 (s, 2H), 8.02 – 7.95 (m, 2H), 7.75 – 7.57 (m, 13H), 4.38 – 4.16 (m, 2H), 1.53 – 1.33 (m, 2H), 0.39 (t, *J* = 7.4 Hz, 3H). ^{13}C NMR (101 MHz, CD_3CN) δ 157.46, 156.21, 134.63, 133.93, 133.36, 132.06, 130.70, 130.21, 129.93, 129.31, 127.70, 57.18, 24.07, 10.78. HRMS (ESI+): m/z calcd for $C_{26}H_{24}N^+$: 350.1903; found: 350.1914.

Compound 2a. Starting materials: 2.0 g of benzaldehyde, 5.51 g of α-tetralone. After precipitation: yellow solid, 4.80 g, yield of 52%. ^{1}H NMR (400 MHz, CD_3CN) δ 8.36 (d, *J* = 7.8 Hz, 2H), 7.78 – 7.57 (m, 7H), 7.49 (d, *J* = 7.6 Hz, 2H), 7.43 – 7.31 (m, 2H), 3.06 (dd, *J* = 8.7, 6.4 Hz, 4H), 2.89 (dd, *J* = 8.5, 6.3 Hz, 4H). ^{13}C NMR (101 MHz, CD_3CN) δ 167.24, 166.09, 142.92, 136.43, 133.75, 131.67, 131.52, 130.33, 130.13, 129.37, 128.05, 127.18, 126.79, 26.88, 25.17. HRMS (ESI+): m/z calcd for $C_{27}H_{21}O^+$: 361.1587; found: 361.1594.

Compound 2b. Starting materials: 2.0 g **2a**, 0.36 g of *n*-propylamine. After precipitation: pale yellow solid, 1.44 g, yield of 72%. ^{1}H NMR (400 MHz, CD_3CN) δ 8.15 (d, J = 9.1 Hz, 2H), 7.74 – 7.50 (m, 9H), 7.45 (s, 1H), 7.19 (s, 1H), 5.23 (t, J = 6.8 Hz, 2H), 2.88 (t, J = 6.8 Hz, 4H), 2.81 (s, 2H), 2.38 (s, 2H), 1.31 (h, J = 7.2 Hz, 2H), 0.40 (t, J = 7.3 Hz, 3H). ^{13}C NMR (101 MHz, CD_3CN) δ 156.32, 154.22, 142.75, 138.80, 136.17, 133.62, 130.32, 130.21, 130.01, 129.63, 129.59, 128.50, 67.05, 28.45, 27.12, 24.50, 10.46. HRMS (ESI+): m/z calcd for $C_{30}H_{28}N^+$: 402.2216; found: 402.2220.

Compound 3a. Starting materials: 1.50 g of 4-methoxybenzaldehyde, 3.31 g of 4-methoxyacetophenone. After precipitation: red solid, 2.85 g, yield of 54%. ^{1}H NMR (400 MHz, CD_3CN) δ 8.31 (d, J = 2.6 Hz, 4H), 8.30 (d, J = 2.2 Hz, 2H), 8.28 (d, J = 2.2 Hz, 1H), 8.26 (d, J = 2.1 Hz, 1H), 7.24 (d, J = 1.7 Hz, 3H), 7.22 (d, J = 1.7 Hz, 3H), 3.97 (s, 3H), 3.97 (s, 6H). ^{13}C NMR (101 MHz, CD_3CN) δ 169.73, 166.81, 166.30, 163.79, 132.80, 131.57, 125.79, 122.39, 116.55, 116.46, 112.35, 56.91, 56.85. HRMS (ESI+): m/z calcd for $C_{26}H_{23}O_4^+$: 399.1591; found: 399.1598.

Compound 3b. Starting materials: 1.50 g of **3a**, 0.27 g of *n*-propylamine. After precipitation: yellow solid, 1.10 g, yield of 68%. ^{1}H NMR (400 MHz, CD_3CN) δ 8.04 (s, 2H), 8.02 – 7.94 (m, 2H), 7.68 – 7.56 (m, 4H), 7.23 – 7.16 (m, 4H), 7.16 – 7.09 (m, 2H), 4.35 – 4.27 (m, 2H), 3.91 (s, 5H), 3.89 (s, 3H), 1.46 – 1.32 (m, 2H), 0.42 (t, J = 7.4 Hz, 3H). ^{13}C NMR (101 MHz, CD_3CN) δ 164.42, 162.60, 157.34, 155.08, 131.68, 131.16, 126.47, 126.37, 126.20, 116.14, 115.52, 56.92, 56.48, 56.37, 23.86, 10.76. HRMS (ESI+): m/z calcd for $C_{29}H_{30}NO_3^+$: 440.2220; found: 440.2224.

Compound 4a. Starting materials: 1.00 g 4-methoxybenzaldehyde, 2.60 g of 6-methoxy-1-tetralone. After precipitation: red solid, 1.10 g, yield of 28%. ^{1}H NMR (400 MHz, CD_3CN) δ 8.24 (d, J = 8.8 Hz, 2H), 7.30 (d, J = 8.9 Hz, 2H), 7.18 (d, J = 8.9 Hz, 2H), 7.11 (dd, J = 8.8, 2.6 Hz, 2H), 7.00 (d, J = 2.6 Hz, 2H), 3.94 (s, 6H), 3.90 (s, 3H), 2.99 (t, J = 7.5 Hz, 4H), 2.89 – 2.80 (m, 4H). ^{13}C NMR (75 MHz, DMSO-d_6) δ 164.67, 163.12, 163.06, 160.66, 144.21, 129.71, 128.36, 127.06, 124.66, 118.54, 114.86, 114.54, 113.72, 56.03, 55.46, 26.21, 24.08. HRMS (ESI+): m/z calcd for $C_{30}H_{27}O_4^+$: 451.1904; found: 451.1913.

Compound 4b. Starting materials: 0.80 g of 4**a**, 0.14 g of *n*-propylamine. After precipitation: yellow solid, 0.50 g, yield of 59%. ^{1}H NMR (400 MHz, CD_3CN) δ 8.05 (d, J = 8.7 Hz, 2H), 7.08 (dd, J = 8.8, 2.7 Hz, 6H),

7.04 (d, J = 2.6 Hz, 3H), 5.09 (t, J = 6.7 Hz, 2H), 3.92 (s, 6H), 3.88 (s, 3H), 2.83 (t, J = 6.6 Hz, 6H), 2.34 (s, 2H), 1.37 – 1.24 (m, 2H), 0.40 (t, J = 7.3 Hz, 3H). ^{13}C NMR (101 MHz, CD_3CN) δ 163.82, 161.29, 155.47, 153.74, 145.22, 136.98, 131.83, 130.39, 128.34, 122.57, 115.46, 114.49, 114.31, 67.12, 56.58, 56.20, 28.94, 26.90, 24.08, 10.48. HRMS (ESI+): m/z calcd for $C_{33}H_{34}NO_3^+$: 492.2533; found: 492.2533.

Compound 5a. Starting materials: 2.50 g 4-(dimethylamino)benzaldehyde, 5.47 g of 4-dimethylaminoacetophenone. After precipitation: purple solid, 3.30 g, yield of 38%. ^{1}H NMR (400 MHz, DMSO-d_6) δ 8.29 (d, J = 8.4 Hz, 2H), 8.15 – 8.07 (m, 6H), 6.84 (d, J = 8.5 Hz, 6H), 3.15 (s, 6H), 3.10 (s, 12H). ^{13}C NMR (101 MHz, DMSO-d_6) δ 165.03, 156.87, 154.20, 153.38, 131.39, 129.10, 118.61, 115.56, 111.99, 111.83, 105.14, 39.61. HRMS (ESI+): m/z calcd for $C_{29}H_{32}N_3O^+$: 438.2540; found: 438.2540.

Compound 5b. Starting materials: 1.50 g of **5a**, 0.84 g of *n*-propylamine. After precipitation: brownish solid, 1.00 g, yield of 66%. ^{1}H NMR (400 MHz, DMSO-d_6) δ 8.23 (d, J = 7.9 Hz, 1H), 8.09 (d, J = 9.0 Hz, 2H), 7.96 (d, J = 6.8 Hz, 2H), 7.65 (d, J = 8.8 Hz, 3H), 7.57 (d, J = 8.3 Hz, 1H), 6.91 (d, J = 8.9 Hz, 4H), 6.82 (d, J = 9.2 Hz, 2H), 6.74 (d, J = 8.6 Hz, 1H), 4.41 (t, J = 7.2 Hz, 2H), 3.06 (s, 6H), 3.04 (s, 12H), 1.26 (dd, J = 13.9, 7.5 Hz, 2H), 0.37 (t, J = 7.4 Hz, 3H). ^{13}C NMR (75 MHz, DMSO-d_6) δ 156.39, 151.37, 130.43, 129.92, 121.51, 120.59, 112.06, 111.69, 39.66, 29.34, 22.02, 10.31. HRMS (ESI+): m/z calcd for $C_{32}H_{39}N_4^+$: 479.3169; found: 479.3174.

Compound 6a. Starting materials: 2.00 g 4-(dimethylamino)benzaldehyde, 5.07 g of 6-(dimethylamino)-3,4-dihydronaphthalen-1(2H)-one. After precipitation: blue solid, 1.98 g, yield of 28%. ^{1}H NMR (400 MHz, DMSO-d_6) δ 8.01 (d, J = 9.0 Hz, 2H), 7.25 (dd, 2H), 6.95 – 6.79 (m, 4H), 6.71 (s, 2H), 3.12 (s, 12H), 3.02 (s, 6H), 2.91 – 2.82 (m, 4H), 2.80 – 2.72 (m, 4H). ^{13}C NMR (101 MHz, DMSO-d_6) δ 173.53, 161.46, 153.89, 150.98, 142.70, 129.72, 127.13, 122.66, 119.69, 113.12, 111.46, 110.32, 28.76, 26.93, 24.28. HRMS (ESI+): m/z calcd for $C_{33}H_{39}N_3O^+$: 490.2853; found: 490.2861.

Compound 6b. Starting materials: 1.00 g of **6a**, 0.56 g of *n*-propylamine. After precipitation: red solid, 0.56 g, yield of 52%. ^{1}H NMR (400 MHz, DMSO-d_6) δ 8.13 (d, J = 9.0 Hz, 2H), 7.28 – 7.00 (m, 2H), 6.98 – 6.63 (m, 7H), 4.95 (q, J = 7.6 Hz, 2H), 3.09 (s, 12H), 2.99 (s, 6H), 2.88 – 2.66 (m, 8H), 1.28 (q, J = 6.7 Hz, 2H), 0.35 (t, J = 7.3 Hz, 3H). ^{13}C NMR (101 MHz, DMSO-d_6) δ 152.65, 152.12, 151.96, 150.25, 142.65, 132.65,

130.33, 129.12, 122.16, 116.28, 111.98, 110.19, 110.01, 65.57, 29.13, 28.26, 25.65, 22.14, 9.96. HRMS (ESI+): m/z calcd for $C_{36}H_{43}N_4^+$: 531.3482; found: 531.3485.

Compound 7. Starting materials: 0.5 g of **4a**, 0.43 g of *n*-dodecylamine. After precipitation: yellow solid, 0.36 g, yield of 52%. ^{1}H NMR (300 MHz, DMSO-d_6) δ 8.42 (d, *J* = 8.5 Hz, 2H), 7.37 (s, 1H), 7.27 – 7.05 (m, 7H), 5.14 (t, *J* = 6.6 Hz, 2H), 3.91 (s, 6H), 3.85 (s, 3H), 2.96 – 2.67 (m, 6H), 2.40 (d, *J* = 16.8 Hz, 2H), 1.22 (d, *J* = 16.1 Hz, 18H), 0.84 (dt, *J* = 7.0, 3.3 Hz, 3H), 0.73 – 0.58 (m, 2H). ^{13}C NMR (75 MHz, DMSO-d_6) δ 162.25, 159.71, 153.60, 151.95, 143.75, 135.34, 131.23, 129.53, 126.94, 121.25, 114.56, 113.42, 113.30, 63.72, 55.72, 55.31, 31.26, 28.92, 28.83, 28.68, 28.62, 28.32, 27.68, 25.76, 24.62, 22.05, 13.92. HRMS (ESI+): m/z calcd for $C_{42}H_{52}NO_3^+$: 618.3942; found: 618.3934.

Compound 8. Starting materials: 0.5 g of **6a**, 1.76 g of *n*-dodecylamine. After precipitation: red solid, 0.34 g, yield of 48% ^{1}H NMR (400 MHz, MeOD) δ 7.93 (d, *J* = 8.8 Hz, 2H), 7.18 – 6.97 (m, 2H), 6.86 (dd, *J* = 9.0, 2.8 Hz, 4H), 6.72 (d, *J* = 2.8 Hz, 2H), 5.09 (t, *J* = 6.5 Hz, 2H), 3.13 (s, 12H), 3.03 (s, 6H), 2.93 – 2.69 (m, 6H), 2.32 (s, 2H), 1.42 – 0.98 (m, 17H), 0.89 (t, *J* = 7.0 Hz, 3H), 0.75 (q, *J* = 7.7 Hz, 2H). ^{13}C NMR (101 MHz, MeOD) δ 155.03, 154.28, 154.24, 152.38, 144.39, 134.65, 131.23, 130.14, 124.08, 118.00, 111.76, 111.23, 65.47, 49.50, 49.28, 49.07, 40.51, 40.16, 33.09, 30.72, 30.62, 30.43, 30.19, 30.12, 30.04, 29.45, 27.16, 26.36, 23.72, 14.44. HRMS (ESI+): m/z calcd for $C_{45}H_{61}N_4^+$: 657.4891; found: 657.4888.

Compound 9a. Starting materials: 1.26 mL 4-decyloxybenzaldehyde, 1.60 g of 6-methoxy-1-tetralone. After precipitation: brown solid, 0.81 g, yield of 26%. ^{1}H NMR (CD_3CN, 400 MHz): δ = 8.24 (d, 2H, *J* = 8.8 Hz), 7.27 (d, 2H, *J* = 8.8 Hz), 7.14 (d, 2H, *J* = 8.8 Hz), 7.11 (dd, 1H, *J* = 8.8, 3.5 Hz), 7.01 (d, 2H, *J* = 2.4 Hz), 4.08 (t, 2H, *J* = 6.5 Hz), 3.94 (s, 6H), 3.00 – 2.96 (m, 4H), 2.87 – 2.83 (m, 4H), 1.84 – 1.77 (m, 2H), 1.52-1.30 (m, 14H), 0.89 (t, 3H, *J* = 6.8 Hz). ^{13}C NMR (CD_3CN, 101 MHz): δ = 166.3, 165.0, 164.8, 161.7, 145.5, 130.4,129.3, 128.5, 125.7, 119.7, 115.9, 115.5, 114.9, 69.2, 56.8, 32.6, 30.3, 30.0, 29.8, 27.4, 26.6, 25.2, 23.4, 14.4. HRMS (ESI+): m/z calcd for $C_{39}H_{45}O_4$: 577.3312; found: 577.3312.

Compound 9b. Starting materials: 75 mg **7a**, 0.11 mL of *n*-propylamine. After precipitation: yellow solid, 34 mg, yield of 43%. ^{1}H NMR (CD_3CN, 400 MHz): δ = 8.04 (d, 2H, *J* = 8.7 Hz), 7.34-7.11 (m, 4H), 7.07 (dd, 2H, *J* = 8.7, 2.7 Hz) 7.04 (d, 2H, *J* = 2.6 Hz), 5.08 (t, 2H, *J* = 6.7 Hz), 4.06 (t, 2H, *J* = 6.5 Hz), 3.91 (s, 9H),

2.85 – 2.74 (m, 6H), 2.40 – 2.27 (m, 2H), 1.83 – 1.76 (m, 2H), 1.53 – 1.27 (m, 16H), 0.89 (t, 3H, *J* = 6.8 Hz), 0.39 (t, 3H, *J* = 7.3 Hz). ^{13}C NMR (CD_3CN, 101 MHz): δ = 163.8, 160.8, 155.5, 153.7, 145.2, 137.0, 131.8, 130.4, 128.2, 122.6, 115.9, 114.5, 114.3, 69.0, 67.1, 56.6, 32.6, 30.3, 30.1, 29.9, 29.0, 26.9, 26.7, 24.1, 23.4, 14.4, 10.5. HRMS (ESI+): m/z calcd for $C_{42}H_{52}NO_3$: 618.3942; found: 618.3939.

Compound 10a. Starting materials: 0.45 mL *p*-anisaldehyde, 2.24 g of 6-(decyloxy)-3,4-dihydronaphthalen-1(2H)-one. After precipitation: brown solid, 0.51 g, yield of 17%. ^{1}H NMR (CD_3CN, 400 MHz): δ = 8.16 (d, 2H, *J* = 8.8 Hz), 7.30 (d, 2H, *J* = 8.5 Hz), 7.18 (d, 2H, *J* = 8.5 Hz), 7.03 (dd, 2H, *J* = 8.7, 1.5 Hz), 6.92 (s, 2H), 4.09 (t, 4H, *J* = 6.4 Hz), 3.90 (s, 3H), 2.97 – 2.93 (m, 4H), 2.84 – 2.80 (m, 4H), 1.81 – 1.75 (m, 4H), 1.45 – 1.29 (m, 28H), 0.89 – 0.87 (m, 6H). ^{13}C NMR (CD_3CN, 101 MHz): δ = 165.9, 164.9, 164.7, 162.2, 145.5, 130.4,129.3, 128.3, 125.9, 119.5, 116.0, 115.5, 115.4, 69.9, 56.4, 32.7, 30.3, 30.1, 30.0, 29.7, 27.5, 26.6, 25.2, 23.4, 14.4. HRMS (ESI+): m/z calcd for $C_{48}H_{63}O_4$: 703.4721; found: 703.4724.

Compound 10b. Starting materials: 82 mg **8a**, 0.10 mL of *n*-propylamine. After precipitation: yellow solid, 19 mg, yield of 22%. ^{1}H NMR ($CDCl_3$, 400 MHz): δ = 8.33 (d, 2H, *J* = 8.8 Hz), 7.29 – 7.00 (m, 4H), 7.16 (dd, 2H, *J* = 8.8, 2.5 Hz), 6.84 (d, 2H, *J* = 2.5 Hz), 5.24 (t, 2H, *J* = 6.8 Hz), 4.06 (t, 2H, *J* = 6.5 Hz), 3.89 (s, 3H), 2.88 – 2.75 (m, 6H), 2.38 – 2.23 (m, 2H), 1.83 – 1.76 (m, 4H), 1.49 – 1.27 (m , 30H), 0.89 – 0.86 (m, 6H), 0.42 (t, 3H, *J* = 7.3 Hz). ^{13}C NMR ($CDCl_3$, 101 MHz): δ = 162.7, 160.4, 154.1, 153.2, 142.9, 135.4, 132.2, 129.2, 127.3, 121.2, 115.0, 114.8, 114.0, 68.7, 66.6, 55.6, 32.0, 29.7, 29.5, 29.5, 29.3, 28.7, 26.4, 26.1, 23.4, 22.8, 14.3, 10.5. HRMS (ESI+): m/z calcd for $C_{51}H_{70}NO_3$: 744.5350; found: 744.5354.

Compound 11a. Starting materials: 1.20 g 4-decyloxybenzaldehyde, 2.80 g of 6-(decyloxy)-3,4-dihydronaphthalen-1(2H)-one. After precipitation: brown solid, 0.84 g, yield of 20%. ^{1}H NMR (CD_3CN, 400 MHz): δ = 8.22 (d, 2H, *J* = 8.8 Hz), 7.30 (d, 2H, *J* = 8.7 Hz), 7.17 (d, 2H, *J* = 8.7 Hz), 7.09 (dd, 2H, *J* = 8.8, 2.3 Hz), 6.98 (d, 2H, *J* = 1.9 Hz), 4.16 – 4.09 (m, 6H), 3.00 – 2.97 (m, 4H), 2.87 – 2.84 (m, 4H), 1.87 – 1.79 (m, 6H), 1.52 – 1.33 (m, 42H), 0.93 – 0.90 (m, 9H). ^{13}C NMR ($CDCl_3$, 101 MHz): δ = 165.0, 164.3, 164.0, 160.8, 144.2, 129.5, 128.4, 127.3, 124.8, 118.8, 115.3, 114.5, 68.9, 68.5, 32.1, 29.7, 29.5, 29.5, 29.3, 29.2, 27.2, 26.2, 26.1, 24.7, 22.8, 14.3. HRMS (ESI+): m/z calcd for $C_{57}H_{81}O_4$: 829.6129; found: 829.6138.

Compound 11b. Starting materials: 96 mg **9a**, 0.11 mL of *n*-propylamine. After precipitation: yellow solid, 12 mg, yield of 12%. ^{1}H NMR (CD_3CN, 400 MHz): δ = 8.06 (d, 2H, *J* = 8.7 Hz), 7.38 – 7.12 (m, 4H), 7.08 (dd, 2H, *J* = 8.7, 2.6 Hz), 7.04 (d, 2H, *J* = 2.5 Hz), 5.11 (t, 2H, *J* = 6.7 Hz), 4.15 (t, 2H, *J* = 6.5 Hz), 4.09 (t, 2H, *J* = 6.5 Hz), 2.87 – 2.81 (m, 6H), 2.43 – 2.29 (m, 2H), 1.87 – 1.80 (m, 6H), 1.53 – 1.33 (m, 44H), 0.94 – 0.91 (m, 9H), 0.43 (t, 3H, *J* = 7.3 Hz). ^{13}C NMR ($CDCl_3$, 101 MHz): δ = 162.7, 160.0, 154.2, 153.2, 142.9, 135.4, 132.2, 129.1, 127.0, 121.2, 115.4, 114.8, 114.0, 68.7, 68.4, 66.6, 32.0, 29.7, 29.5, 29.4, 29.3, 29.2, 28.7, 26.4, 26.2, 26.1, 23.4, 22.8, 14.2, 10.5. HRMS (ESI+): m/z calcd for $C_{60}H_{88}NO_3$: 870.6759; found: 870.6764.

Photophysical Characterization of the Compounds. Stock solutions of the compounds were initially prepared in acetonitrile at a concentration of 20 mM. For UV–Vis absorption measurements, aliquots of these stock solutions were diluted to final concentrations of 15 or 30 μM in acetonitrile, dichloromethane, or PBS buffer (10 mM, pH 7.4). Fluorescence emission spectra were recorded after adjusting the sample absorbance to approximately 0.1 at the excitation wavelength in order to minimize primary inner-filter effects. The excitation wavelength (λ_{exc}) was set at the absorption maximum of each individual compound.

Fluorescence Quantum Yield Measurements (ϕF). Fluorescence quantum yields (ϕ_F) were determined in acetonitrile, dichloromethane, and PBS. Quinine sulfate (ϕ_F = 0.55 in 0.5 M H_2SO_4),[2] fluorescein (ϕ_F = 0.97 in basic methanol),[3] and Oxazine-170 (ϕ_F = 0.63 in methanol)[4] were used as initial reference standards. For each measurement, the absorbance of the reference and of the sample was matched at the excitation wavelength. Quantum yields were then calculated according to Equation 1:

$$\phi_F = \phi_R \cdot (A_R \cdot F_C / A_C \cdot F_R) \cdot (n_C^2 / n_R^2) \qquad \text{Equation 1}$$

where ϕ_F denotes the fluorescence quantum yield, *F* corresponds to the integrated emission intensity, *A* represents the absorbance at the excitation wavelength, and *n* is the refractive index of the medium. The subscripts *R* and *C* refer to the reference standard and the sample under investigation, respectively.

Time-resolved fluorescence measurements. Analyses were carried out using time-correlated single-photon counting (TCSPC) on a Horiba IBH-5000U. Samples were excited with pulsed NanoLED sources operating at different wavelengths (304, 372, 464 and 560 nm), selected according to the absorption characteristics of each compound. The full width at half maximum (FWHM) of the excitation pulses was in the sub-nanosecond range, and the repetition rate for all measurements was set to 100 kHz. The instrument response function was analyzed by iterative reconvolution using the IBH DAS6 decay analysis software. Fluorescence decay curves were fitted to the exponential model shown in Equation 2, where *I(t)* is the time-dependent emission intensity, τ_i are the fluorescence lifetimes, and α_i are the pre-exponential factors associated with each decay component when the emission is not monoexponential.

$$I(t) = \sum_i \alpha_i \exp\left(-\frac{t}{\tau_i}\right)$$ Equation 2

Toxicity assays. Cell viability was evaluated using the MTT assay following standard methodologies reported in the literature,[5] with minor adjustments. Cells were plated in 96-well plates at an initial density of approximately 5×10^4 cells per well and maintained in their respective culture medium at 37 °C, 5% CO_2 for 24h to allow proper attachment and stabilization. After this initial incubation, varying concentrations of the test compounds were added, and the cells were exposed to the treatments for an additional 4 hours under identical conditions. Subsequently, 10 µL of an MTT stock solution (5 mg/mL in PBS) was introduced into each well, followed by a 3-h incubation period to enable viable, metabolically active cells to reduce MTT into insoluble formazan crystals. The supernatant was then removed, and the resulting formazan was solubilized by adding 100 µL of dimethyl sulfoxide (DMSO). The plates were gently agitated to ensure complete dissolution, and absorbance was recorded at 570 nm with a reference wavelength of 630 nm using a Multiskan FC microplate reader. The measured absorbance values correlated directly with the number of viable cells, enabling quantification of cytotoxic effects of the evaluated compounds.

Yeast strains and staining. Yeast strains used for CLSM imaging were first revived from glycerol stocks by streaking onto Sabouraud Dextrose Agar (SDA) plates and incubating at 37 °C for 24 hours. A single colony was then used to inoculate 1× Sabouraud Broth, followed by incubation at 37 °C for 24 hours under constant agitation. Cells were collected by centrifugation and washed three times with phosphate-buffered saline (PBS, pH 7.4) to remove any remaining culture medium. The final suspension was adjusted to 1×10^6 cells/mL in PBS, and aliquots were transferred into microtubes for subsequent treatments.[6] Each sample was incubated with the fluorescent probes at 100 nM and MitoTracker Deep Red FM at 100 nM. After 30 min at 37 °C, cells were pelleted at 10,000 rpm for 5 min and washed three times with PBS to eliminate any non-internalized dye. For CLSM imaging, the stained cells were resuspended in 50 µL of PBS, and a small volume of this suspension was deposited onto a microscope slide containing a pre-applied mounting medium (90% glycerol in PBS).[7–9]

Cell culture and IBIDI preparation. The preparation of IBIDI plates for confocal microscopy followed the procedure previously described.[5] Cells were seeded in IBIDI 4-well plates and allowed to adhere for 24 hours at 37 °C in a humidified atmosphere containing 5% CO_2. After this initial period, the culture medium was removed, and the cells were gently washed three times with phosphate-buffered saline (PBS, pH 7.4) to eliminate any residual medium. Subsequently, the staining solutions were added to each well, consisting of the mitochondrial probe (0.1 µM, 30 min) and either MitoTracker Deep Red FM or BioTracker 405 (100 nM, 30 min). The cells were incubated with the corresponding staining mixture at 37 °C for the specified period. After incubation, the solutions were removed, and the cells were washed three additional times with PBS to eliminate any unbound dyes. Finally, the wells were filled with culture medium lacking FluoroBrite to minimize background fluorescence during confocal imaging.

Confocal Microscopy. Images were acquired using a Leica TCS SP8 confocal microscope equipped with a 60× oil-immersion objective. For each probe, the excitation laser line and the emission detection window were selected individually on the basis of their absorption and emission spectra, and adjusted to avoid any spectral overlap with the commercial trackers. MitoTracker Deep Red FM was excited at 633 nm, whereas BioTracker

405 was excited at 405 nm. Separate images were recorded for each channel (BioTracker 405 or MitoTracker Deep Red, and the probes) in the A549 cells, using sequential acquisition to minimize bleed-through between fluorophores. Pearson's correlation coefficient (PCC) values were calculated from multiple confocal images using a total of 10 individual cells for each probe. Cells were selected from different regions of the images to ensure representative sampling and to minimize potential bias arising from local heterogeneity. The results are reported as mean ± standard deviation (SD).

Calculation of cLogP values. The lipophilicity (cLogP) of the compounds was estimated using the SwissADME web server.[10] Calculations were performed using canonical SMILES as input, and the reported values correspond to the consensus LogP, obtained as the average of five predictive models implemented in SwissADME (XlogP3, WlogP, MlogP, iLOGP, and Silicos-IT). All calculations were carried out using the default settings.

Chloroquine-mediated inhibition of uptake. The assay was adapted from a previously reported chloroquine-based nanoparticle uptake protocol.[11] Cells were seeded in IBIDI 4-well plates and allowed to adhere for 24 h at 37 °C in a humidified atmosphere containing 5% CO_2. The culture medium was then removed, and the cells were gently washed with PBS. For the chloroquine-treated condition, cells were incubated with chloroquine phosphate (200 μM) for 30 min at 37 °C in FBS-free culture medium. Subsequently, the fluorescent probe was added to a final concentration of 100 nM, and the cells were incubated for an additional 30 min under the same conditions. After incubation, the staining solution was removed, the cells were washed with PBS, and fresh culture medium without FluoroBrite was added before imaging. Confocal images were acquired using a Leica TCS SP8 confocal microscope with 488 nm laser excitation at 50% laser power. Fluorescence emission was collected between 550 and 650 nm using 850% gain.

Dynamic Light Scattering (DLS). Samples were prepared by dissolving each probe in 10 mM PBS, pH 7.4 to a final concentration of 50 μM. Samples were introduced into 3 mL disposable PMMA cuvettes (10 mm optical path length). The suspensions were analyzed using a Zetasizer Nano-ZS90 instrument (Malvern

Instruments, UK). Measurements were carried out at 25 °C, applying the automatic optimization of beam focusing and attenuation for each sample. Particle size distributions were obtained from three independent measurements, and the results are reported as average hydrodynamic diameter and polydispersity index (PDI). Unless otherwise stated, the samples were analyzed without filtration in order to preserve the native population of aggregates present in solution.

Transmission Electron Microscopy (TEM). Samples were prepared in 10 mM PBS, pH 7.4, at a probe concentration of 50 μM. A drop of each freshly prepared suspension was deposited onto Formvar/carbon-coated copper grids and allowed to adsorb briefly. The excess liquid was carefully removed by capillary action using filter paper, and the deposition/removal step was repeated twice. No staining or contrast agent was applied to the samples. The grids were then air-dried at room temperature before observation. TEM images were acquired using a JEOL JEM-2100 transmission electron microscope equipped with a LaB_6 thermionic electron gun, operated at 100 kV, and a Gatan Orius high-resolution CCD camera.

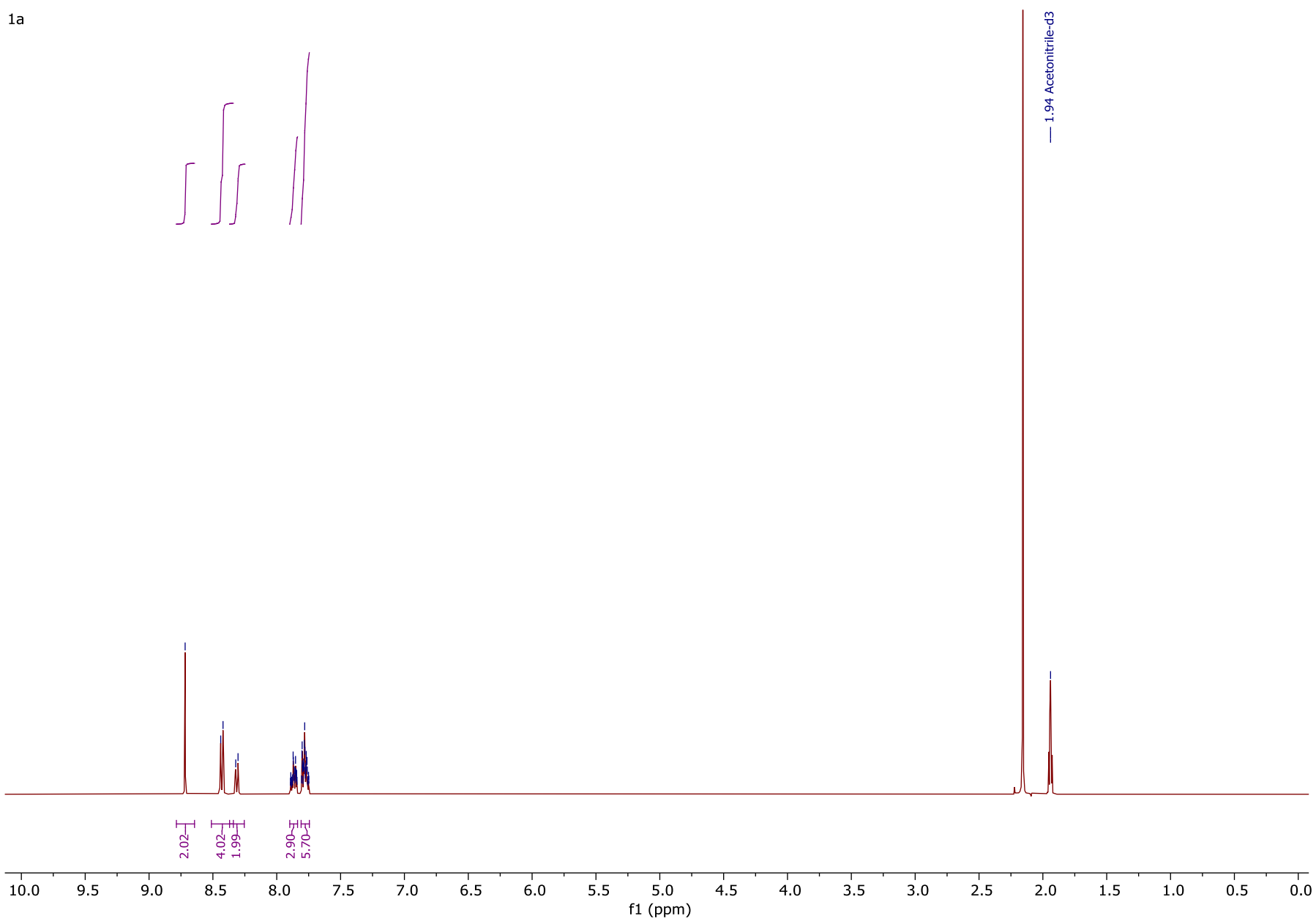

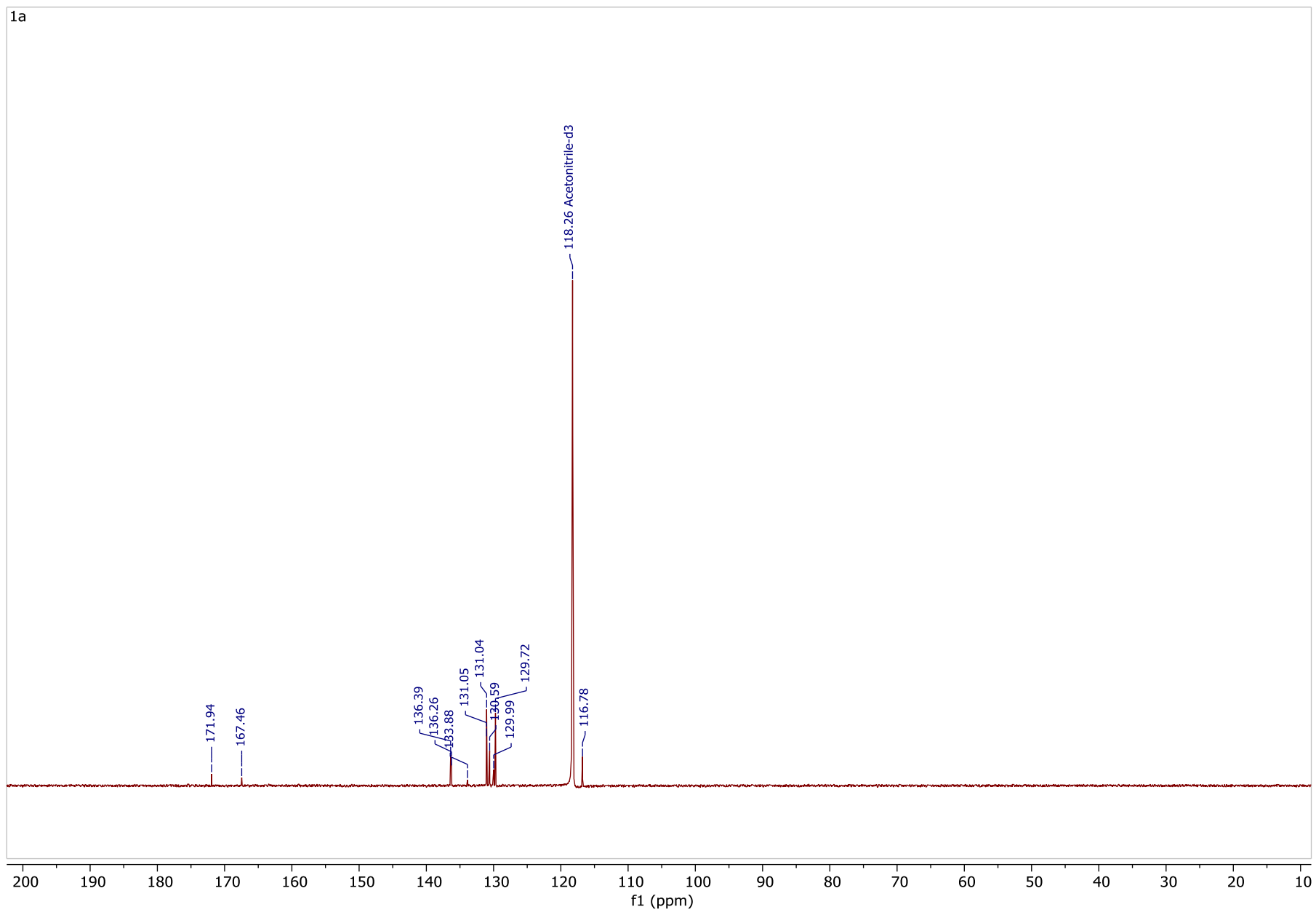

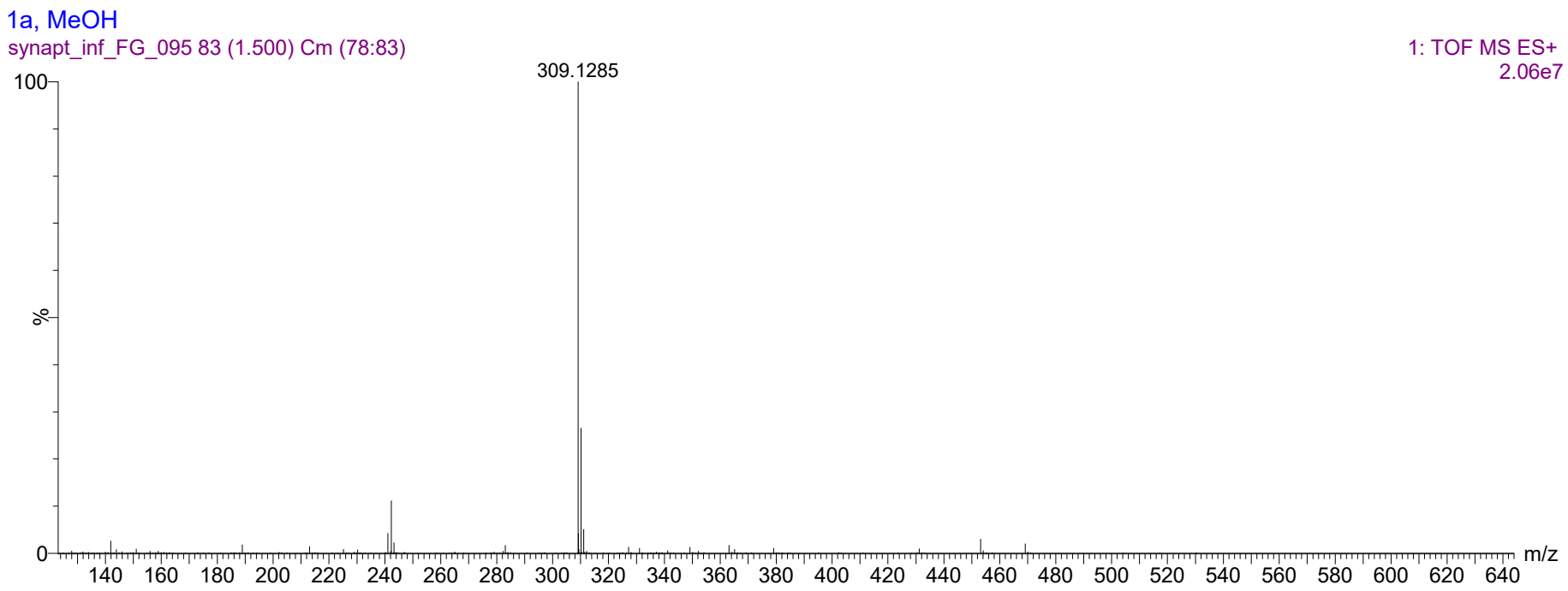

Figure S2. ^{1}H NMR, ^{13}C NMR (CD_3CN) and mass spectra of compound **1a**.

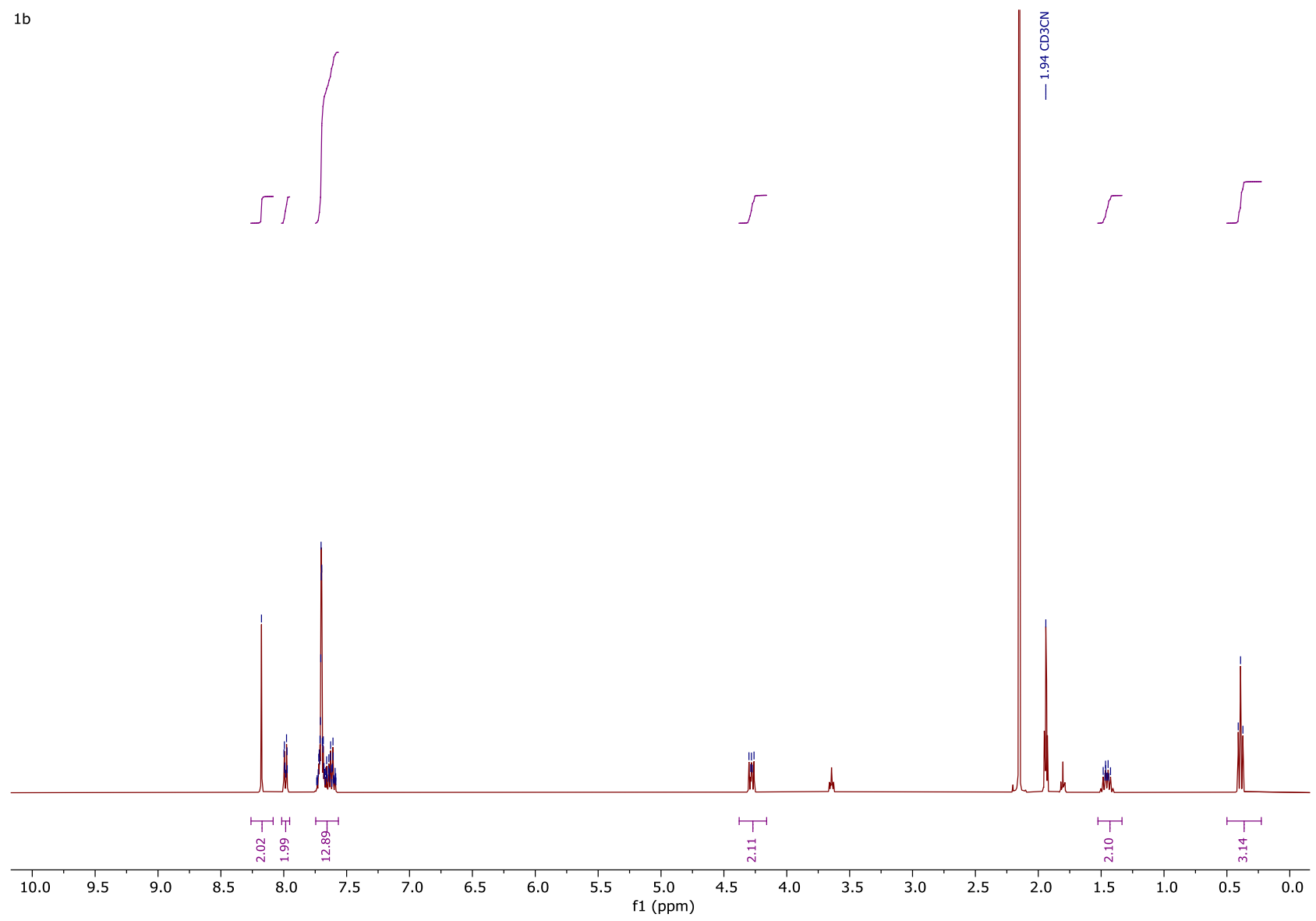

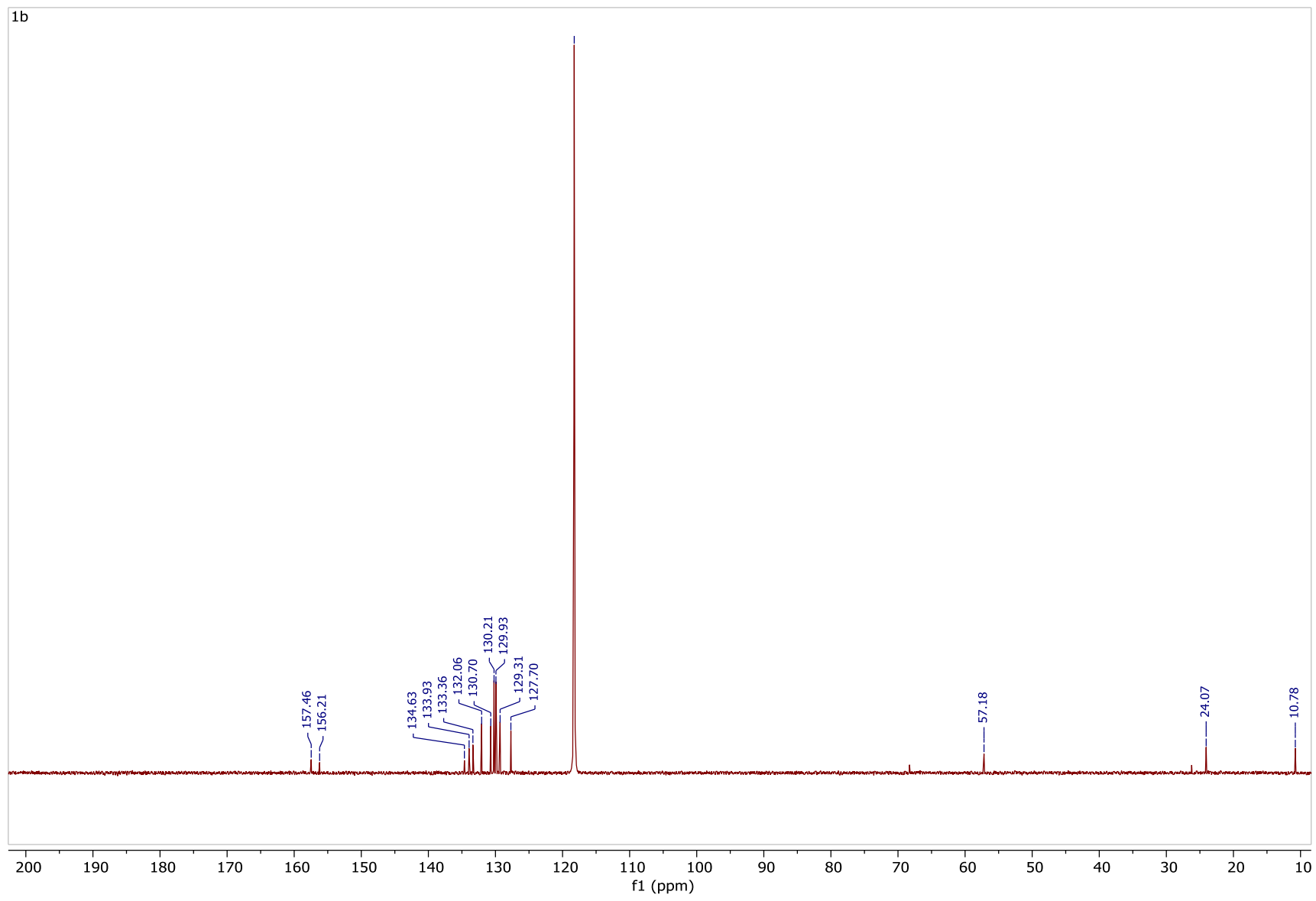

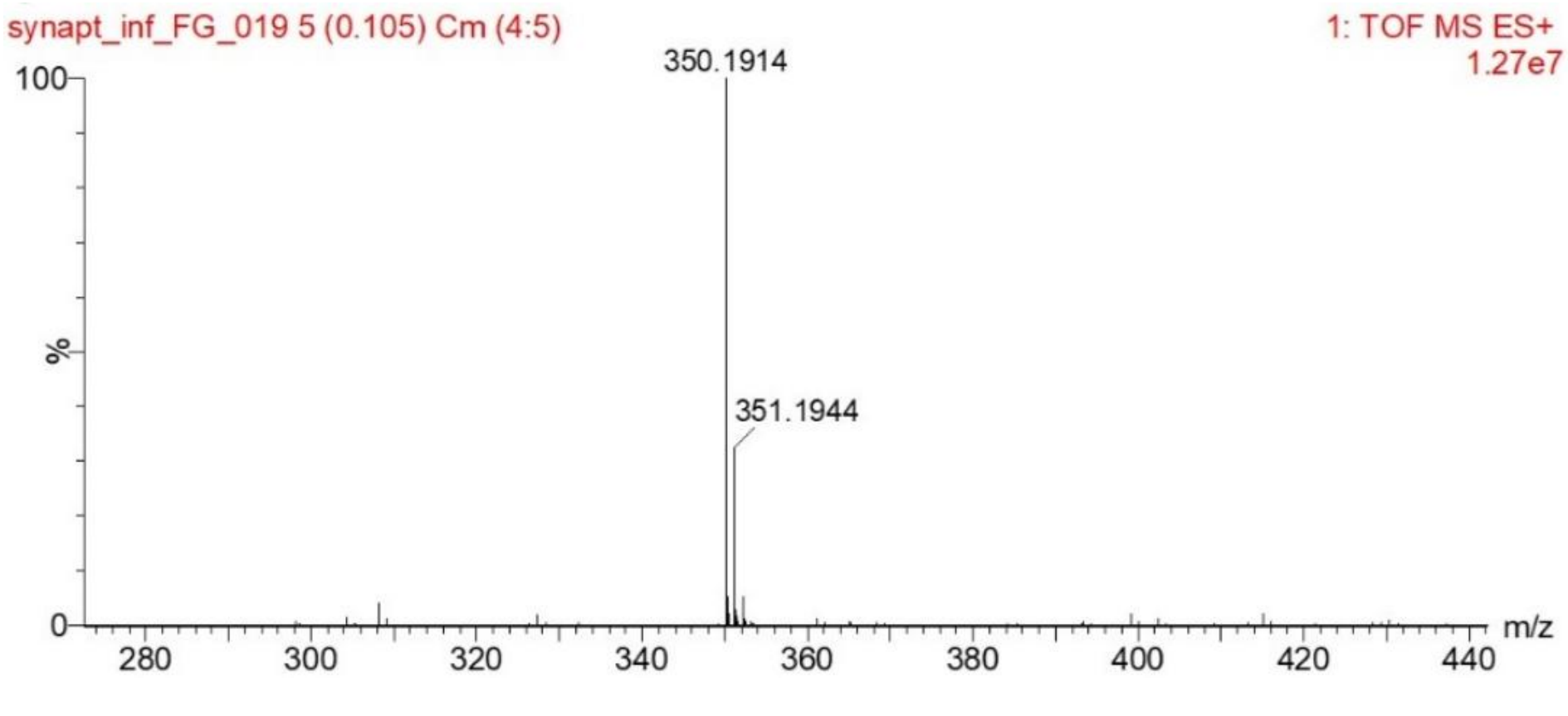

Figure S3. ^{1}H NMR, ^{13}C NMR (CD_3CN) and mass spectra of compound **1b**.

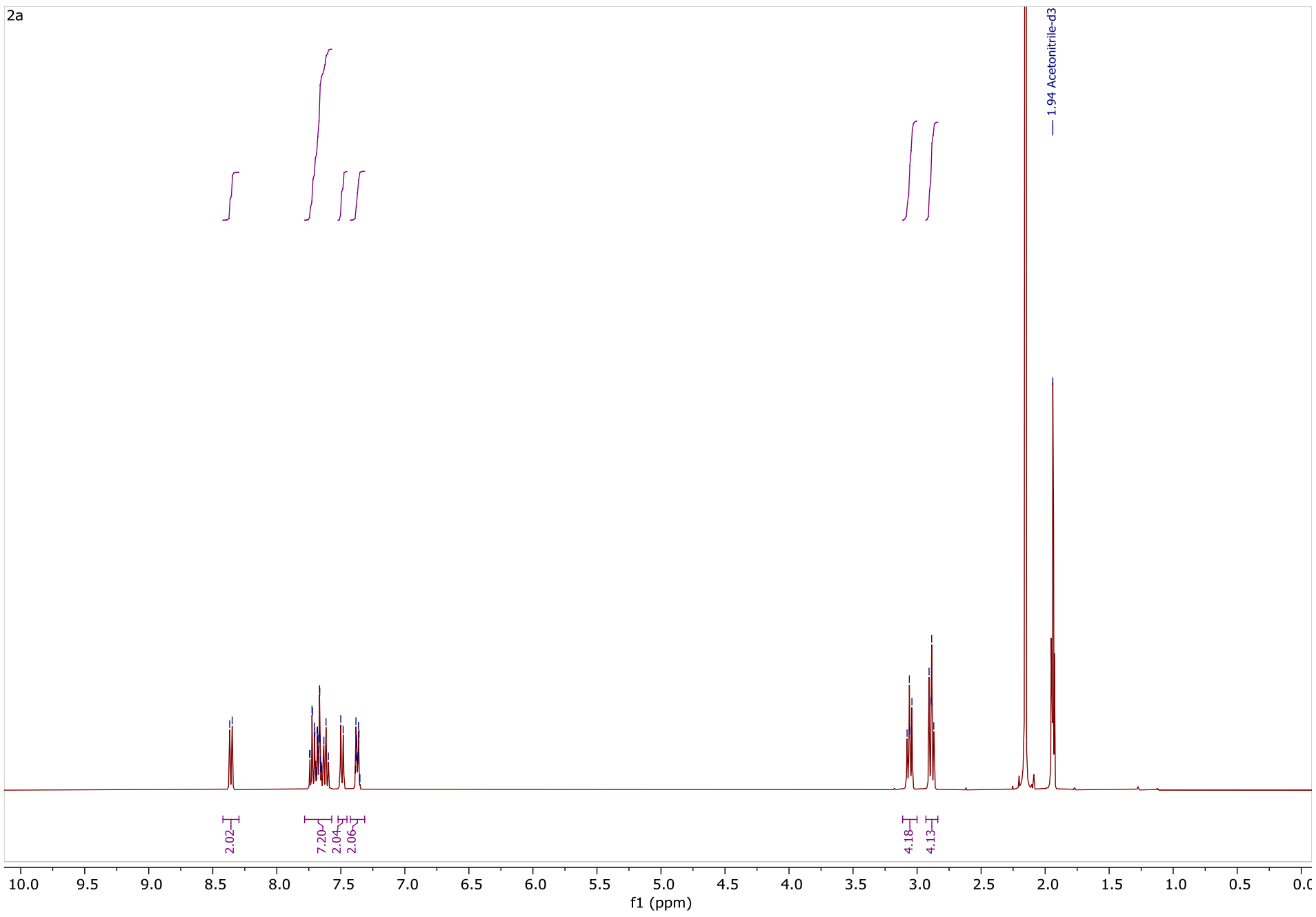

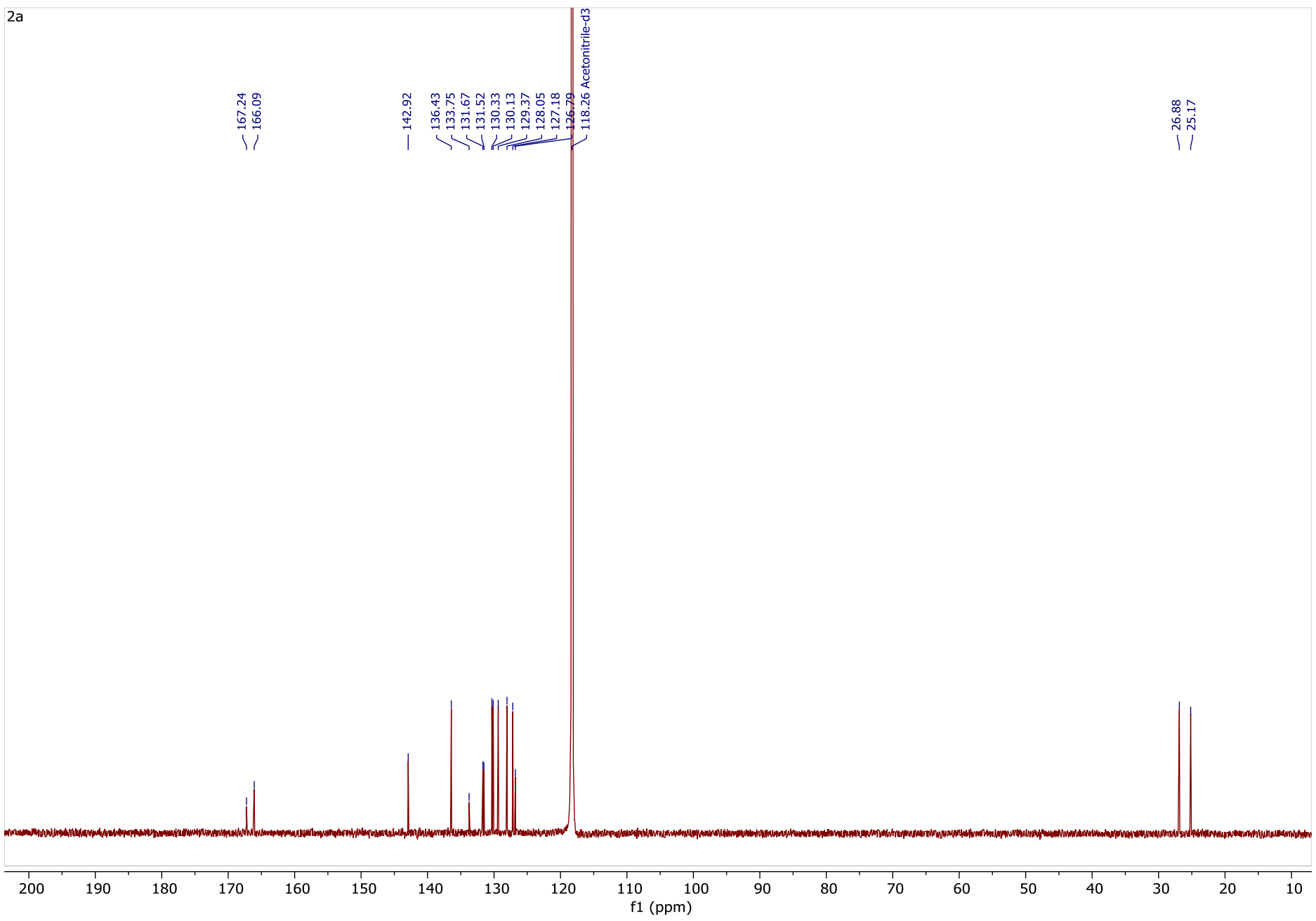

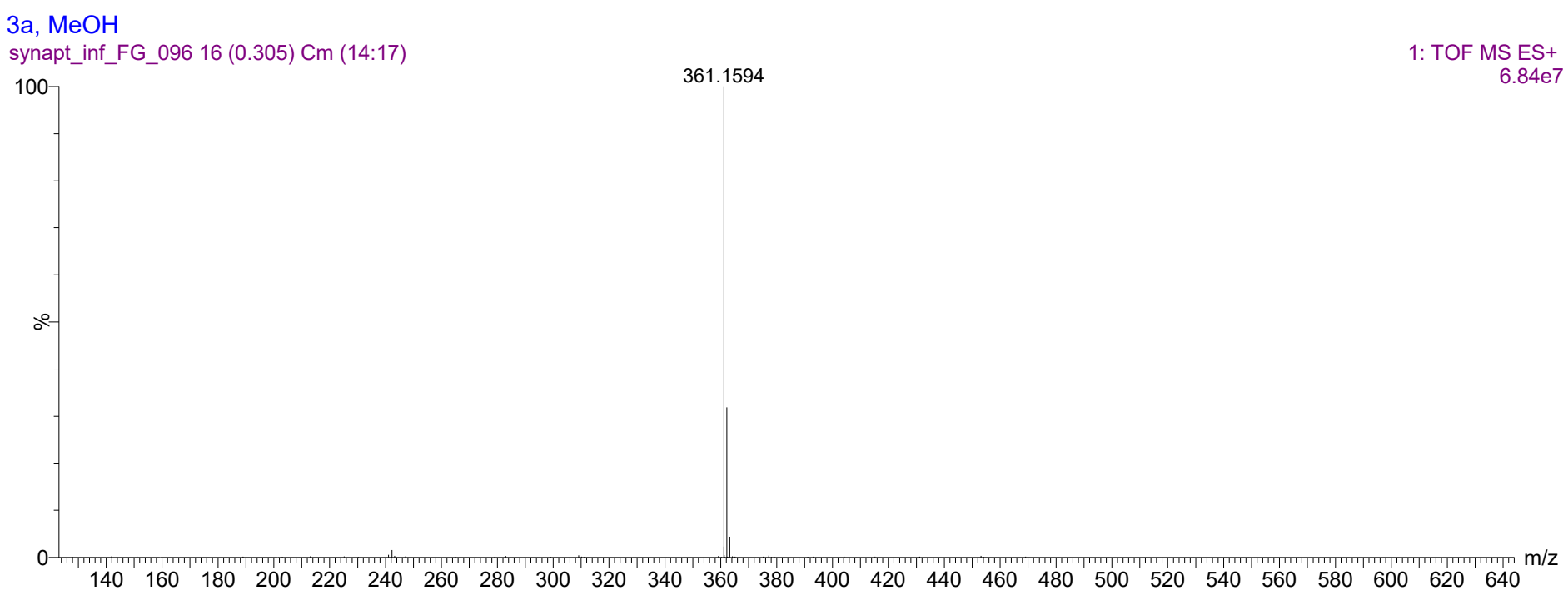

Figure S4. ^{1}H NMR, ^{13}C NMR (CD_3CN) and mass spectra of compound **2a**.

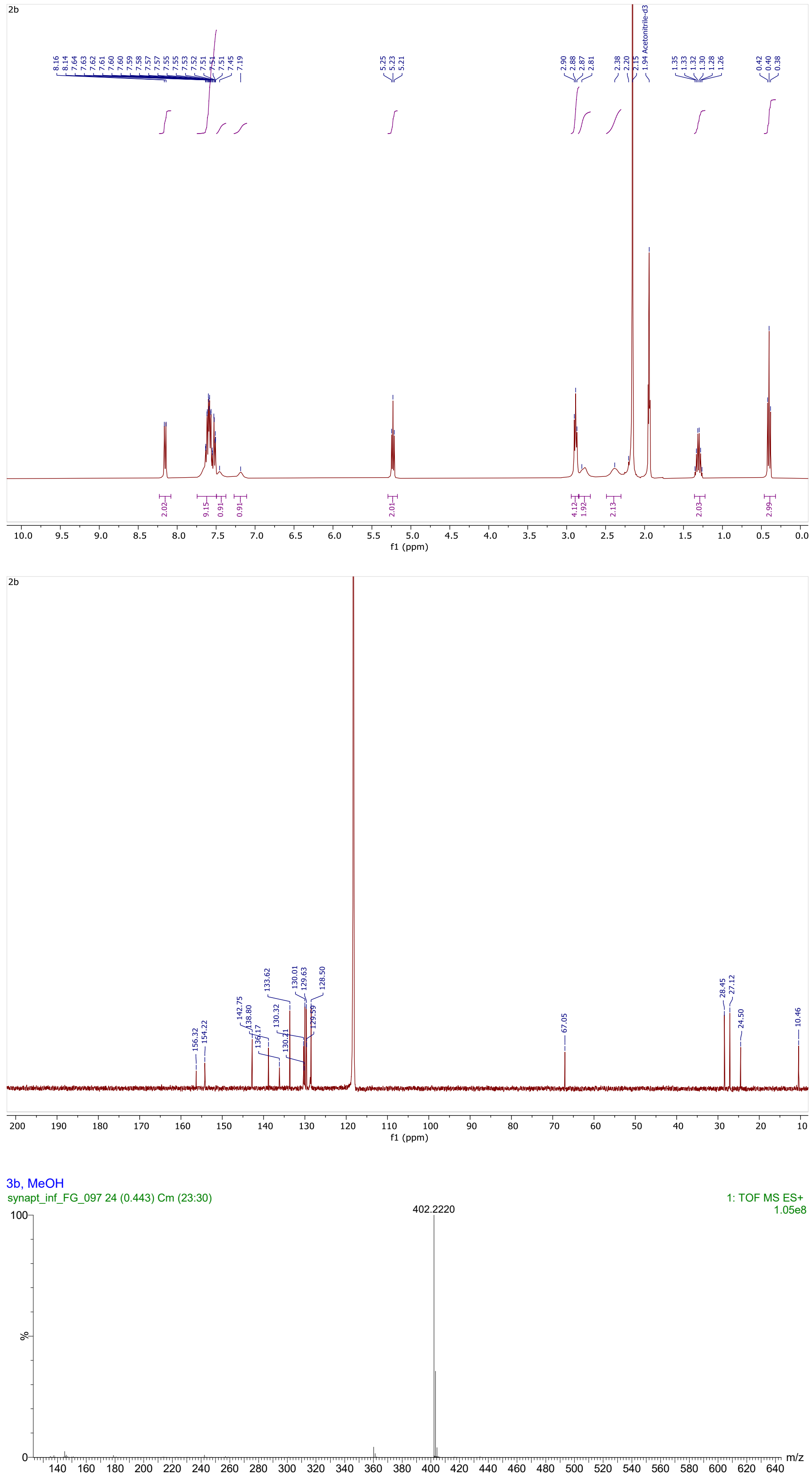

Figure S5. ^{1}H NMR, ^{13}C NMR (CD_3CN) and mass spectra of compound **2b**.

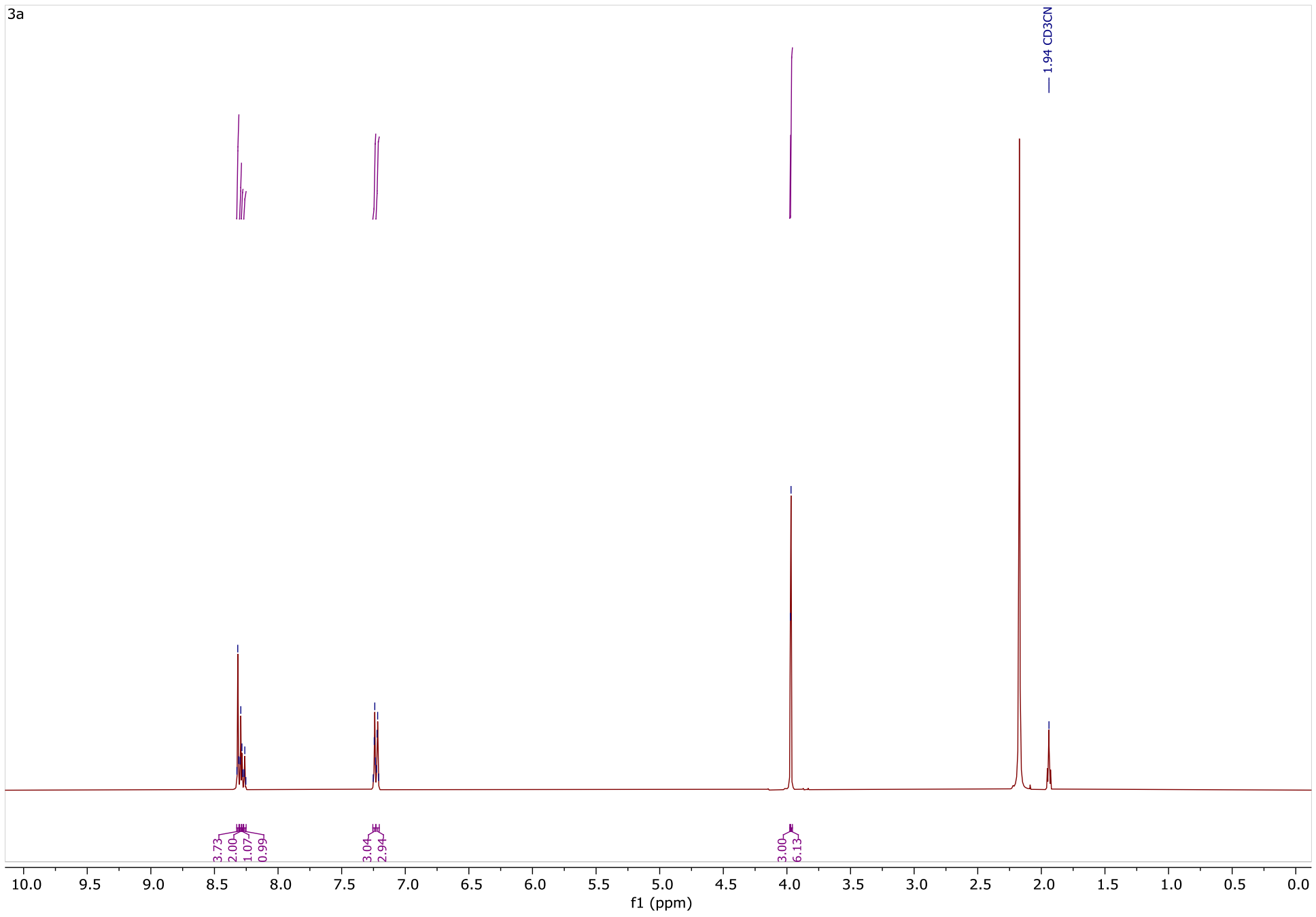

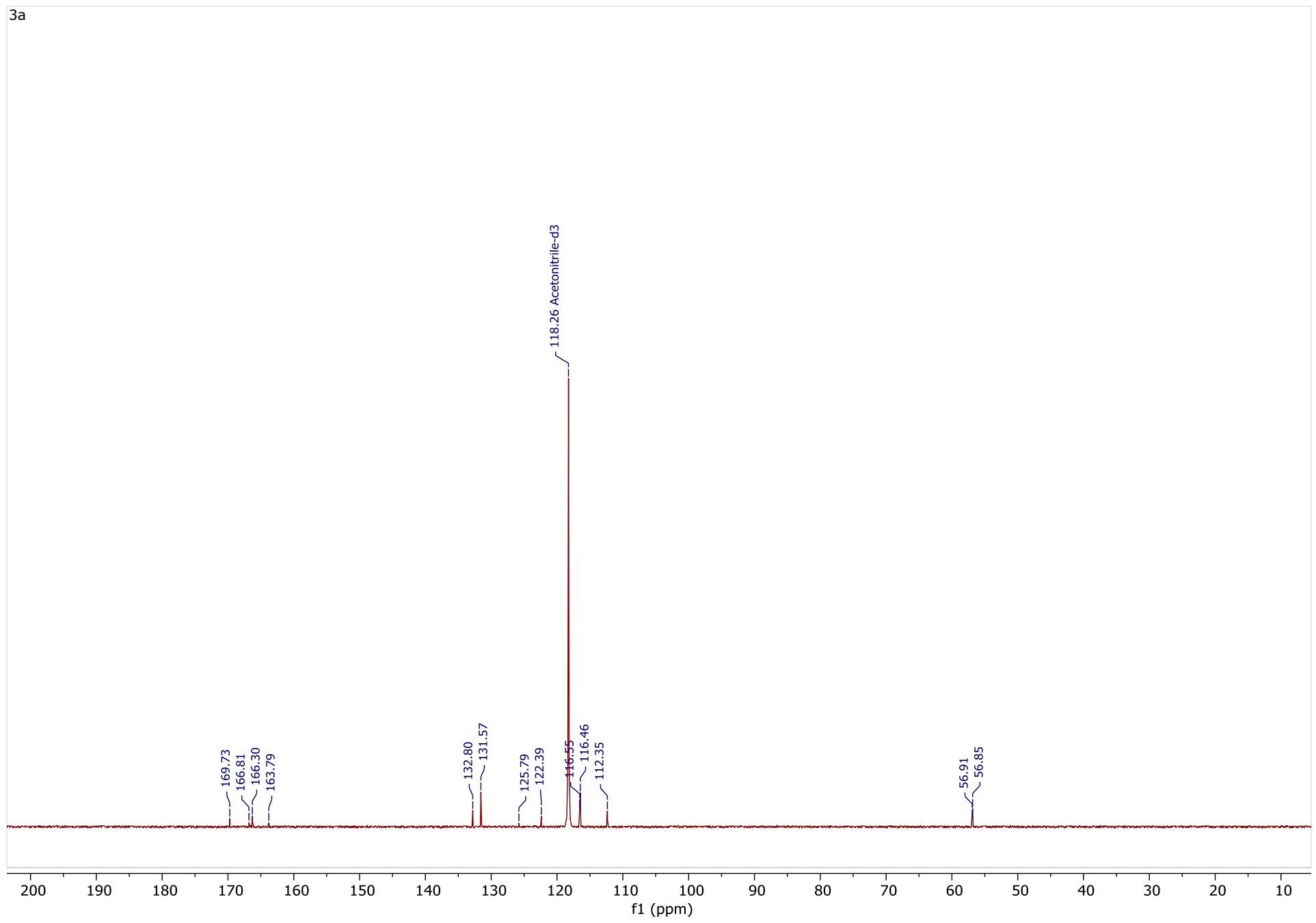

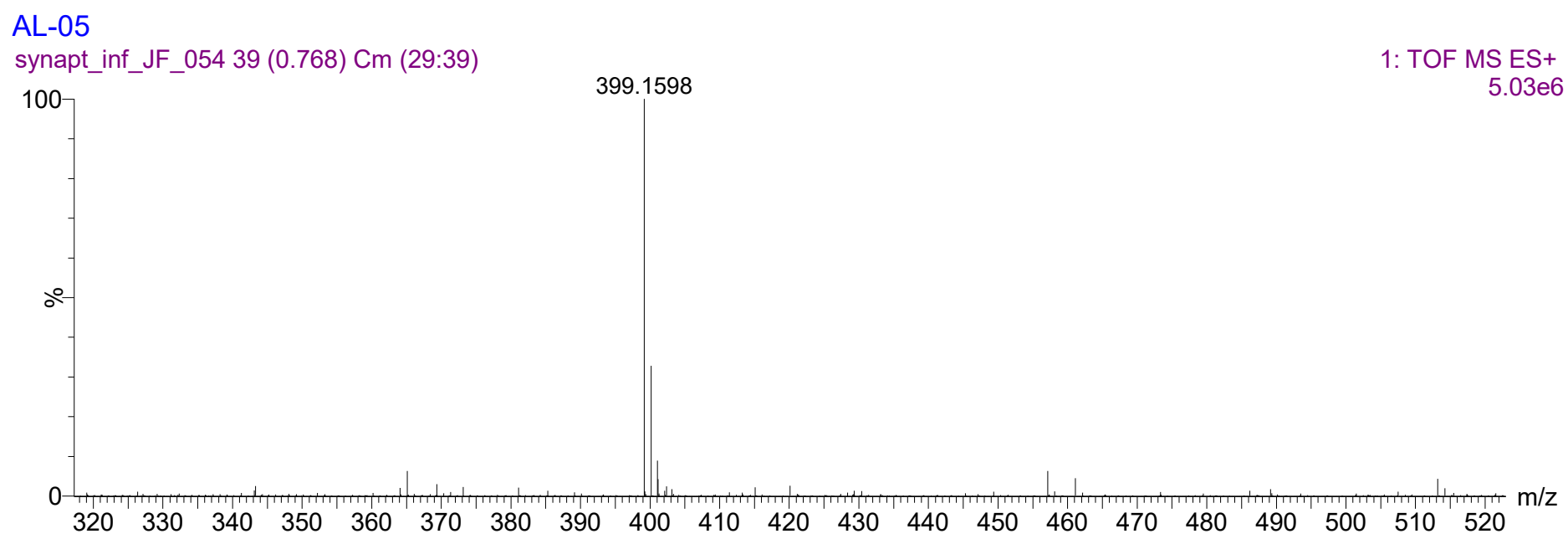

Figure S6. ^{1}H NMR, ^{13}C NMR (CD_3CN) and mass spectra of compound **3a**.

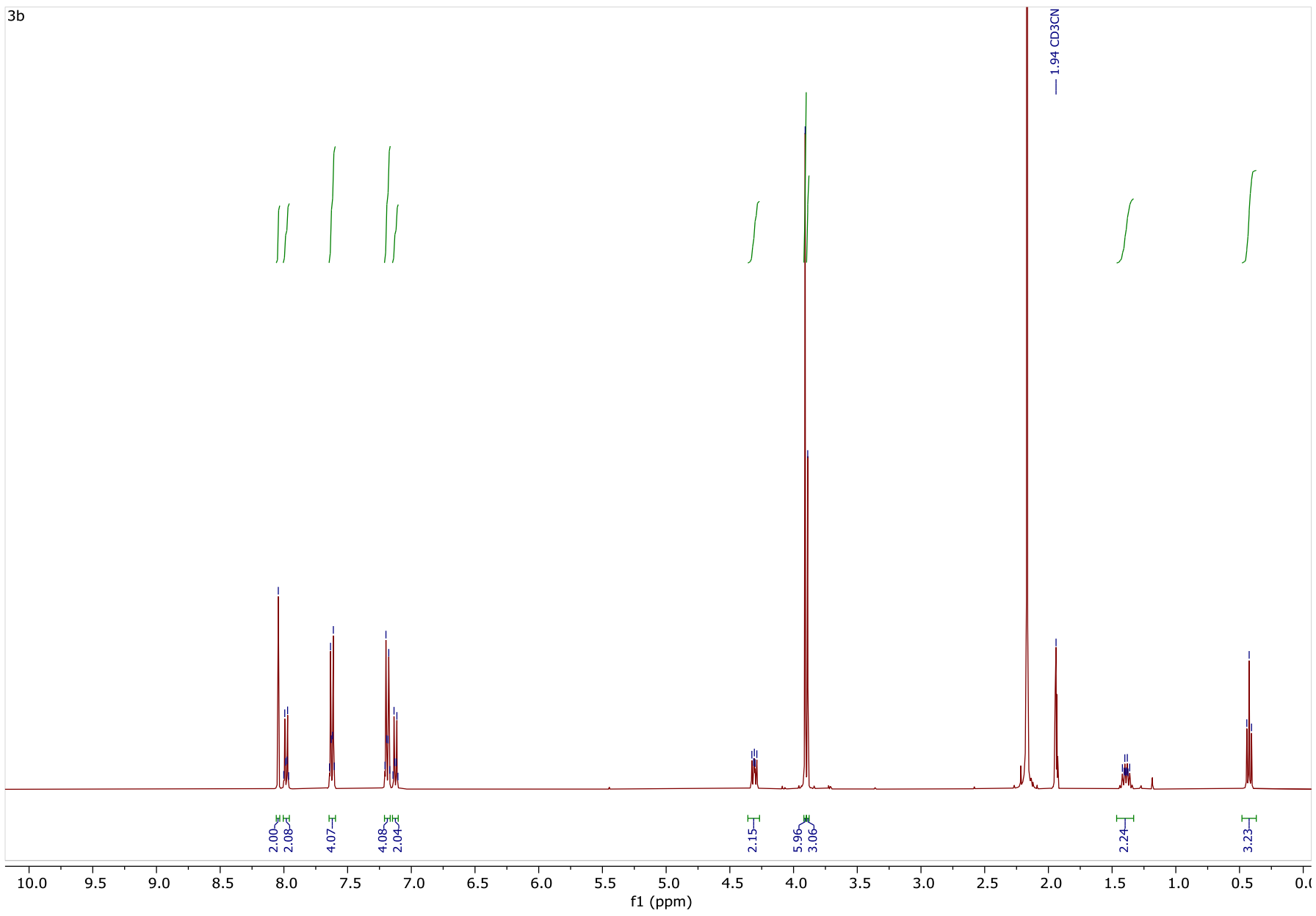

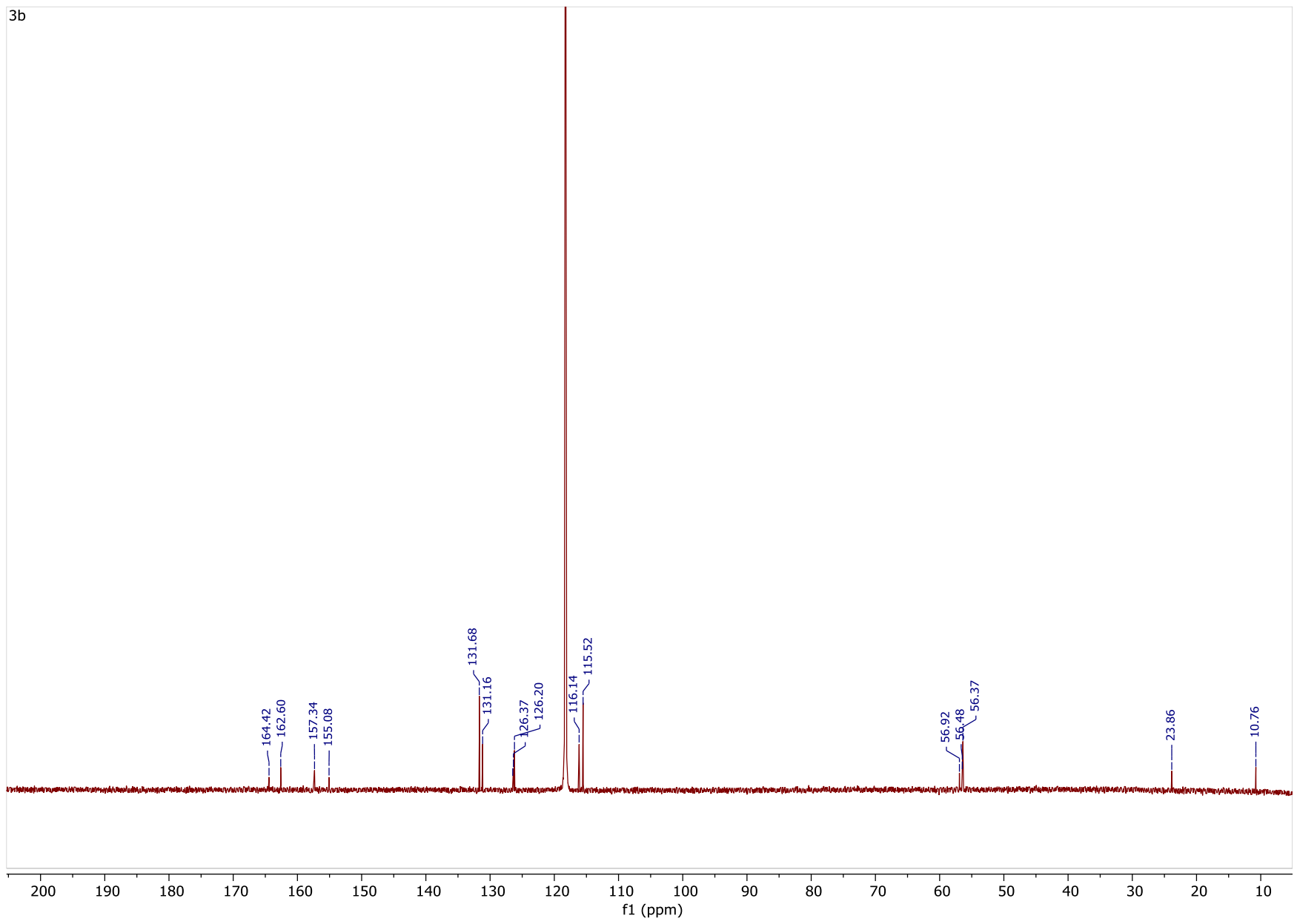

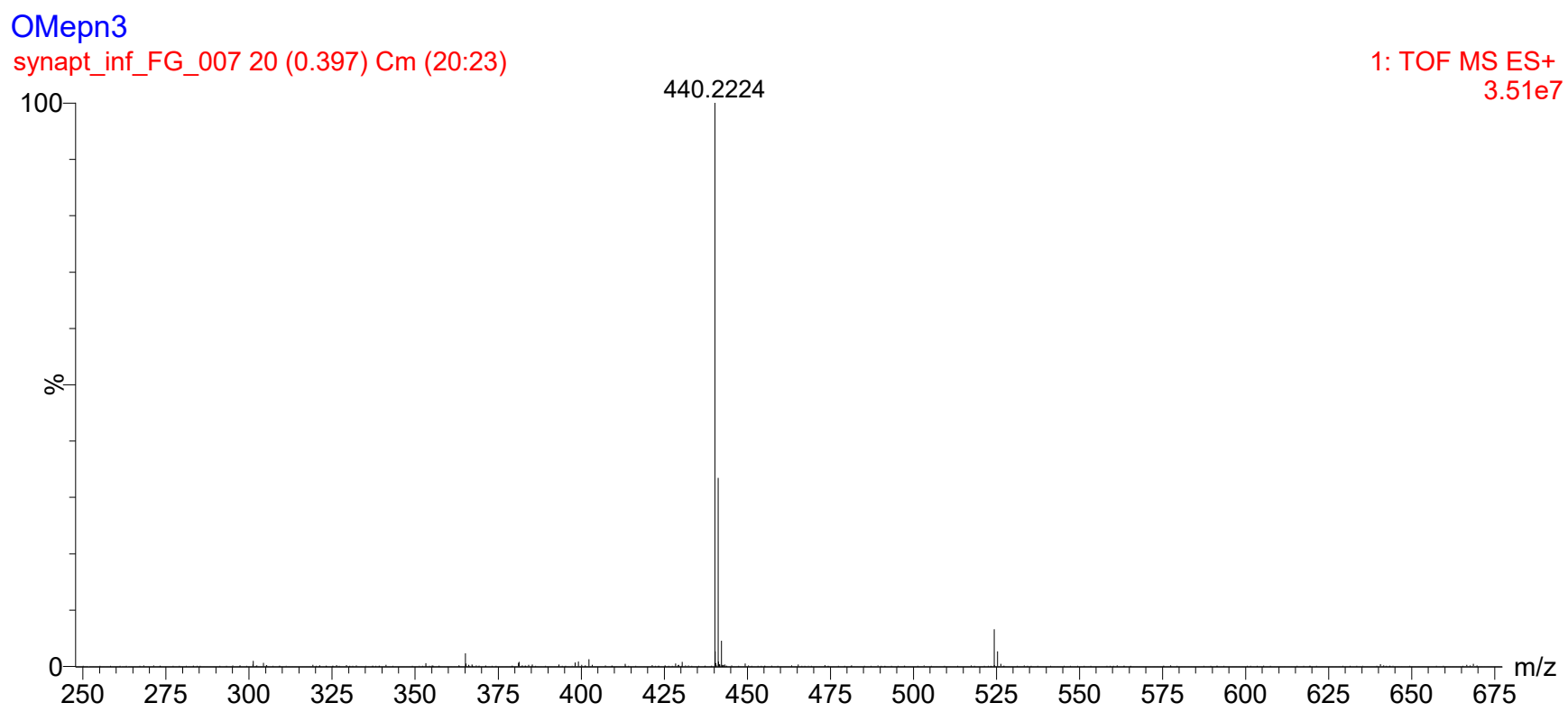

Figure S7. ^{1}H NMR, ^{13}C NMR (CD_3CN) and mass spectra of compound **3b**.

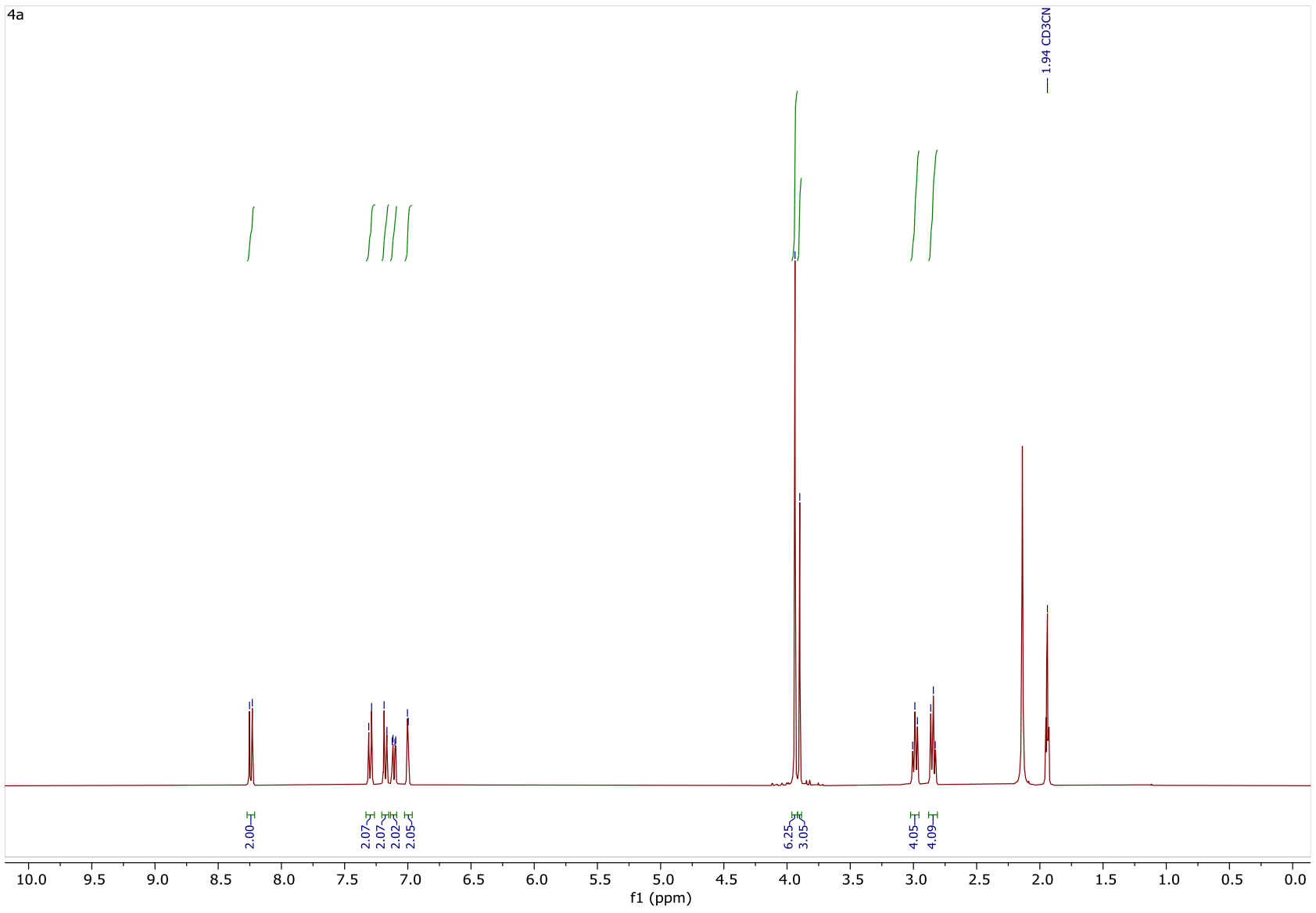

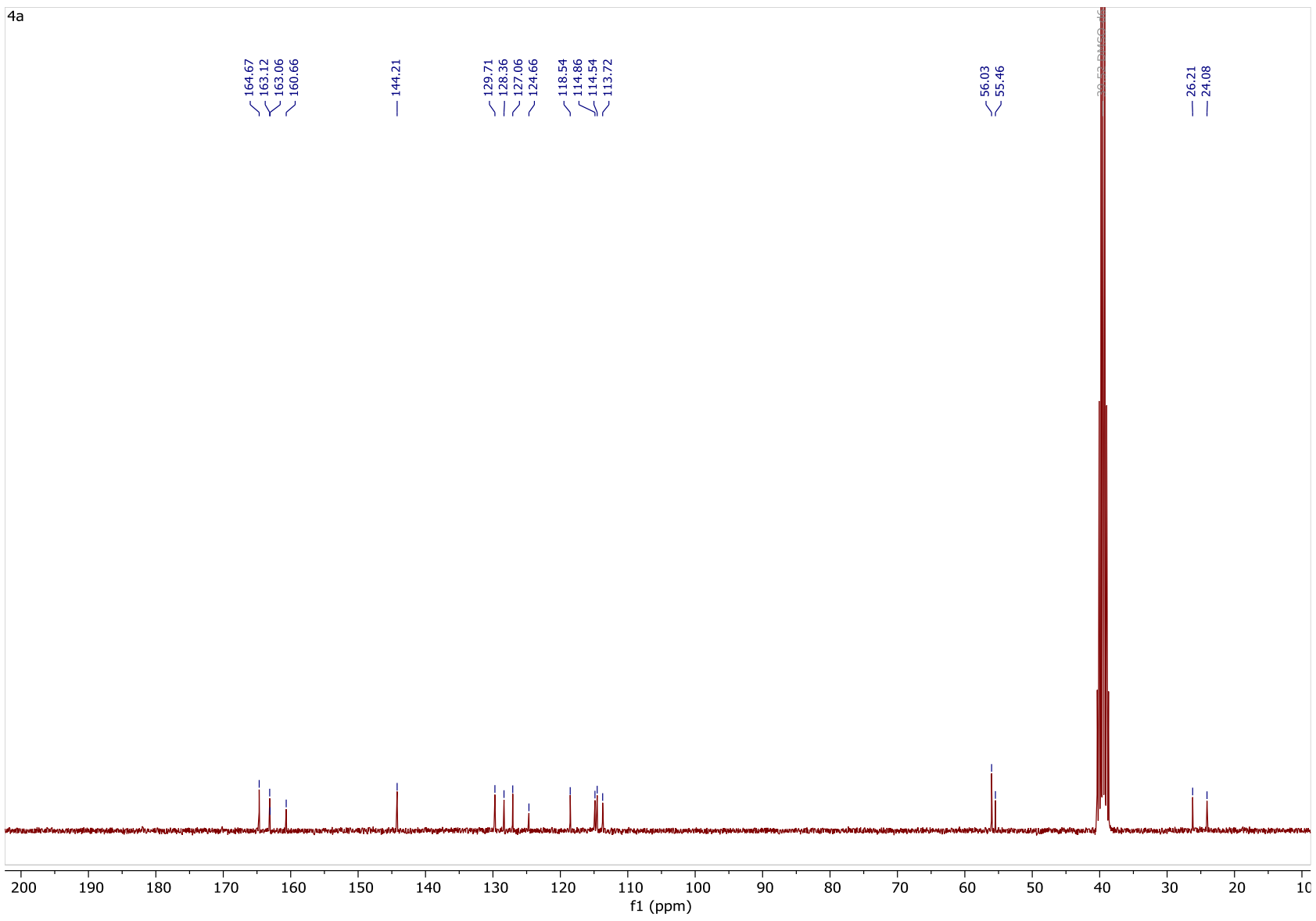

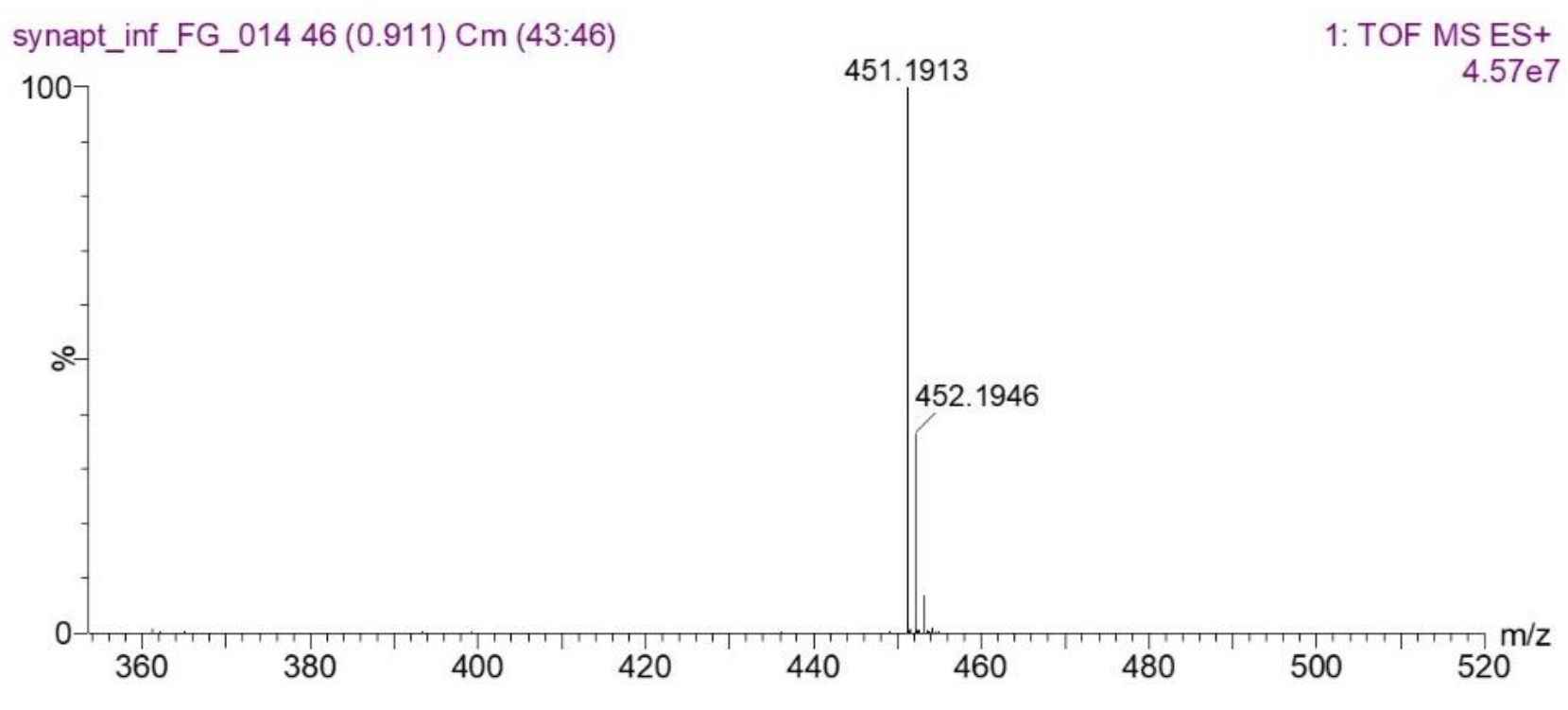

Figure S8. ^{1}H NMR, ^{13}C NMR (CD_3CN) and mass spectra of compound **4a**.

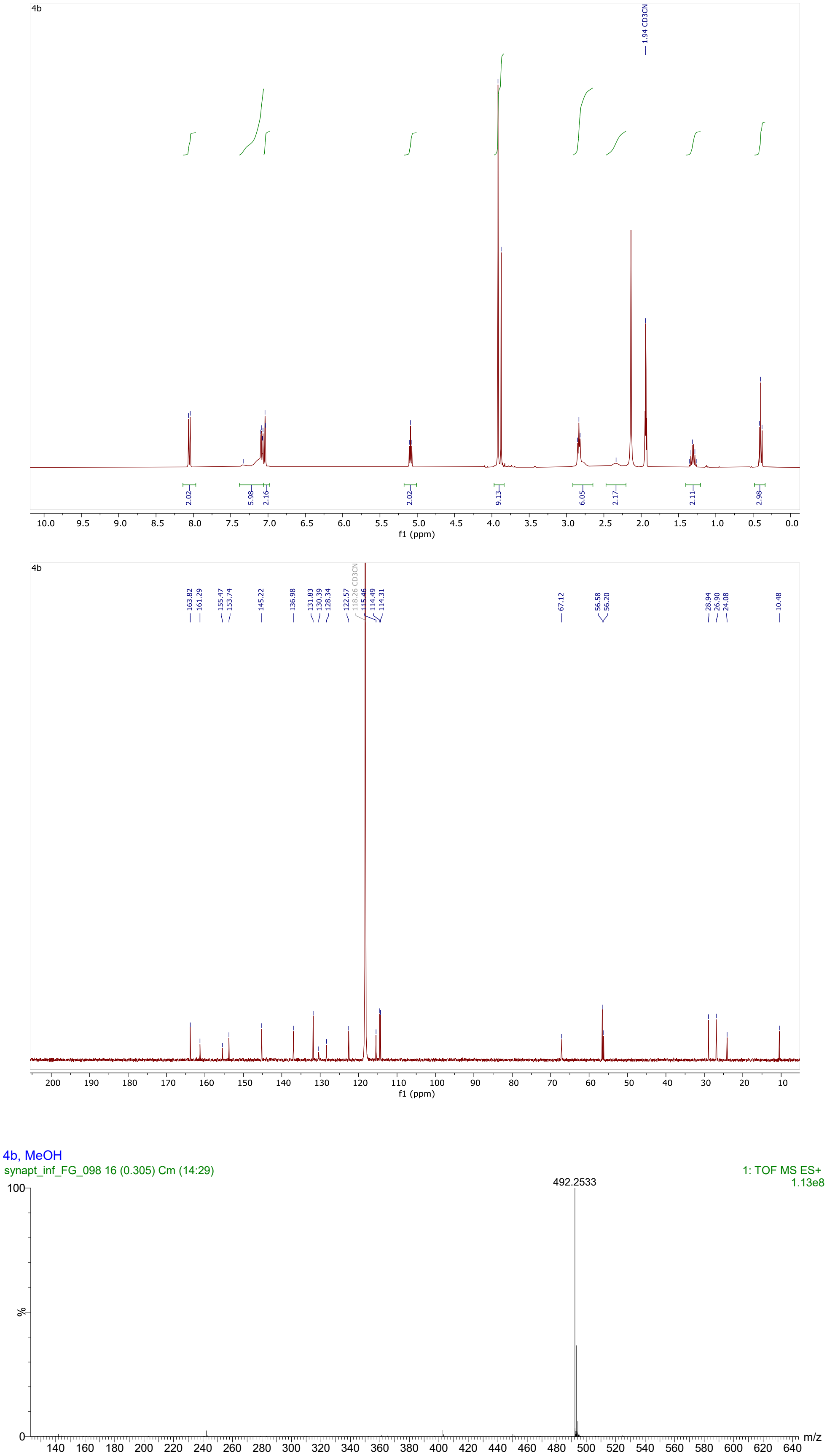

iFigure S9. ^{1}H NMR, ^{13}C NMR (CD_3CN) and mass spectra of compound **4b**.

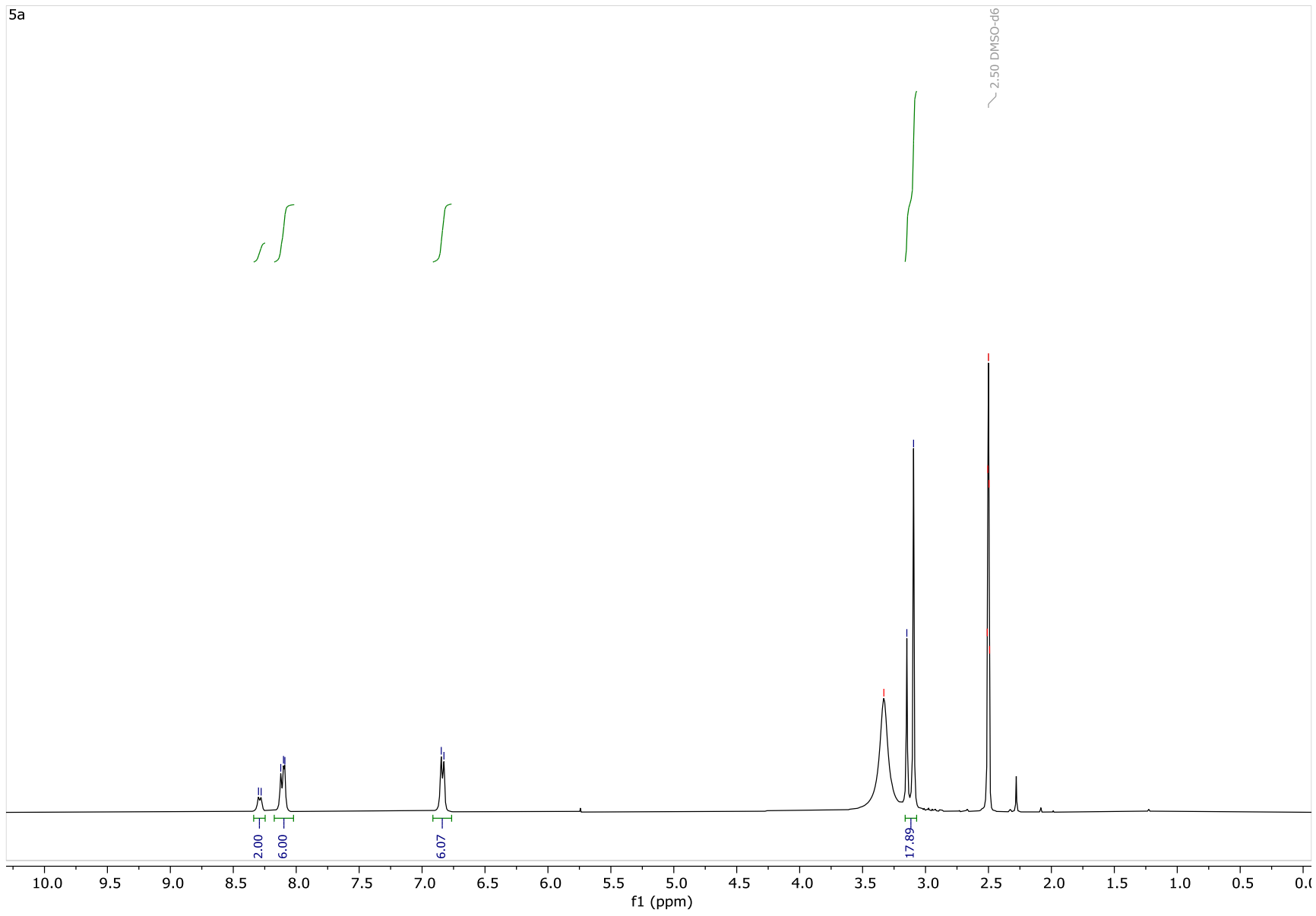

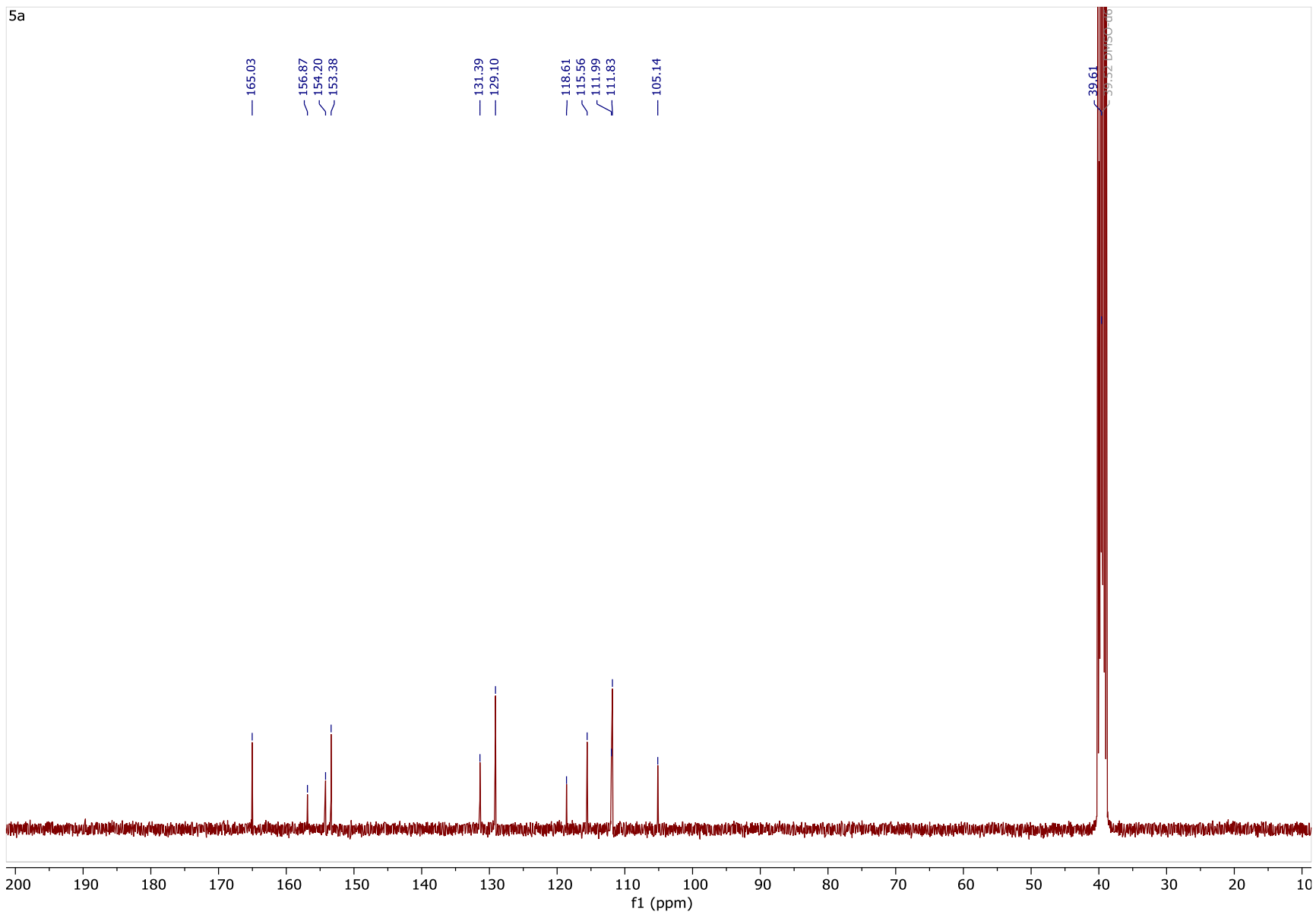

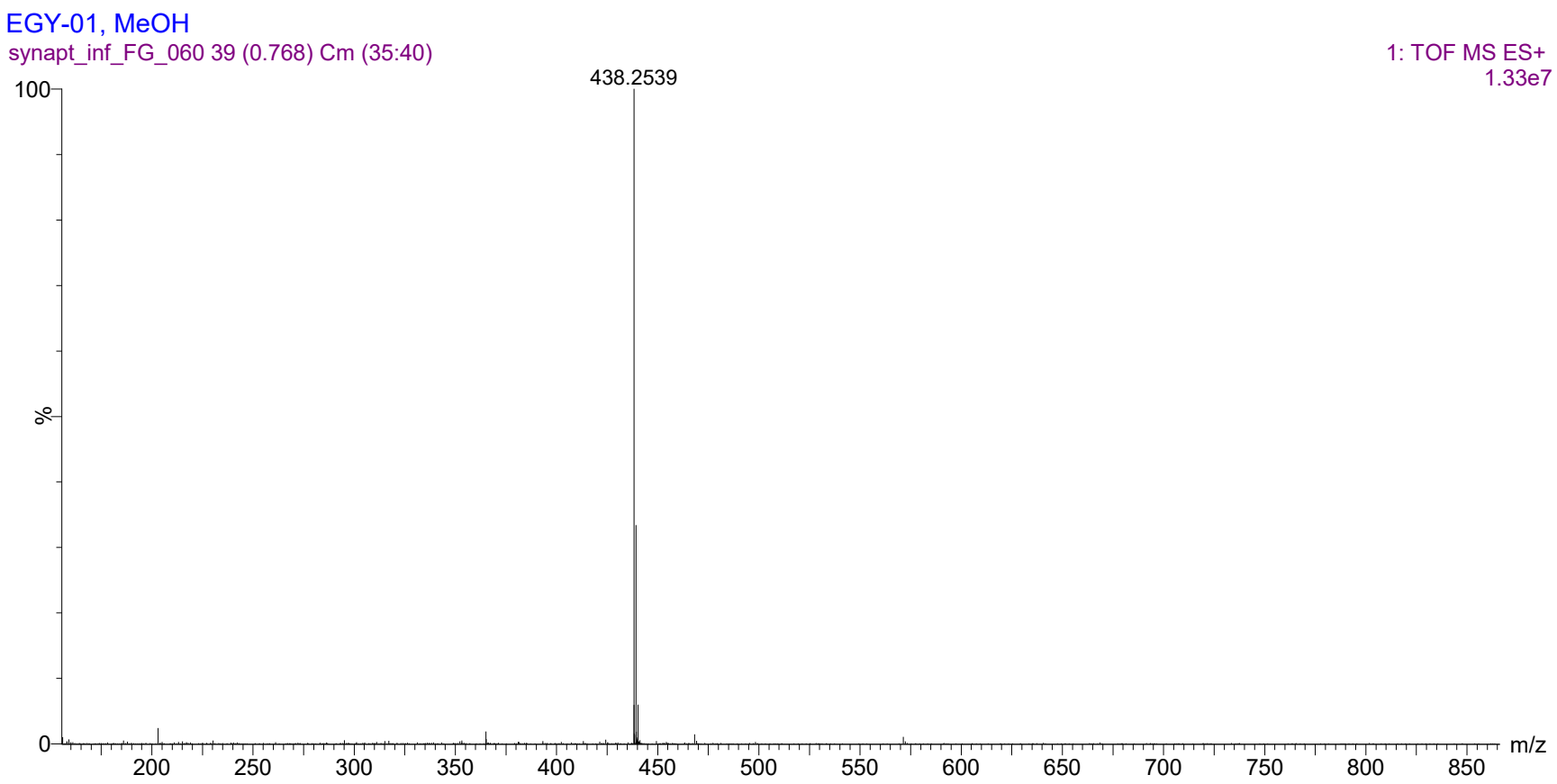

Figure S10. ^{1}H NMR, ^{13}C NMR (DMSO-d$_6$) and mass spectra of compound **5a**.

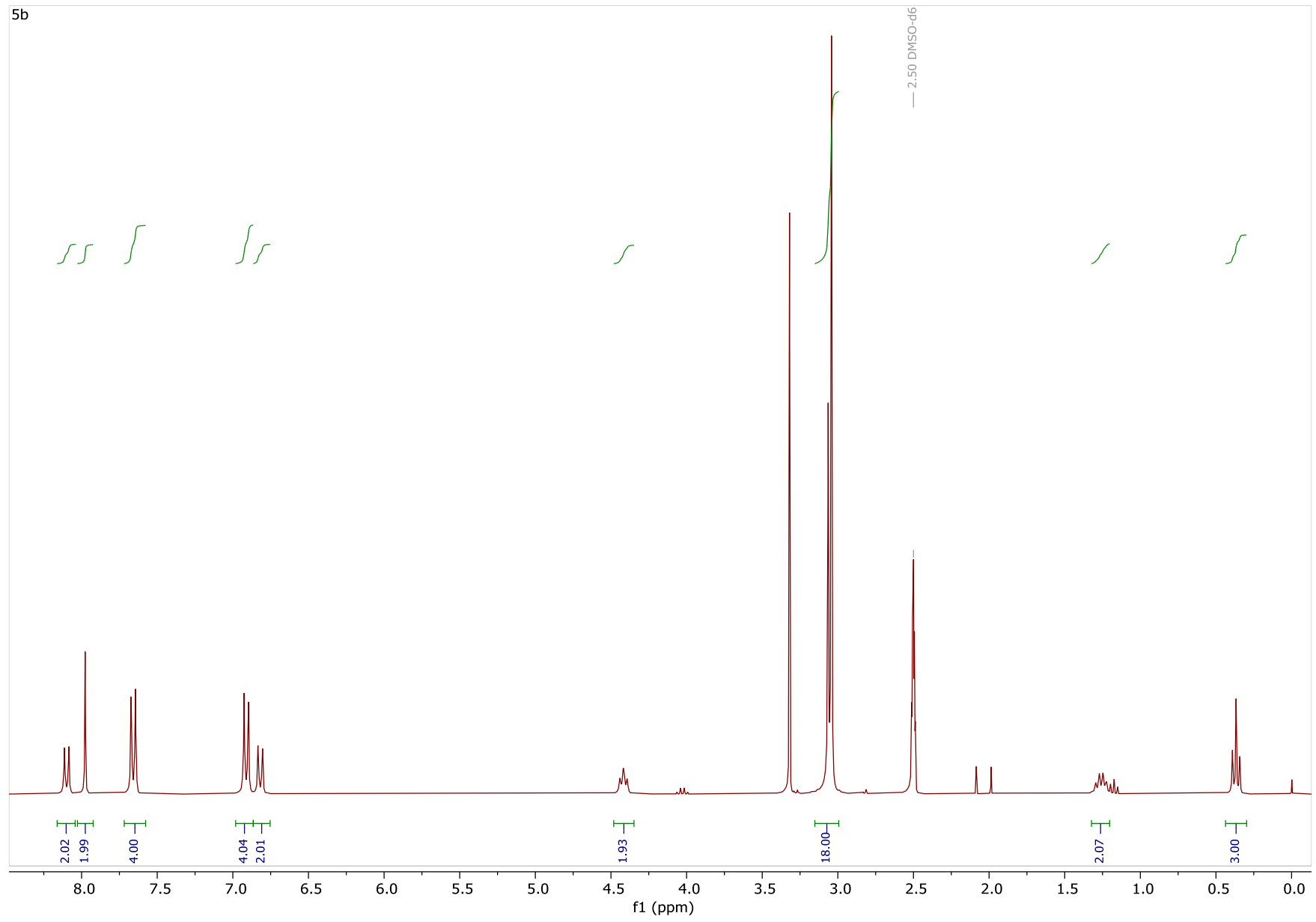

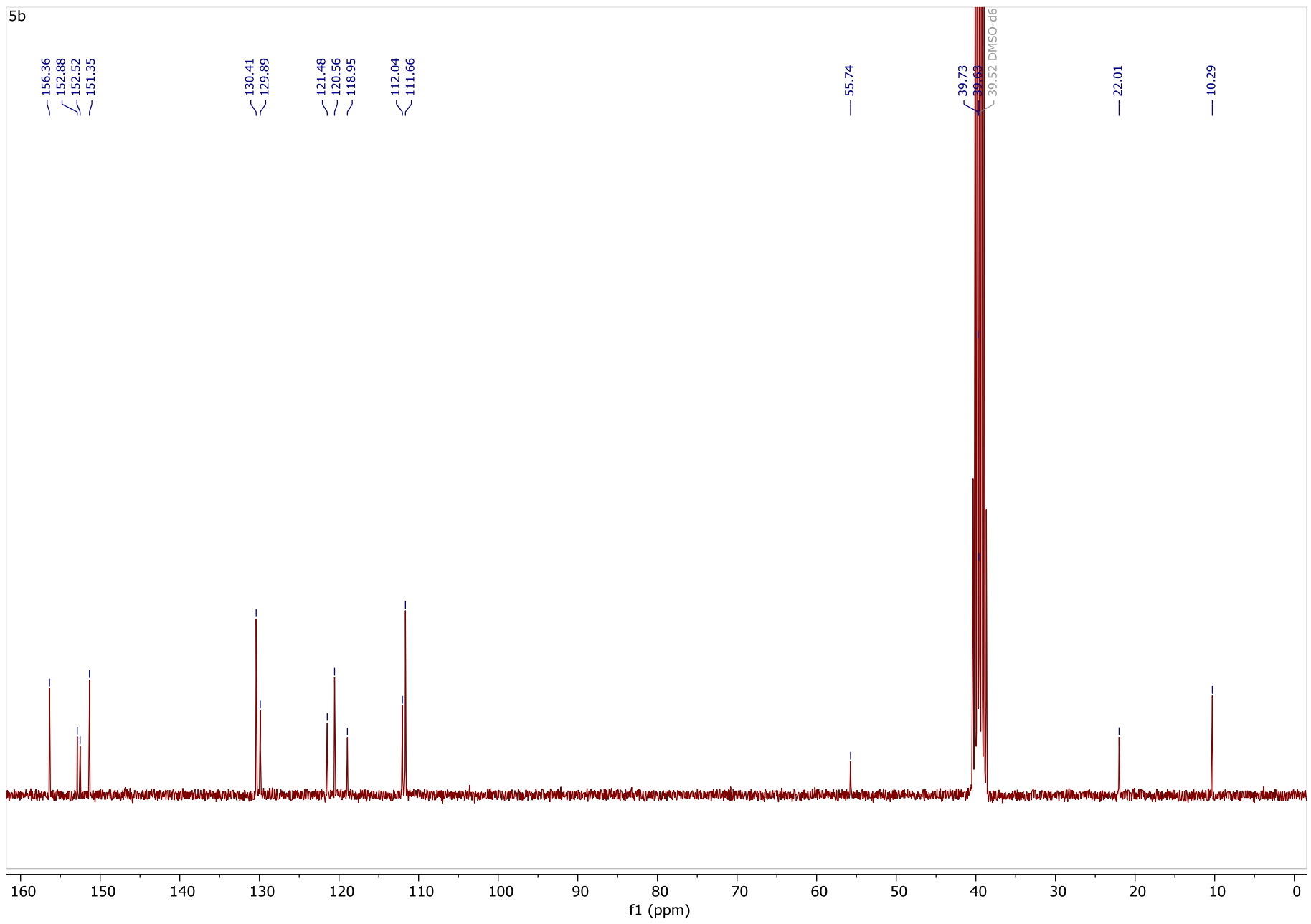

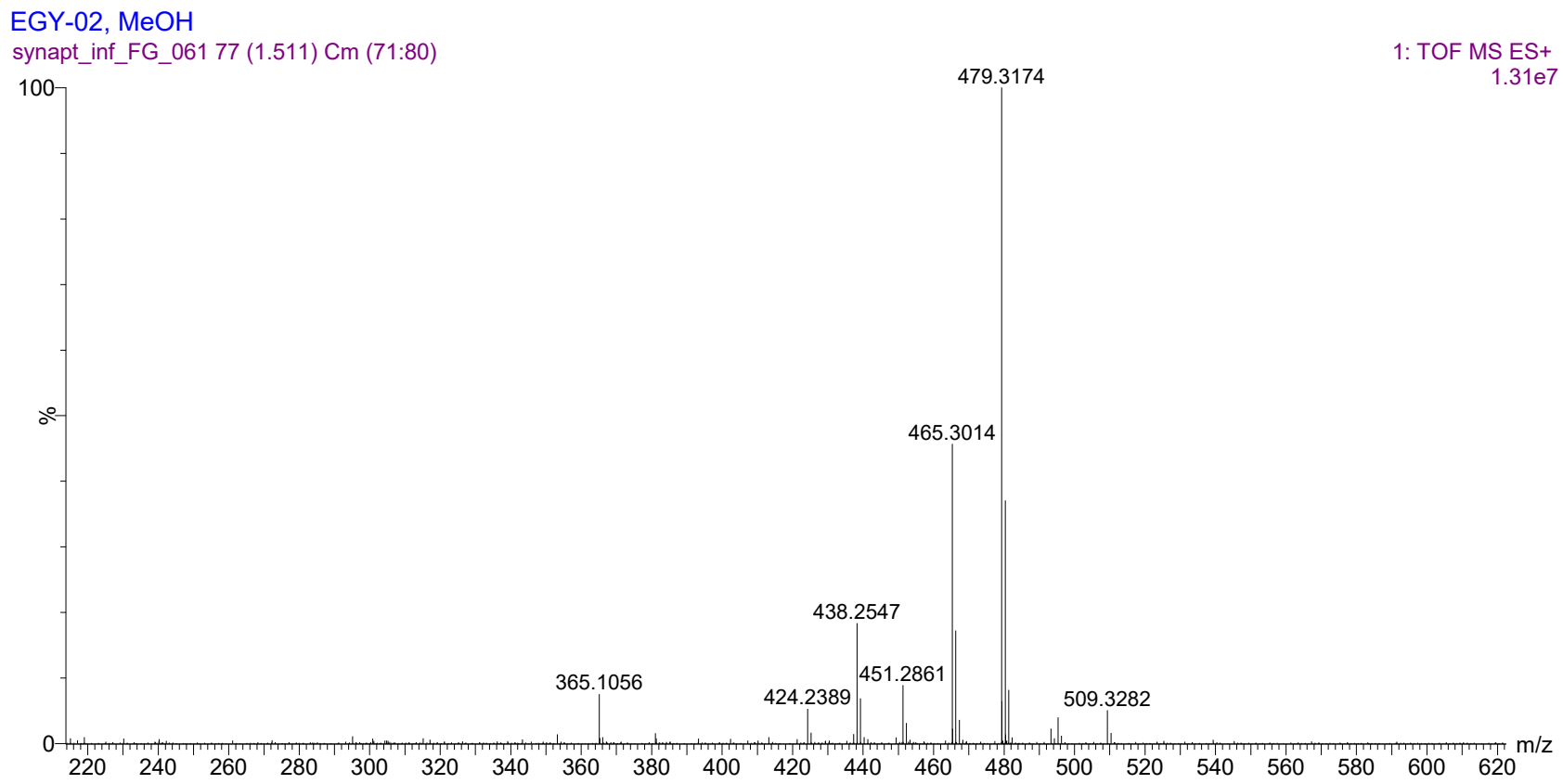

Figure S11. ^{1}H NMR, ^{13}C NMR (DMSO-d$_6$) and mass spectra of compound **5b**.

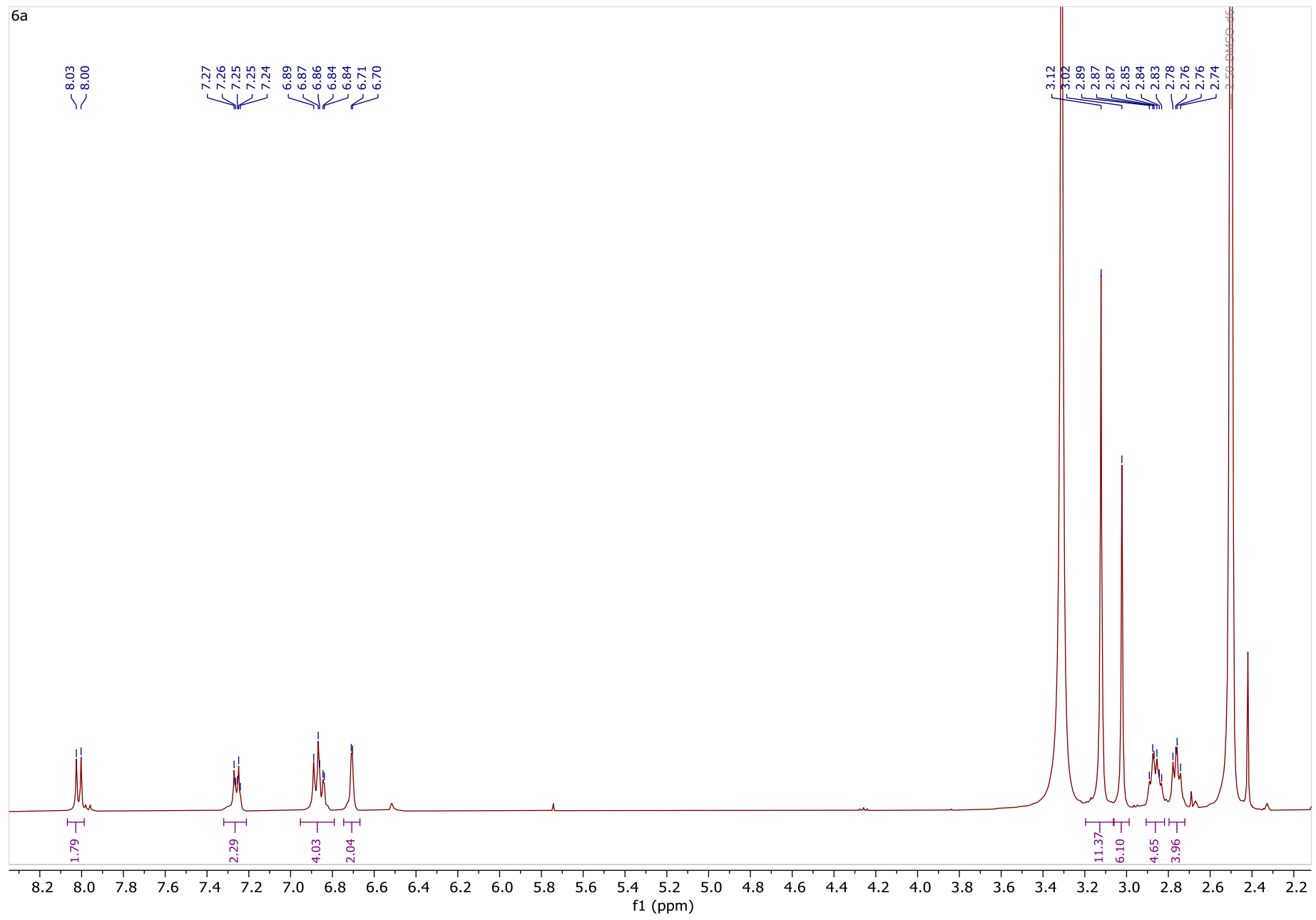

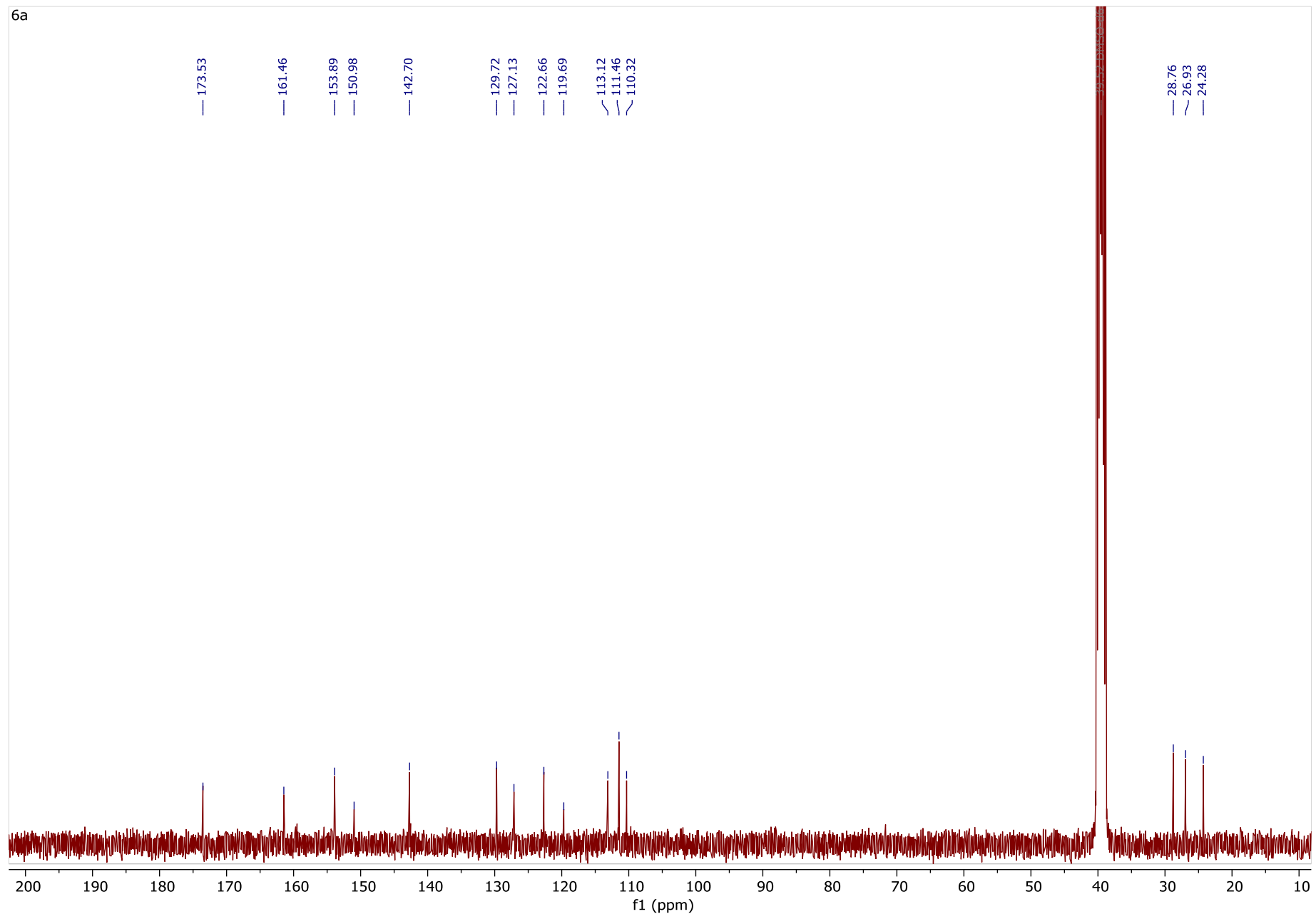

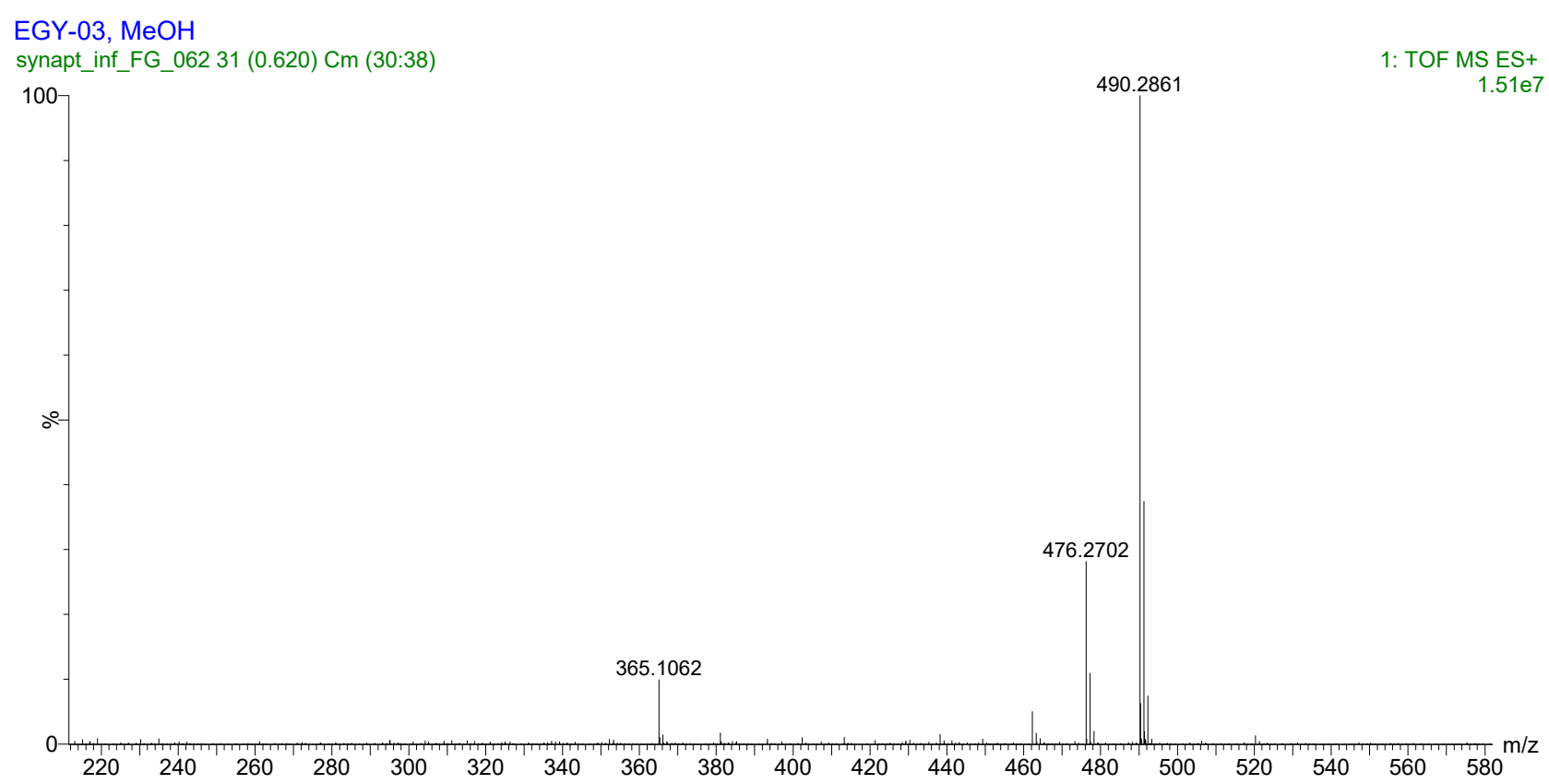

Figure S12. ^{1}H NMR, ^{13}C NMR (DMSO-d$_6$) and mass spectra of compound **6a**.

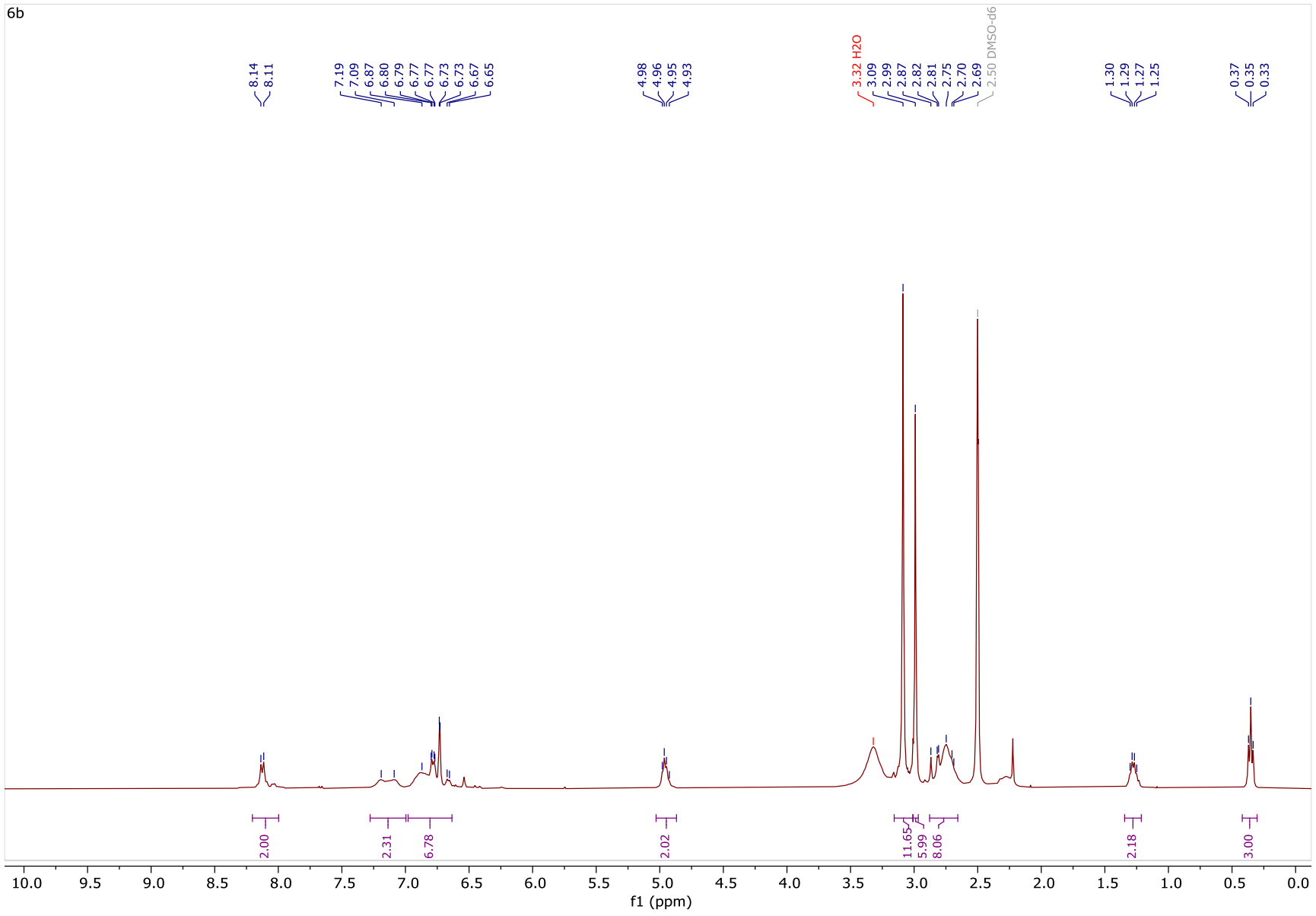

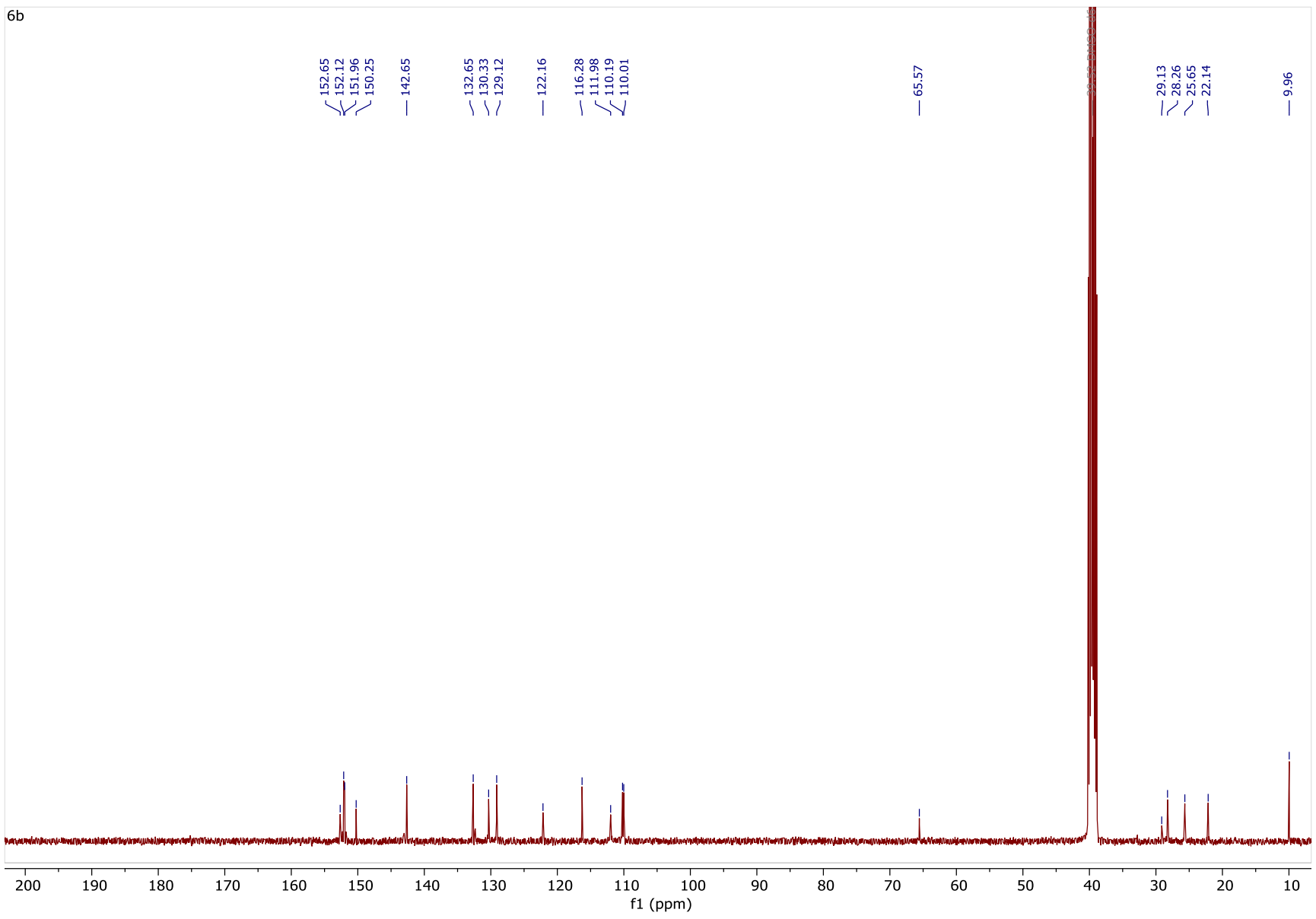

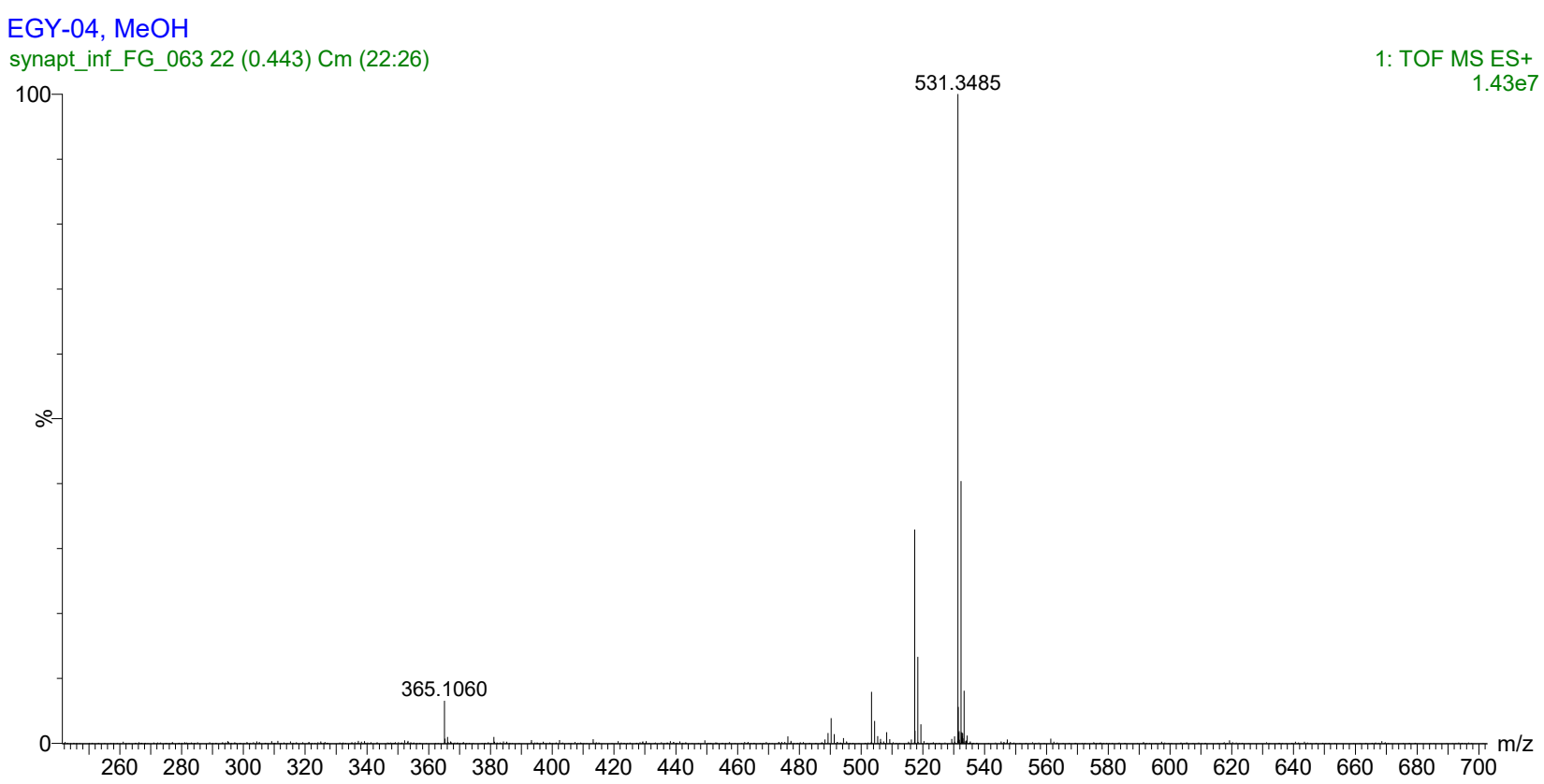

Figure S13. ^{1}H NMR, ^{13}C NMR (DMSO-d_6) and mass spectra of compound **6b**.

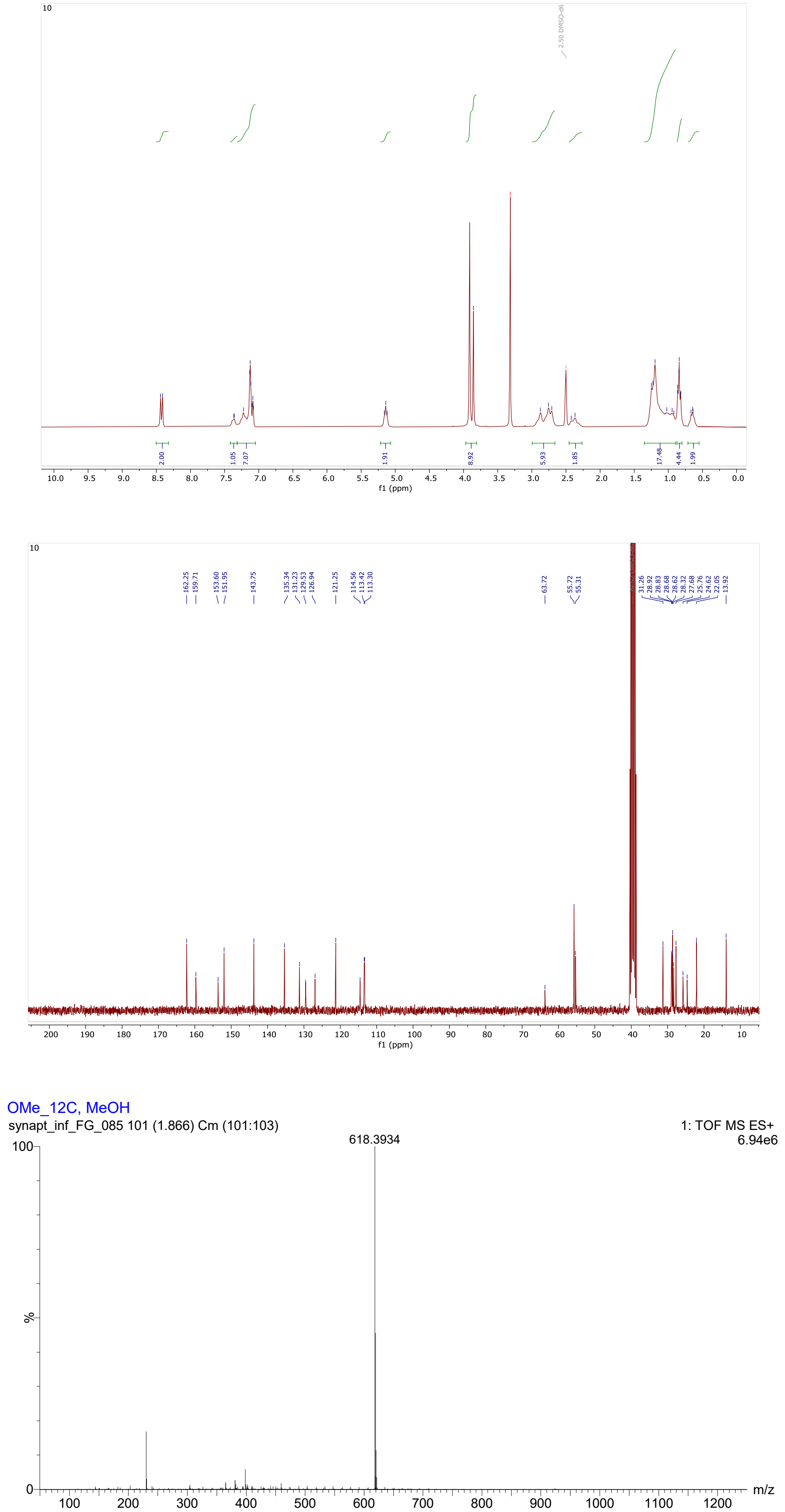

Figure S14. ^{1}H NMR, ^{13}C NMR (DMSO-d$_6$) and mass spectra of compound **7**.

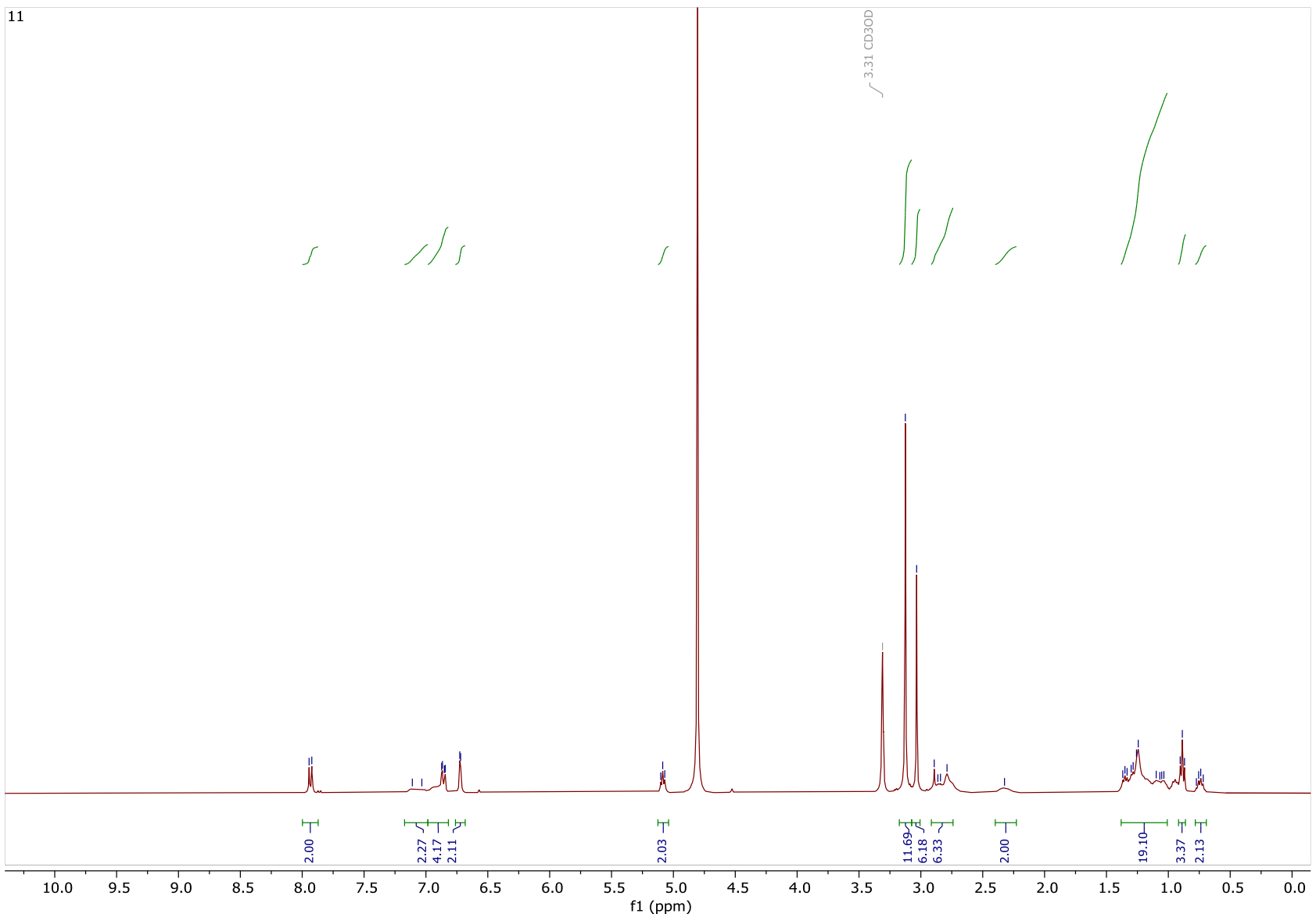

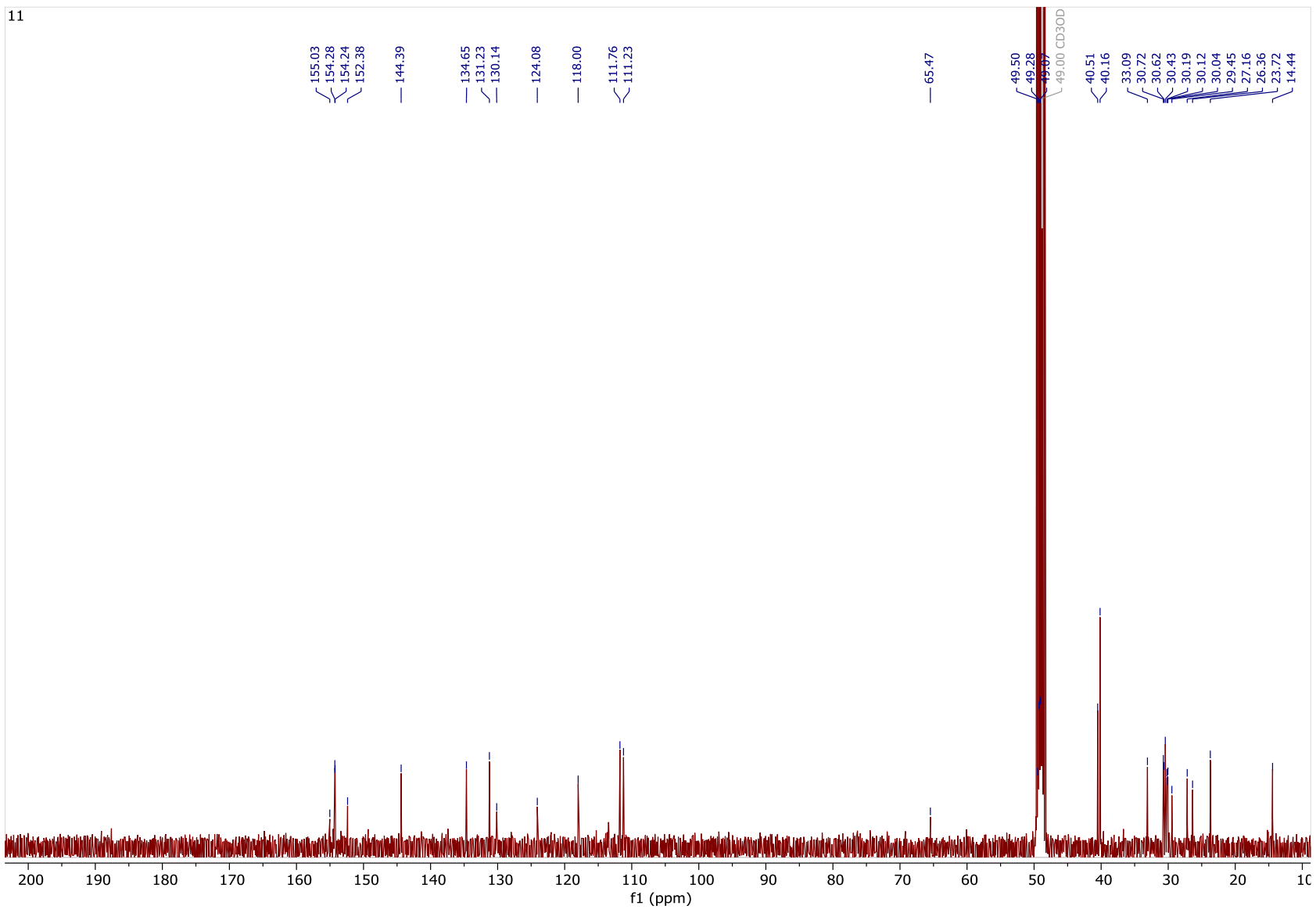

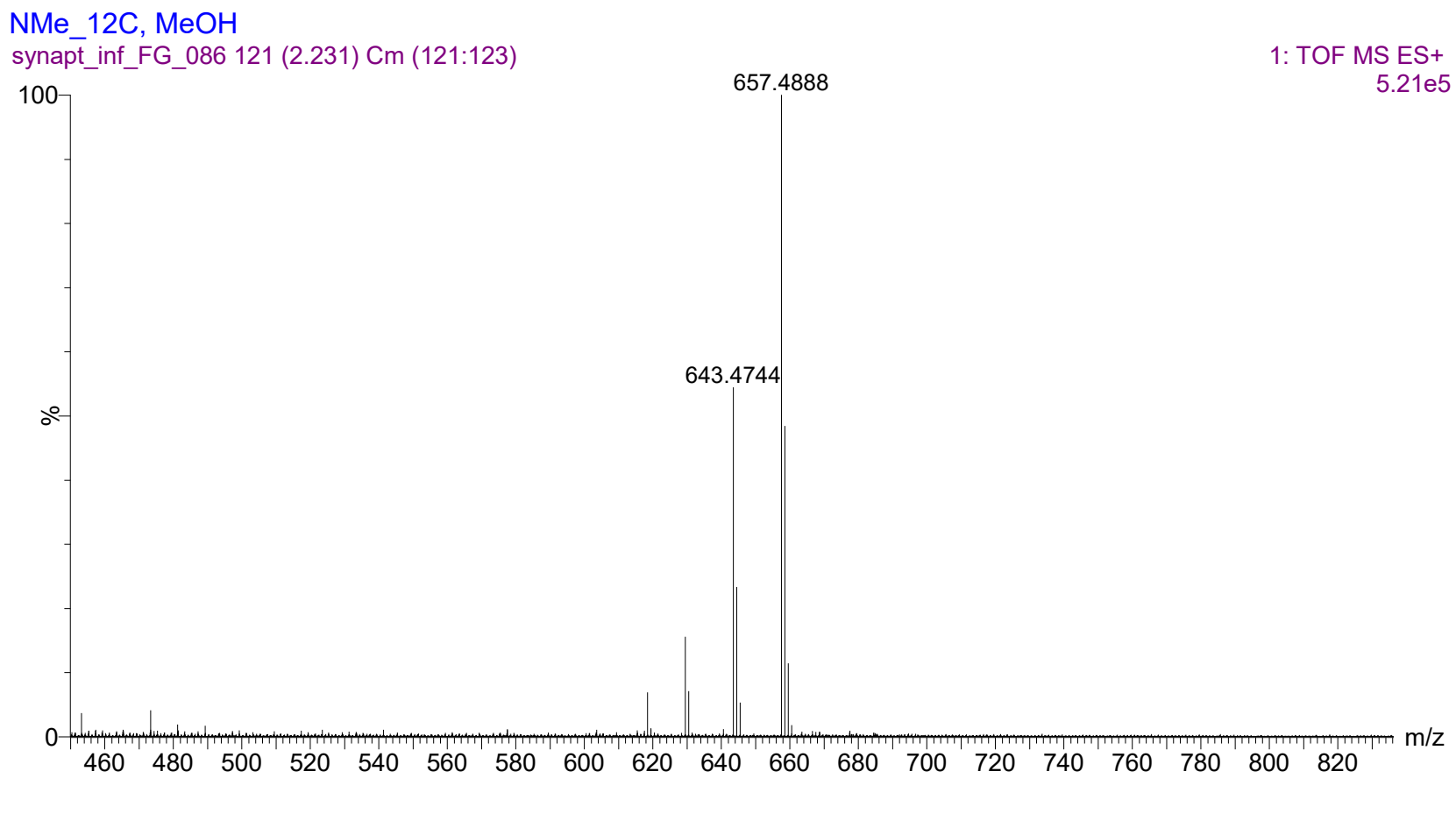

Figure S15. ^{1}H NMR, ^{13}C NMR (methanol-d_4) and mass spectra of compound **8**.

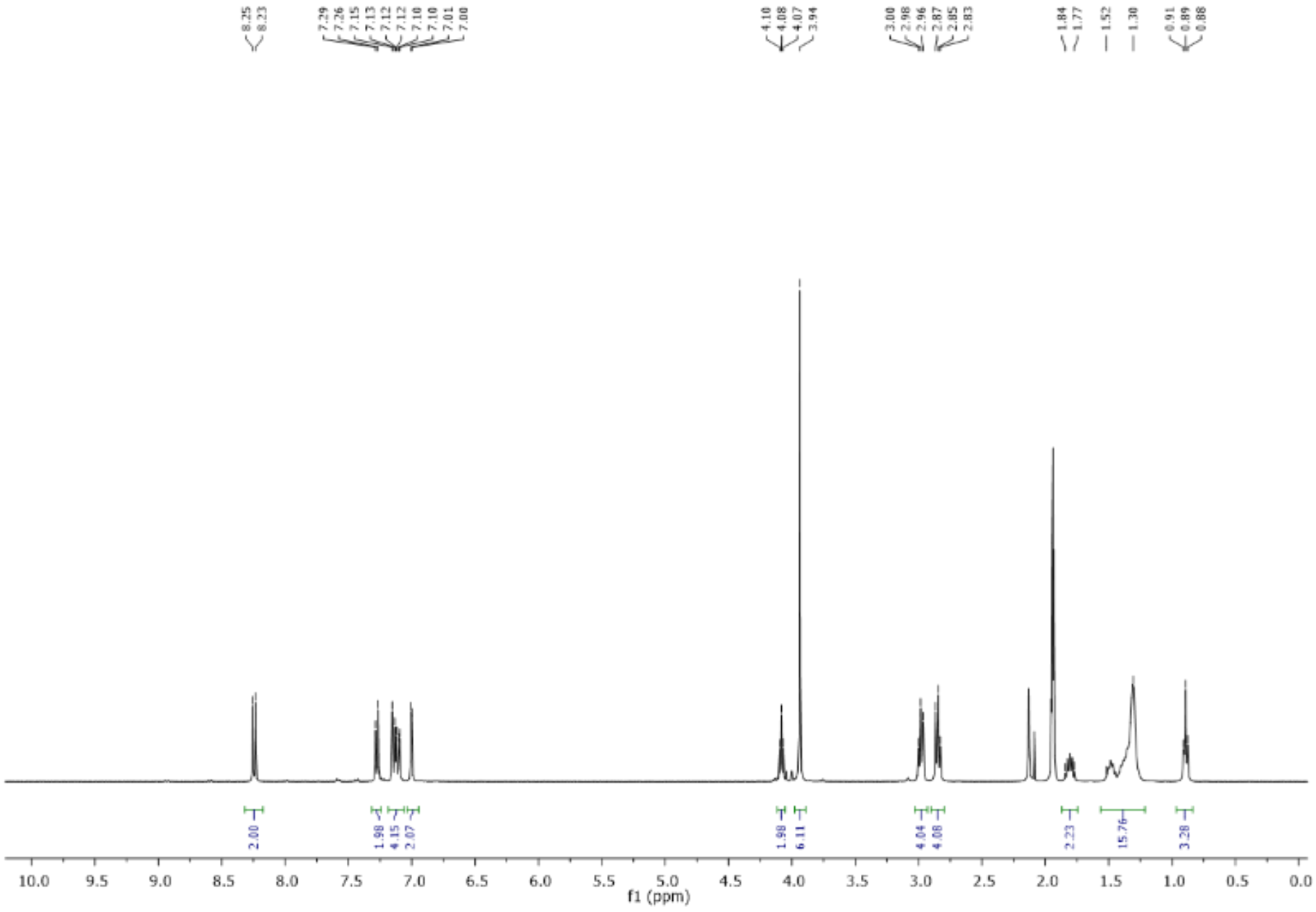

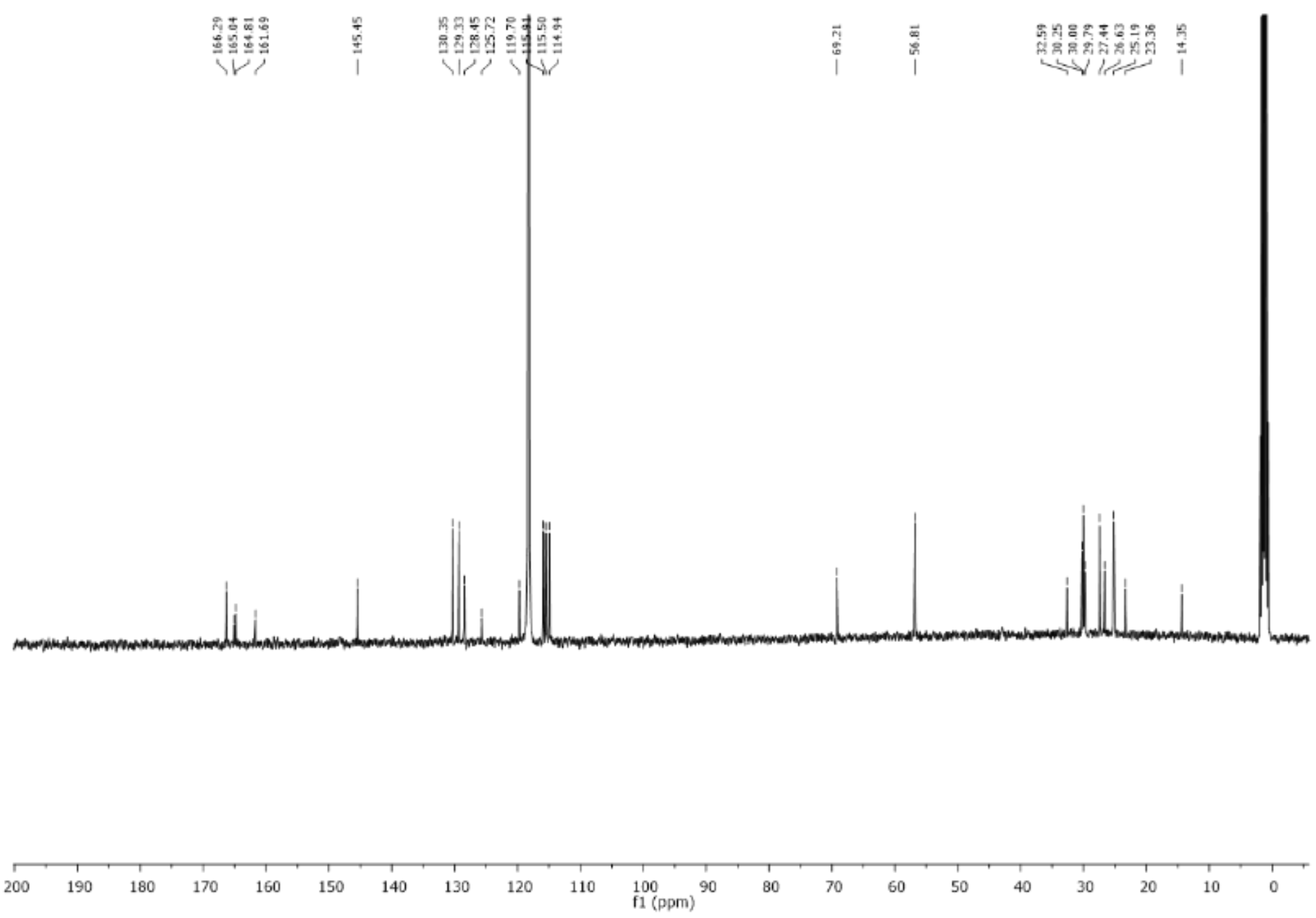

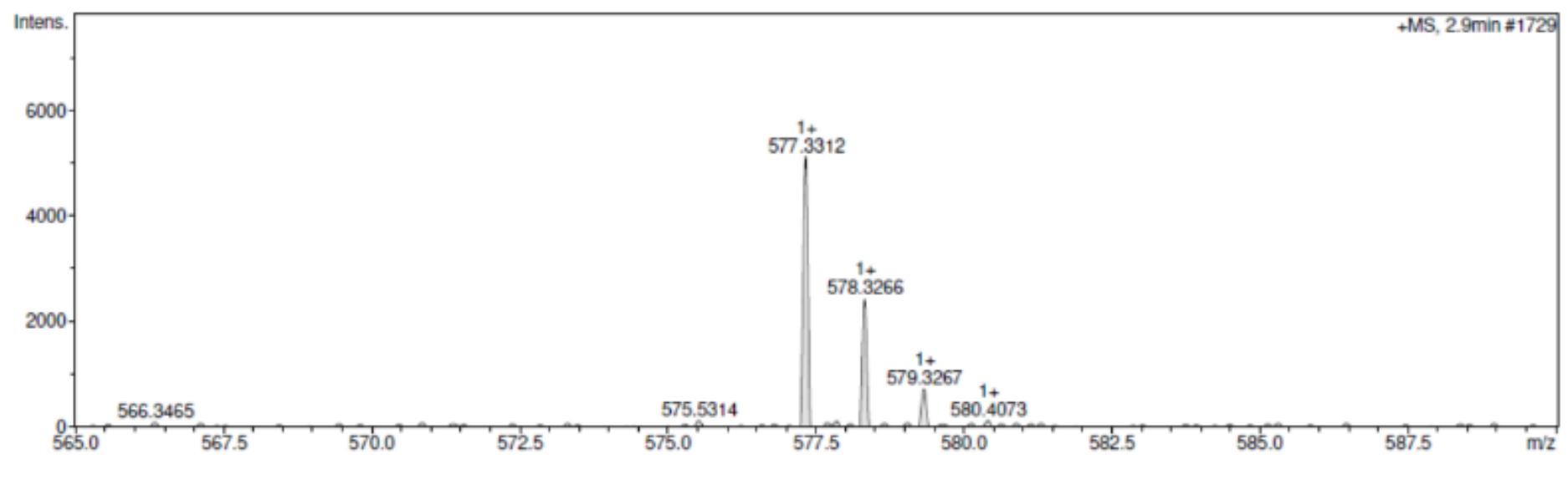

Meas. m/z	#	Ion Formula	Score	m/z	err [mDa]	err [ppm]	mSigma	rdb	e⁻ Conf	N-Rule	Adduct
577.3312	1	C40H41N4	59.52	577.3326	-1.4	-2.5	24.1	22.5	even	ok	M+H
	2	C39H45O4	100.00	577.3312	-0.1	-0.1	32.0	17.5	even	ok	M+H
	3	C35H41N6O2	14.36	577.3286	2.6	4.5	44.3	18.5	even	ok	M+H

Figure S16. ^{1}H NMR, ^{13}C NMR (CD_3CN) and mass spectra of compound **9a**.

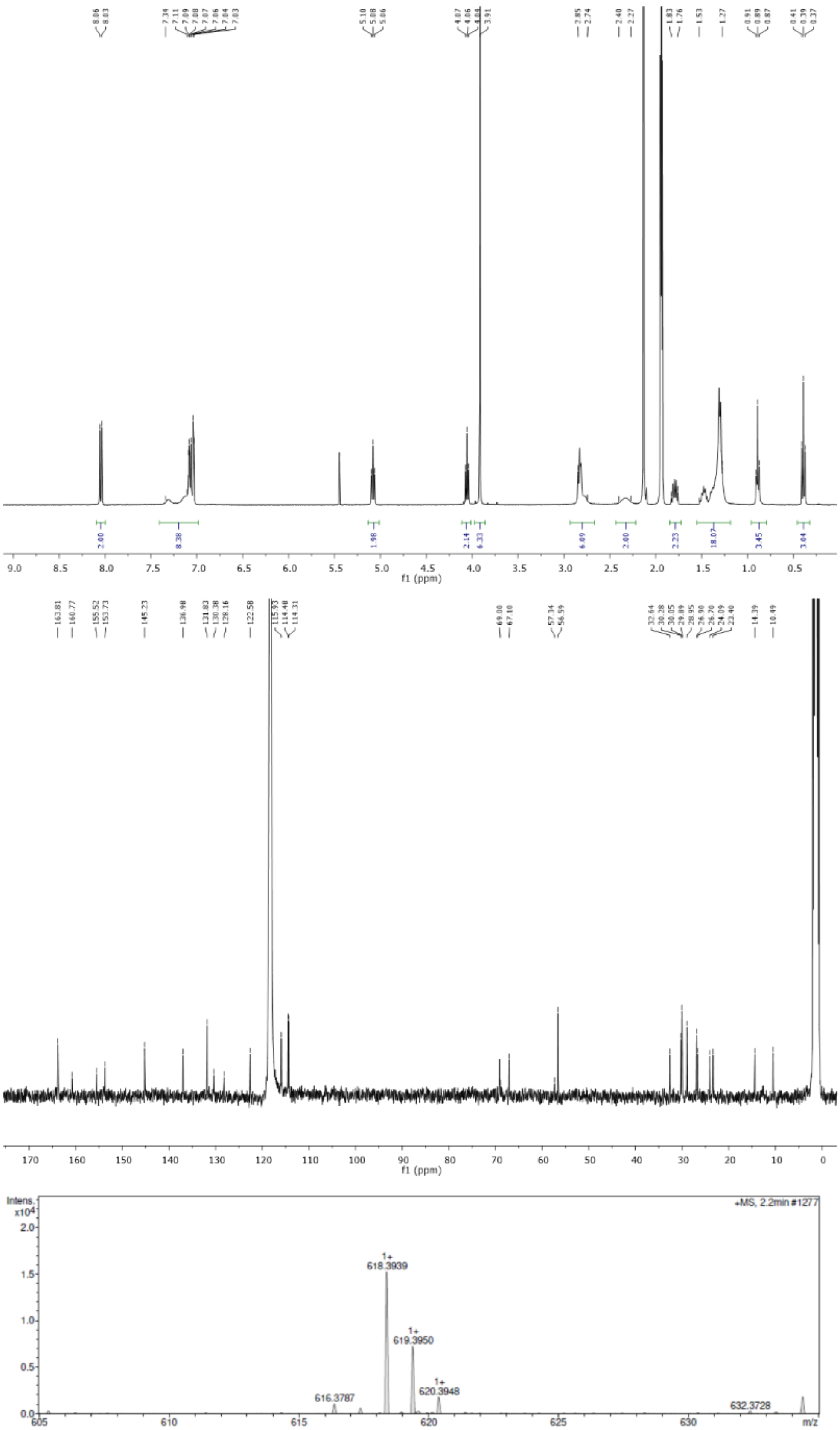

Meas. m/z	#	Ion Formula	Score	m/z	err [mDa]	err [ppm]	mSigma	rdb	e⁻ Conf	N-Rule	Adduct
618.3939	1	C42H52NO3	100.00	618.3942	-0.2	-0.4	6.9	17.5	even	ok	M+H
	2	C38H48N7O	19.28	618.3915	-2.4	-4.0	19.2	18.5	even	ok	M+H

Figure S17. H NMR, ^{13}C NMR (CD_3CN) and mass spectra of compound **9b**.

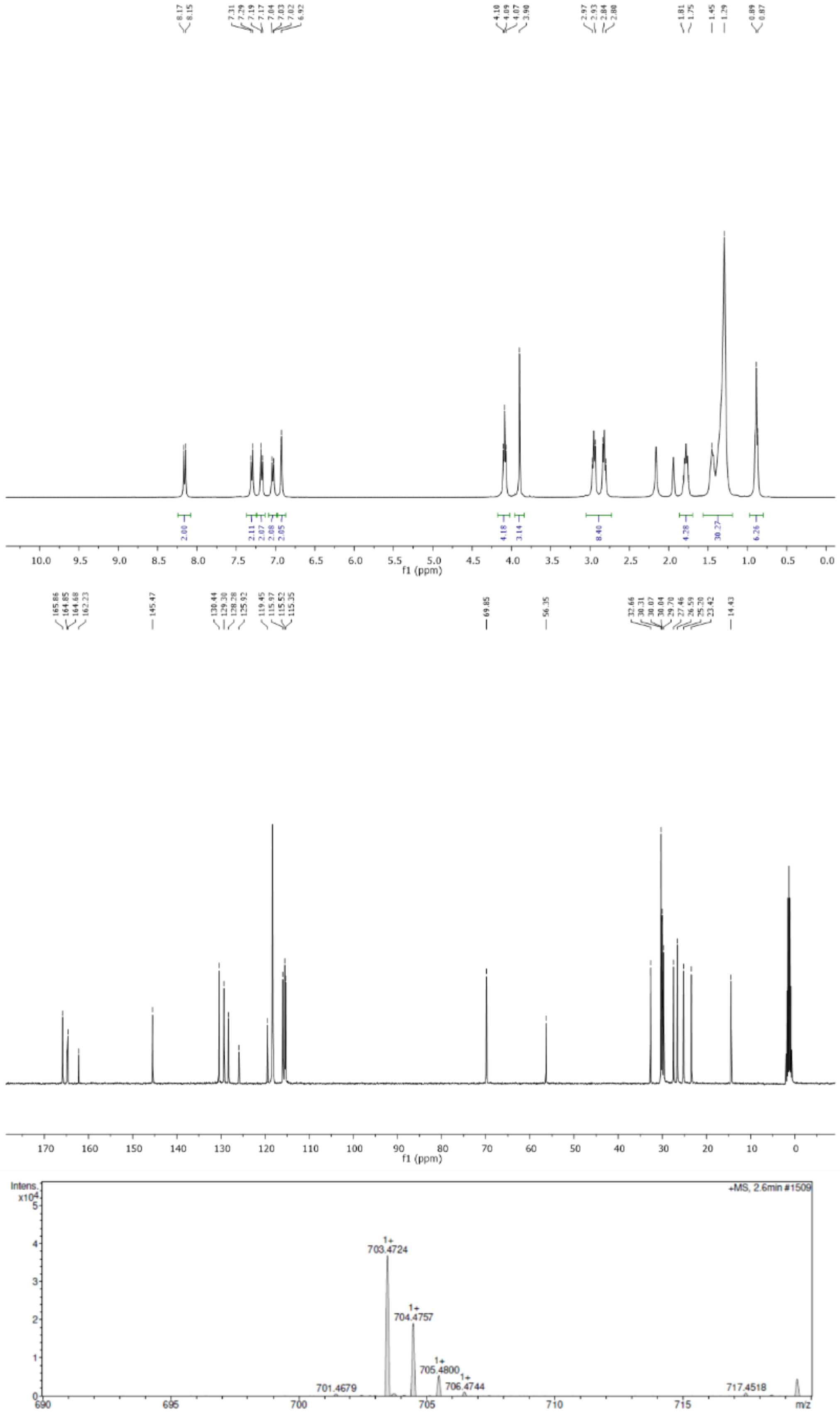

Meas. m/z	#	Ion Formula	Score	m/z	err [mDa]	err [ppm]	mSigma	rdb	e⁻ Conf	N-Rule	Adduct
703.4724	1	C48H63O4	100.00	703.4721	0.3	0.4	3.9	17.5	even	ok	M
	2	C49H59N4	42.90	703.4734	-1.1	-1.5	15.6	22.5	even	ok	M

Figure S18. ^{1}H NMR, ^{13}C NMR (CD_3CN) and mass spectra of compound **10a**.

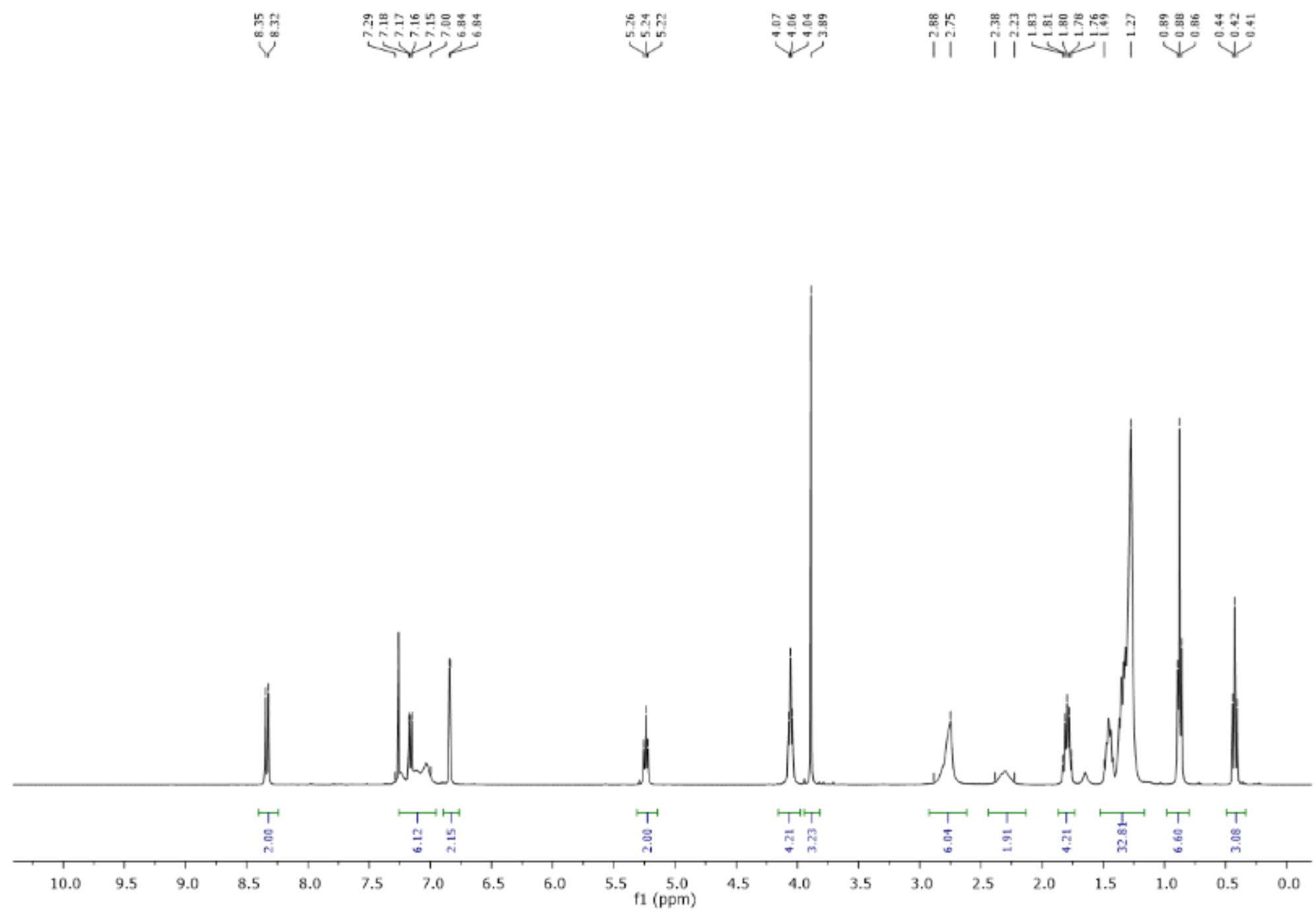

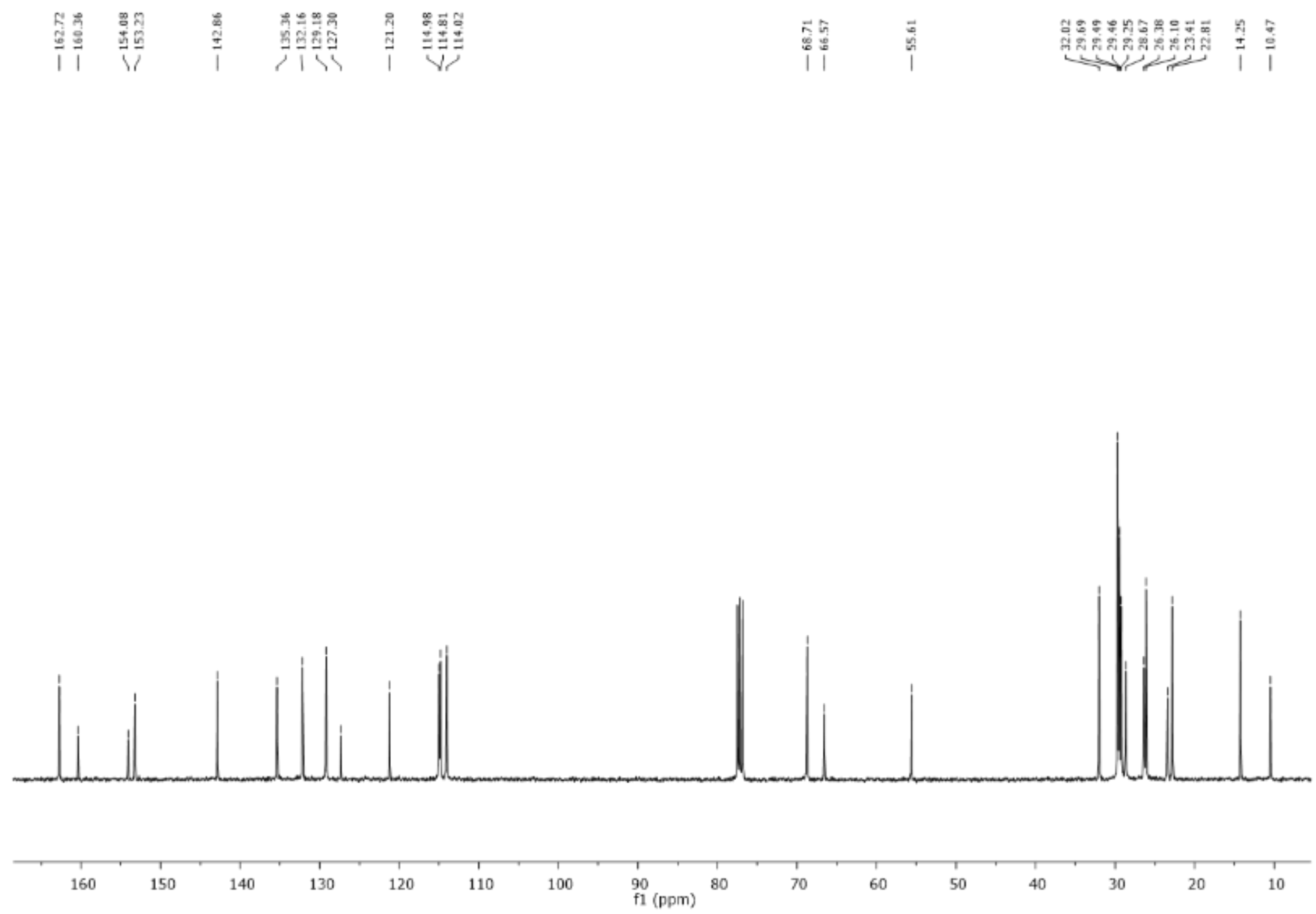

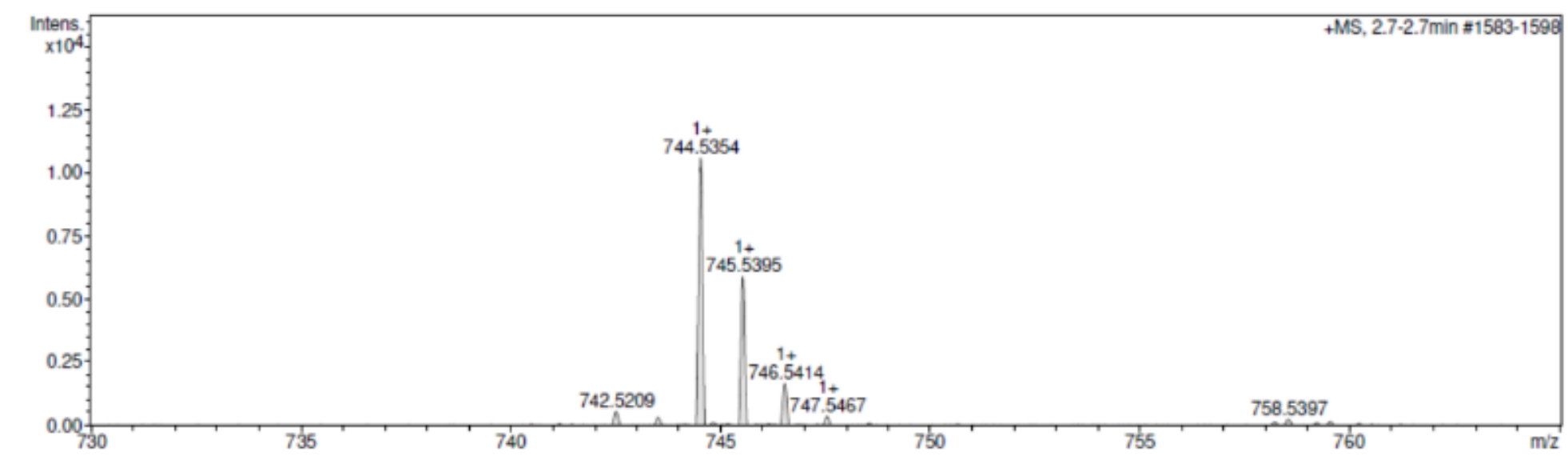

Meas. m/z	#	Ion Formula	Score	m/z	err [mDa]	err [ppm]	mSigma	rdb	e⁻ Conf	N-Rule	Adduct
744.5354	1	C51H70NO3	100.00	744.5350	0.4	0.5	4.2	17.5	even	ok	M+H
	2	C47H66N7O	10.46	744.5323	-3.1	-4.2	10.2	18.5	even	ok	M+H

Figure S19. ^{1}H NMR, ^{13}C NMR ($CDCl_3$) and mass spectra of compound **10b**.

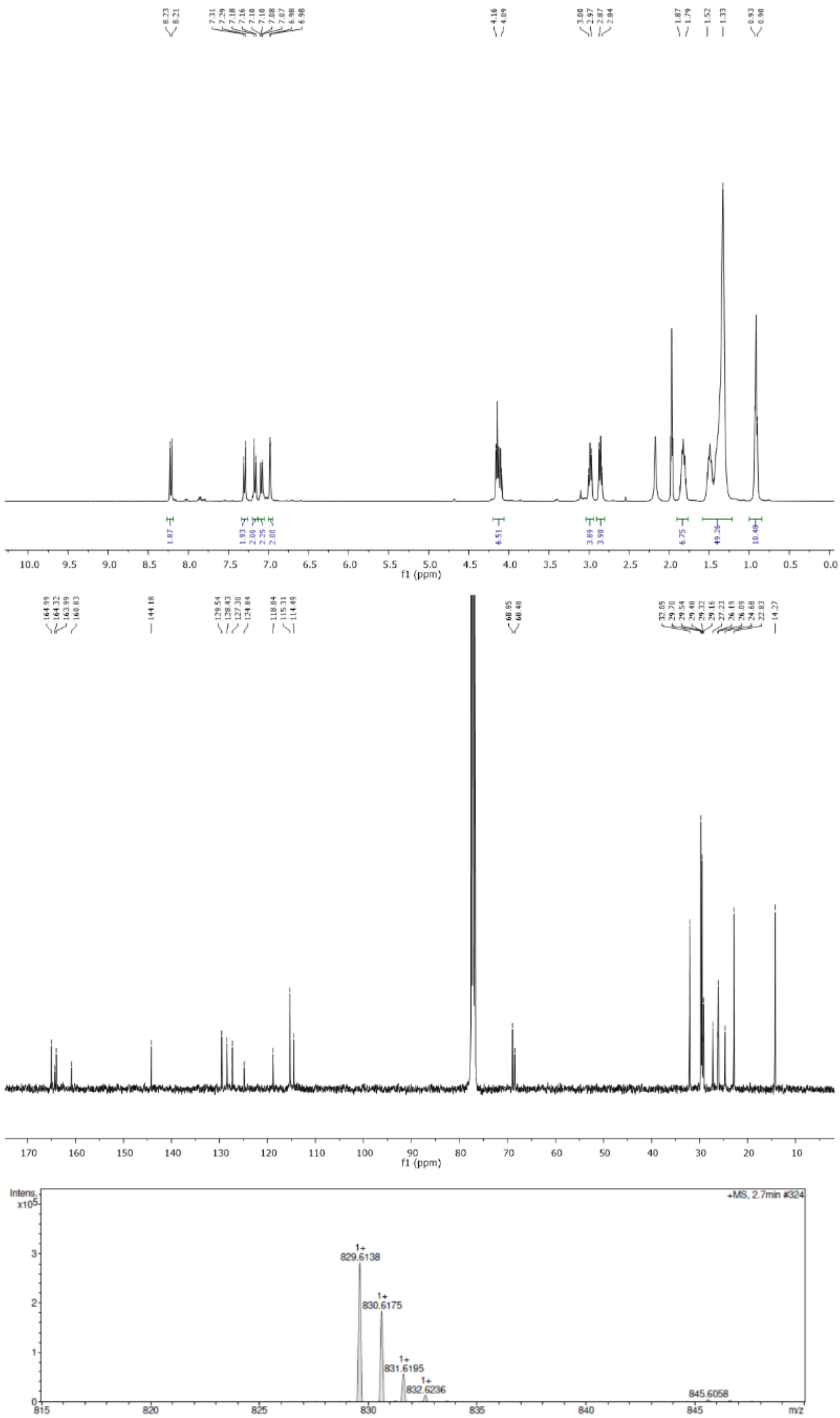

Meas. m/z	#	Ion Formula	Score	m/z	err [mDa]	err [ppm]	mSigma	rdb	e⁻ Conf	N-Rule	Adduct
829.6138	1	C58H77N4	100.00	829.6143	0.5	0.6	4.6	22.5	even	ok	M+H
	2	C60H79NO	41.08	829.6156	-1.8	-2.2	9.4	22.0	odd	ok	M+H
	3	C57H81O4	72.85	829.6129	-0.9	-1.0	11.2	17.5	even	ok	M+H
	4	C55H79N3O3	26.53	829.6116	2.2	2.7	16.6	18.0	odd	ok	M+H

Figure S20. ^{1}H NMR, ^{13}C NMR (CD_3CN) and mass spectra of compound **11a**.

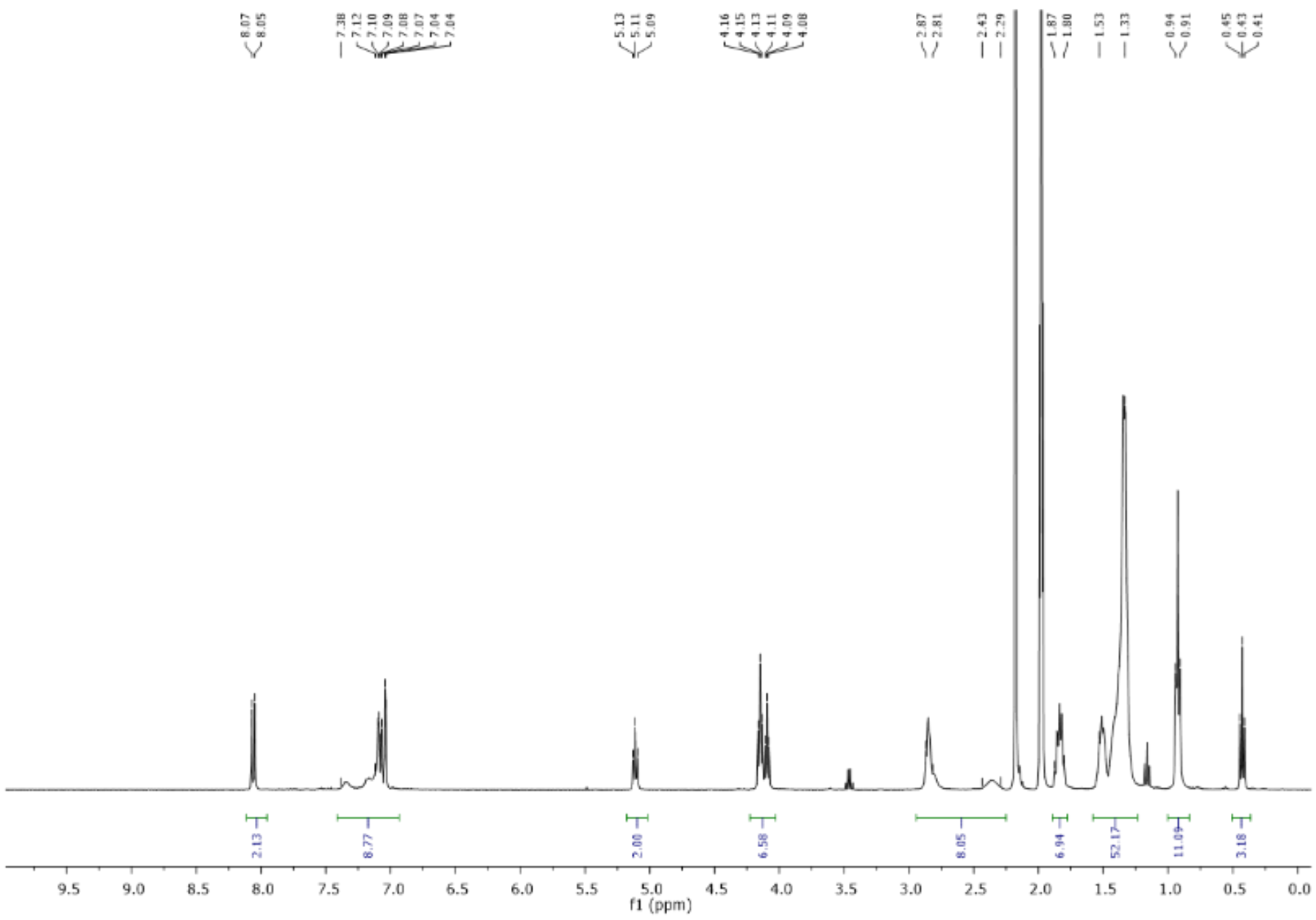

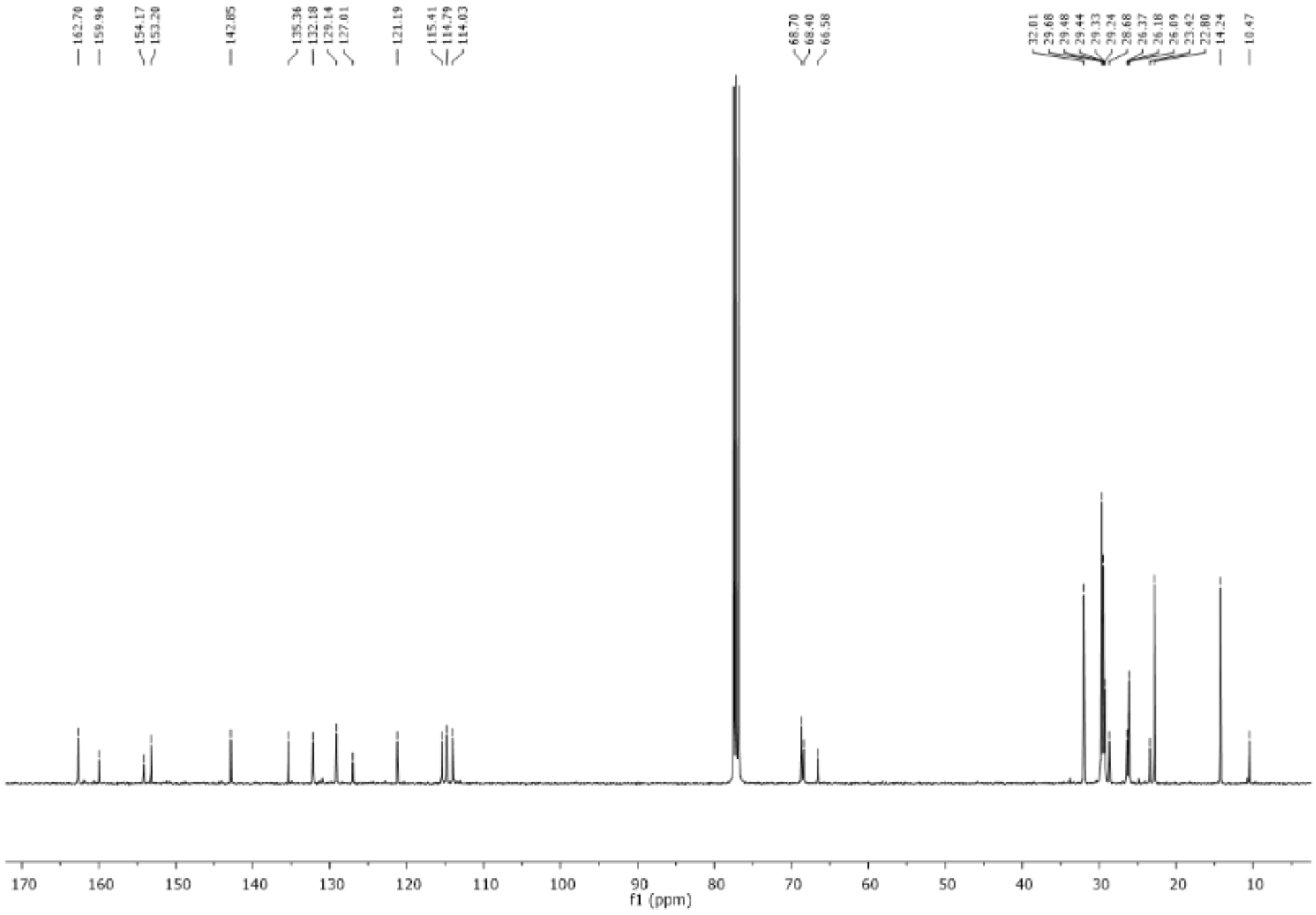

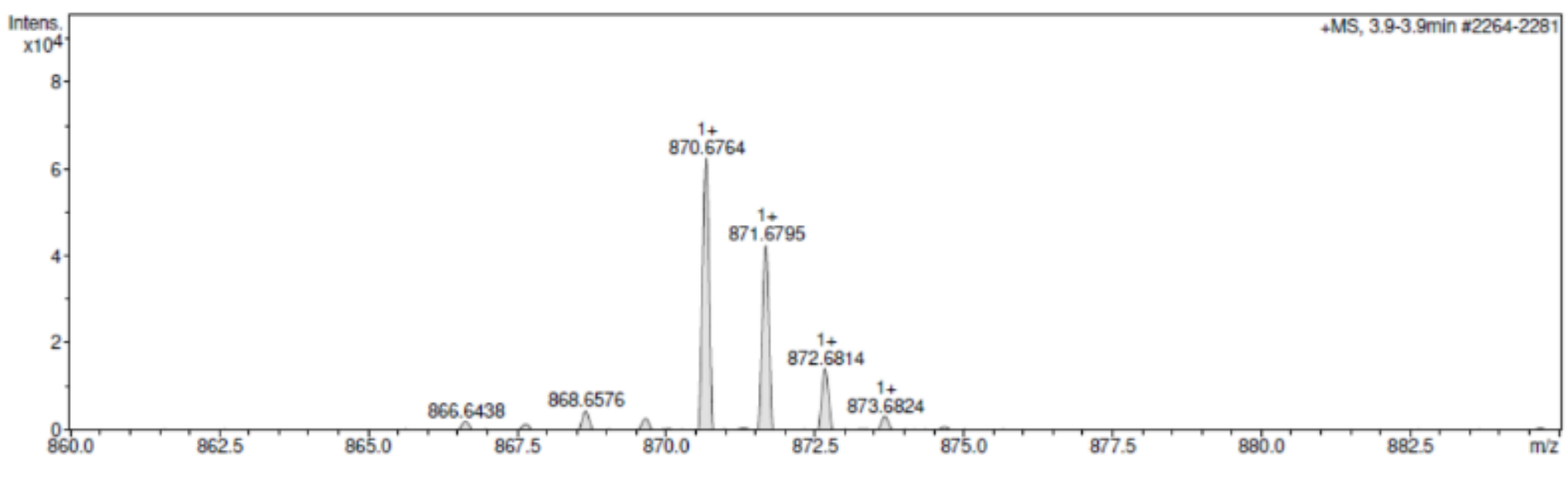

Meas. m/z	#	Ion Formula	Score	m/z	err [mDa]	err [ppm]	mSigma	rdb	e⁻ Conf	N-Rule	Adduct
870.6764	1	C60H88NO3	100.00	870.6759	-0.6	-0.6	7.0	17.5	even	ok	M
	2	C58H86N4O2	39.18	870.6745	-1.9	-2.2	13.1	18.0	odd	ok	M
	3	C56H84N7O	10.54	870.6732	3.2	3.7	19.6	18.5	even	ok	M
	4	C57H90O6	9.66	870.6732	3.2	3.7	23.9	13.0	odd	ok	M

Figure S21. ^{1}H NMR, ^{13}C NMR (CD_3CN) and mass spectra of compound **11b**.

Table S1. Photophysical properties of compounds in Group B. λ_{abs} and λ_{fluo}: absorption and emission maxima, respectively; log ε: logarithm of the molar absorption coefficient; Stokes shift expressed in wavenumbers and nanometers; Φ_f: fluorescence quantum yield; τ_f: fluorescence lifetime (ns); τ_f: fluorescence lifetime; k_r and k_{nr}: radiative and non-radiative rate constants, respectively.

COMP. (cLogP) (a)	S	λ_{abs} (nm)	Log ε	λ_{fluo} (nm)	Stokes shift (cm^{-1})	Stokes shift (nm)	Φ_f	τ_f (ns)	k_r (10^7 s^{-1})	k_{nr} (10^7 s^{-1})
**7** (8.57)	A(b)	425	4.27	544	5147	119	0.98	6.18	16	<1
	D(c)	436	4.30	547	4654	111	0.90	5.64	16	2
	P(d)	442	4.26	548	4376	106	0.93	<1.00	NC	NC
**8** (8.62)	A	510	4.30	652	4270	142	0.05	<1.00	NC	NC
	D	520	4.35	642	3654	122	0.85	<1.00	NC	NC
	P	541	4.21	674	3647	133	0.01	<1.00	NC	NC

Notes: (a) cLogP calculated using SwissADME[10]; (b) Acetonitrile; (c) Dichloromethane; (d) PBS (10 mM, pH 7.4); ND: not detected; NC: not calculated.

Table S2. Photophysical properties of compounds in Group C. λ_{abs} and λ_{fluo}: absorption and emission maxima, respectively; log ε: logarithm of the molar absorption coefficient; Stokes shift expressed in wavenumbers and nanometers; Φ_f: fluorescence quantum yield; τ_f: fluorescence lifetime (ns); τ_f: fluorescence lifetime; k_r and k_{nr}: radiative and non-radiative rate constants, respectively.

COMP. (cLogP) (a)	S	λ_{abs} (nm)	Log ε	λ_{fluo} (nm)	Stokes shift (cm^{-1})	Stokes shift (nm)	Φ_f	τ_f (ns)	k_r (10^7 s^{-1})	k_{nr} (10^7 s^{-1})
**9a** (7.53)	A(b)	493	4.36	550	2102	57	0.74	5.03	15	5
	D(c)	518	4.78	551	1156	33	0.79	4.93	16	4
	P(d)	505	4.36	566	2133	61	ND	<1.00	NC	NC
**9b** (8.48)	A	429	4.44	544	4928	115	0.95	6.09	16	1
	D	436	4.47	548	4688	112	0.95	5.63	17	1
	P	438	4.36	555	4813	117	0.2	1.74	11	46
**10a** (10.23)	A	497	4.41	556	2135	59	0.76	5.03	15	5
	D	511	4.68	556	1584	45	0.71	4.77	15	6
	P	482	4.42	566	3079	84	ND	ND	NC	NC
**10b** (11.22)	A	429	4.47	550	5128	121	0.94	5.88	16	1
	D	441	4.49	552	4560	111	0.92	5.96	15	1
	P	431	4.36	547	4920	116	0.1	1.86	5	48
**11a** (12.97)	A	493	4.30	555	2266	62	0.75	4.97	15	5
	D	517	4.63	556	1357	39	0.74	4.77	16	5
	P	489	4.33	581	3243	92	ND	ND	NC	NC
**11b** (13.91)	A	428	4.44	550	5183	122	0.93	5.82	16	1
	D	440	4.50	552	4611	112	0.92	5.42	17	1
	P	433	4.42	547	4813	114	0.24	1.74	14	44

Notes: (a) cLogP calculated using SwissADME[10]; (b) Acetonitrile; (c) Dichloromethane; (d) PBS (10 mM, pH 7.4); ND: not detected; NC: not calculated.

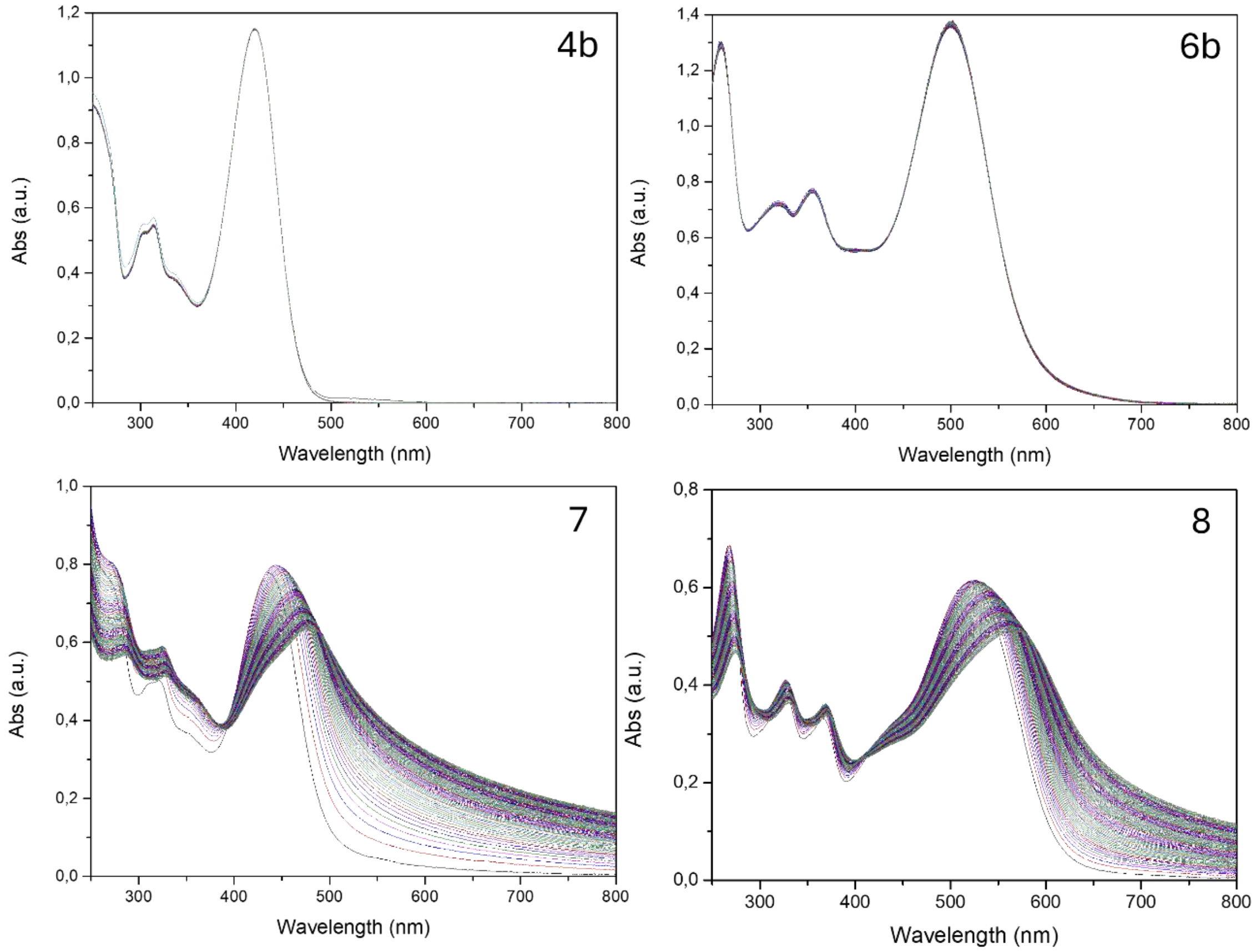

Figure S22. Time-dependent UV–Vis absorption spectra of compounds 4b, 6b, 7 and 8 showing aggregate formation in PBS (10 mM, pH 7.4; Δt = 20 s, total time = 30 min).

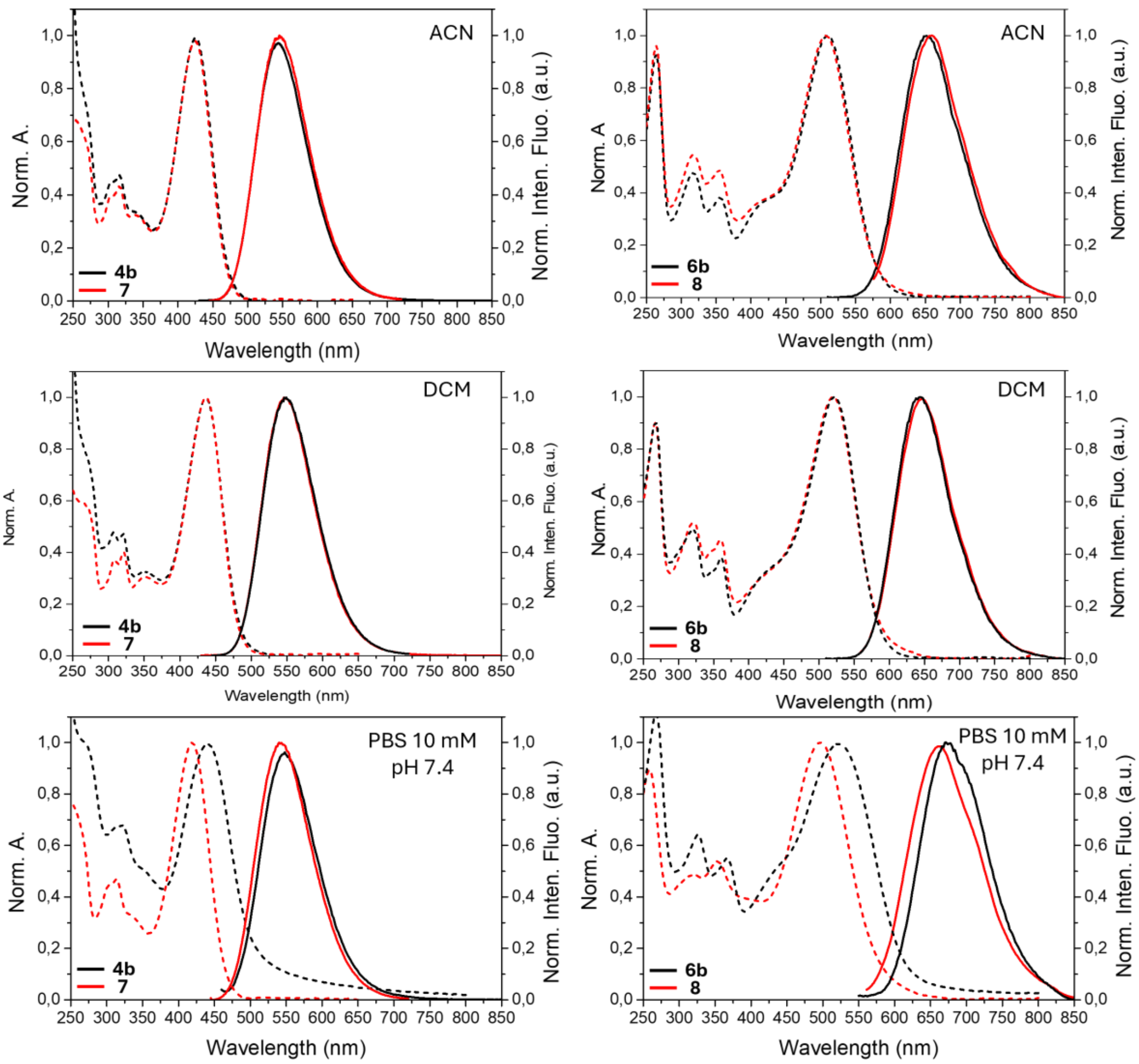

Figure S23. Comparative absorption and fluorescence emission spectra of compounds **4b**, **6b**, **7**, and **8** recorded in different solvents.

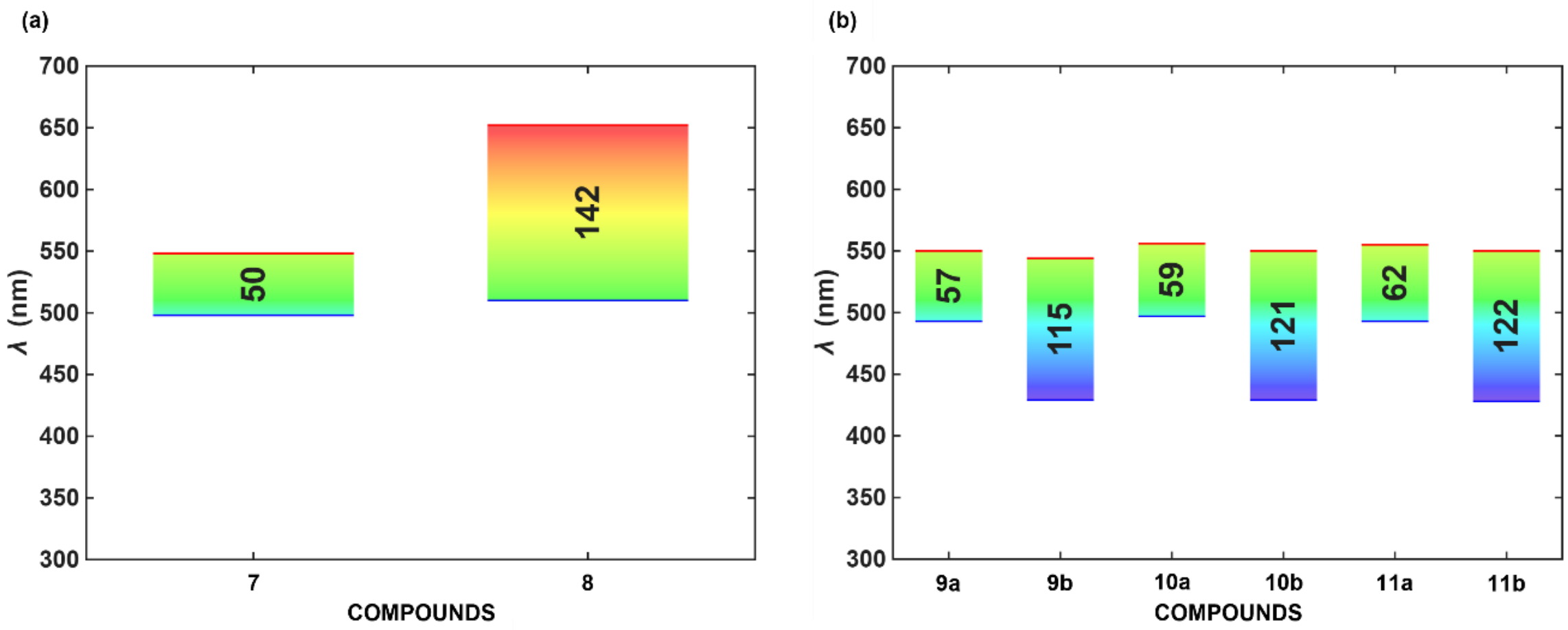

Figure S24. Stokes Shifts of groups **A** and **C**.

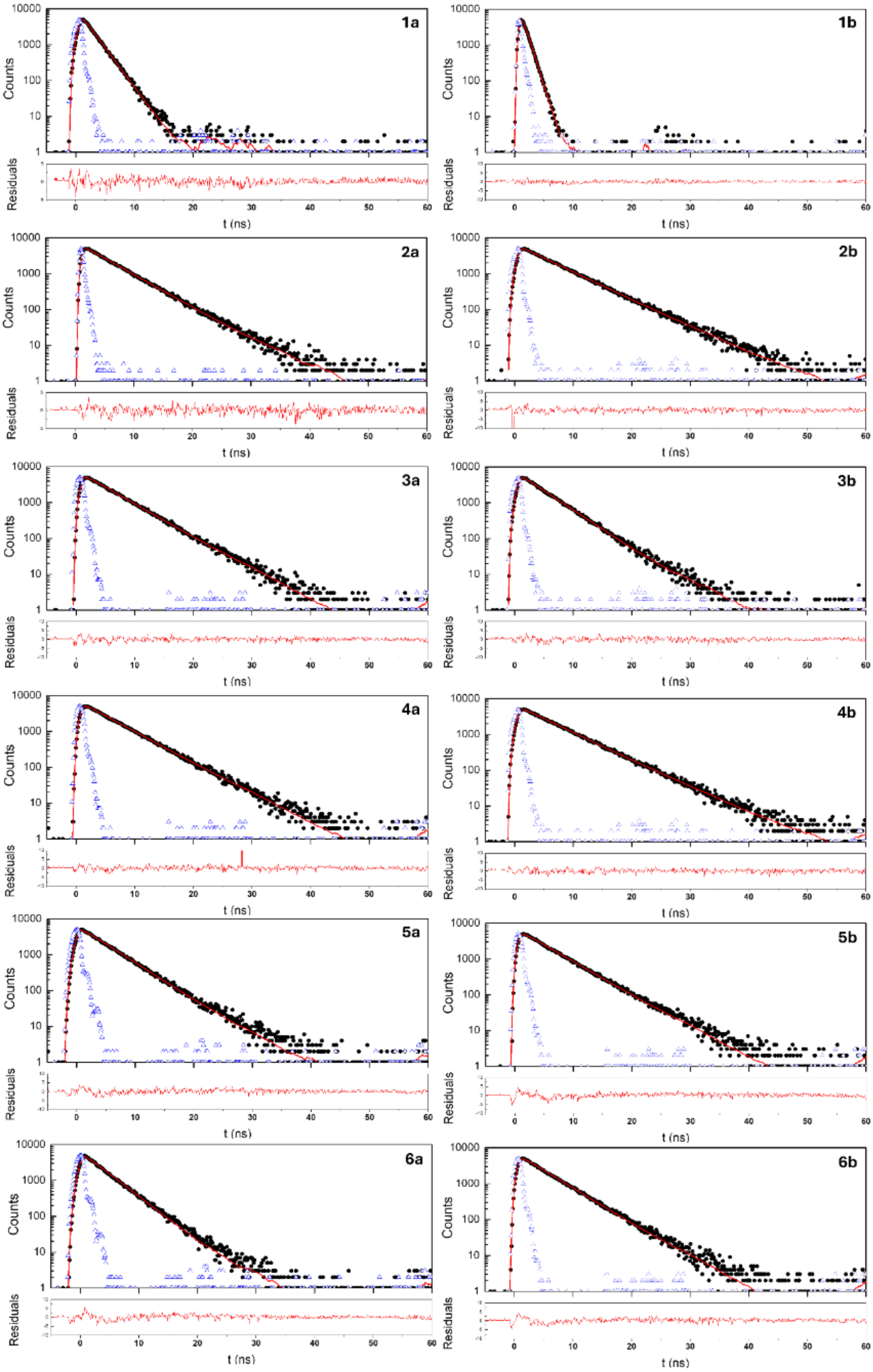

Figure S25. Fluorescence lifetime measurements of compounds **1–6a** and **1–6b** in dichloromethane determined by TCSPC. For each sample, the plot shows the signal of the excitation source (instrument response function, blue triangles), the emission decay of the solution recorded at the corresponding emission wavelength (black circles), the monoexponential fit obtained from the analysis (upper red line), and the residuals of the fit (bottom red line). Excitation and detection wavelengths were selected individually for each compound according to its absorption and emission maxima.

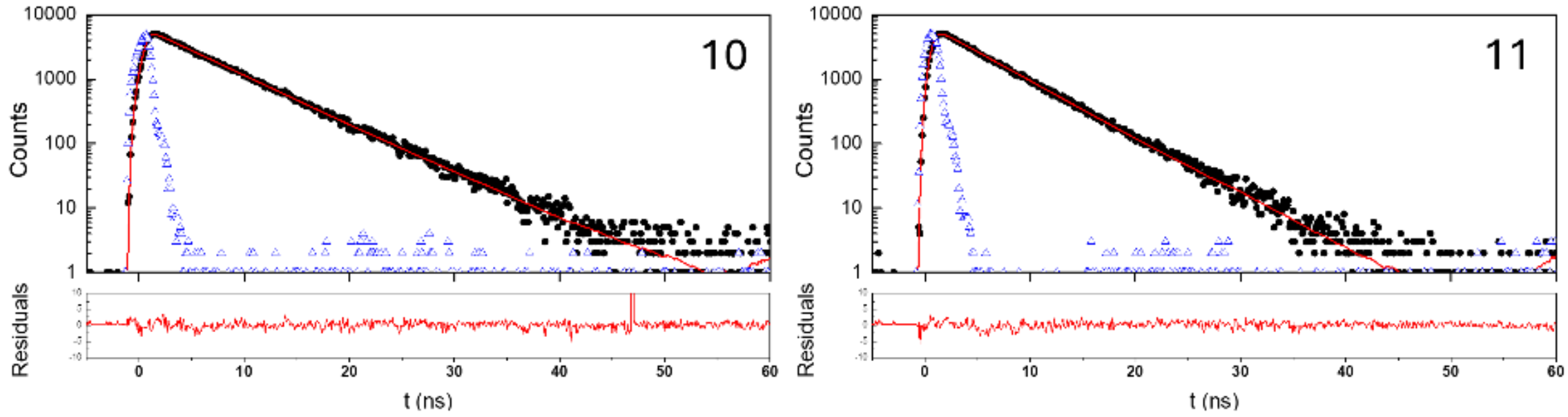

Figure S26. Fluorescence lifetime measurements of compounds 10 and 11 in dichloromethane determined by TCSPC. For each sample, the plot shows the signal of the excitation source (instrument response function, blue triangles), the emission decay of the solution recorded at the corresponding emission wavelength (black circles), the monoexponential fit obtained from the analysis (upper red line), and the residuals of the fit (bottom red line). Excitation and detection wavelengths were selected individually for each compound according to its absorption and emission maxima.

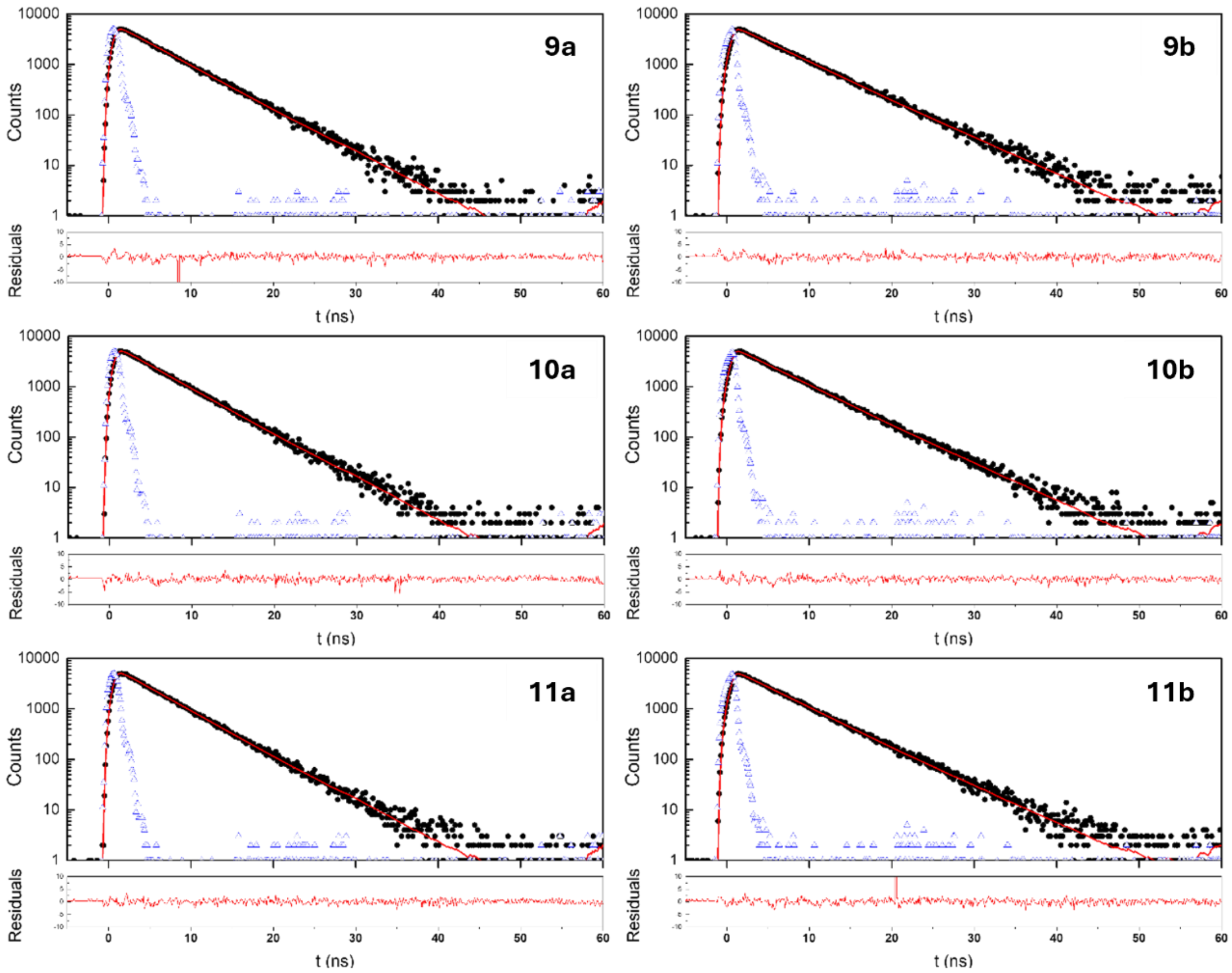

Figure S27. Fluorescence lifetime measurements of compounds **9-11a** and **9-11b** in dichloromethane determined by TCSPC. For each sample, the plot shows the signal of the excitation source (instrument response function, blue triangles), the emission decay of the solution recorded at the corresponding emission wavelength (black circles), the monoexponential fit obtained from the analysis (upper red line), and the residuals of the fit (bottom red line). Excitation and detection wavelengths were selected individually for each compound according to its absorption and emission maxima.

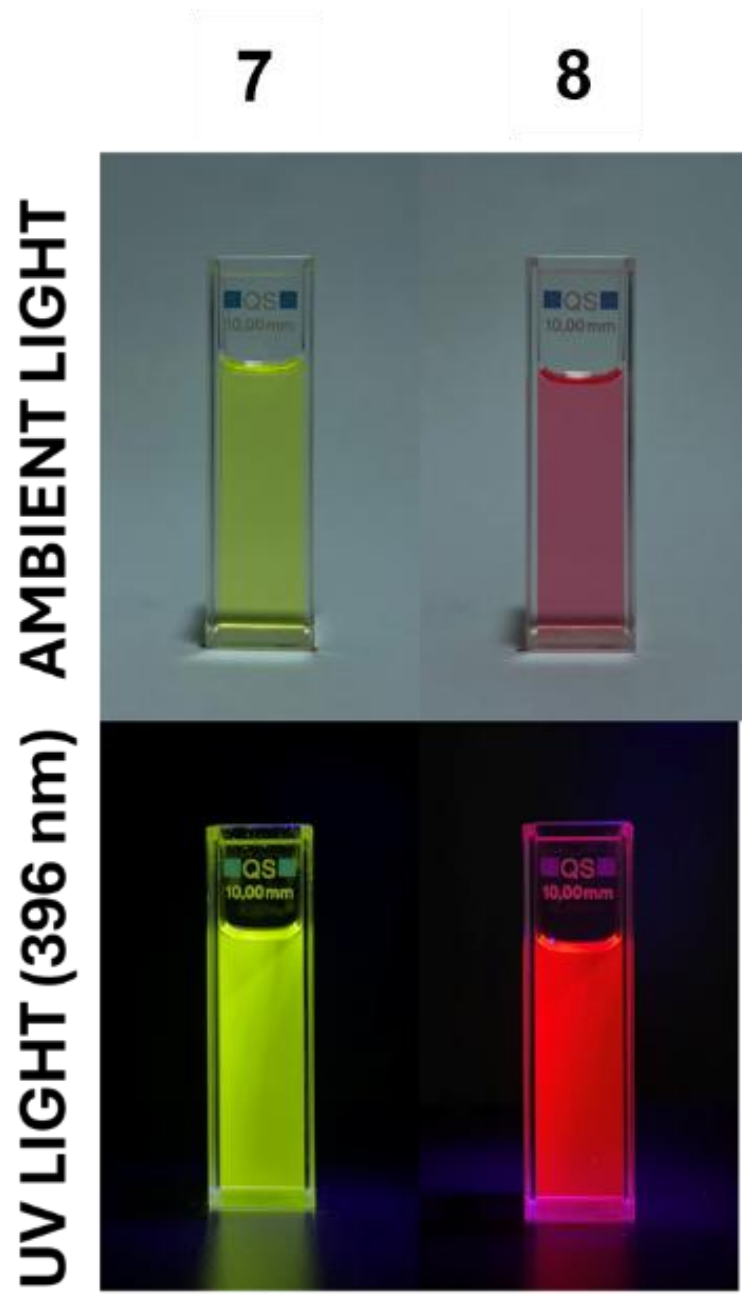

Figure S28. Photographs of dyes **7** and **8** in DCM under ambient light (top rows) and UV illumination (bottom rows).

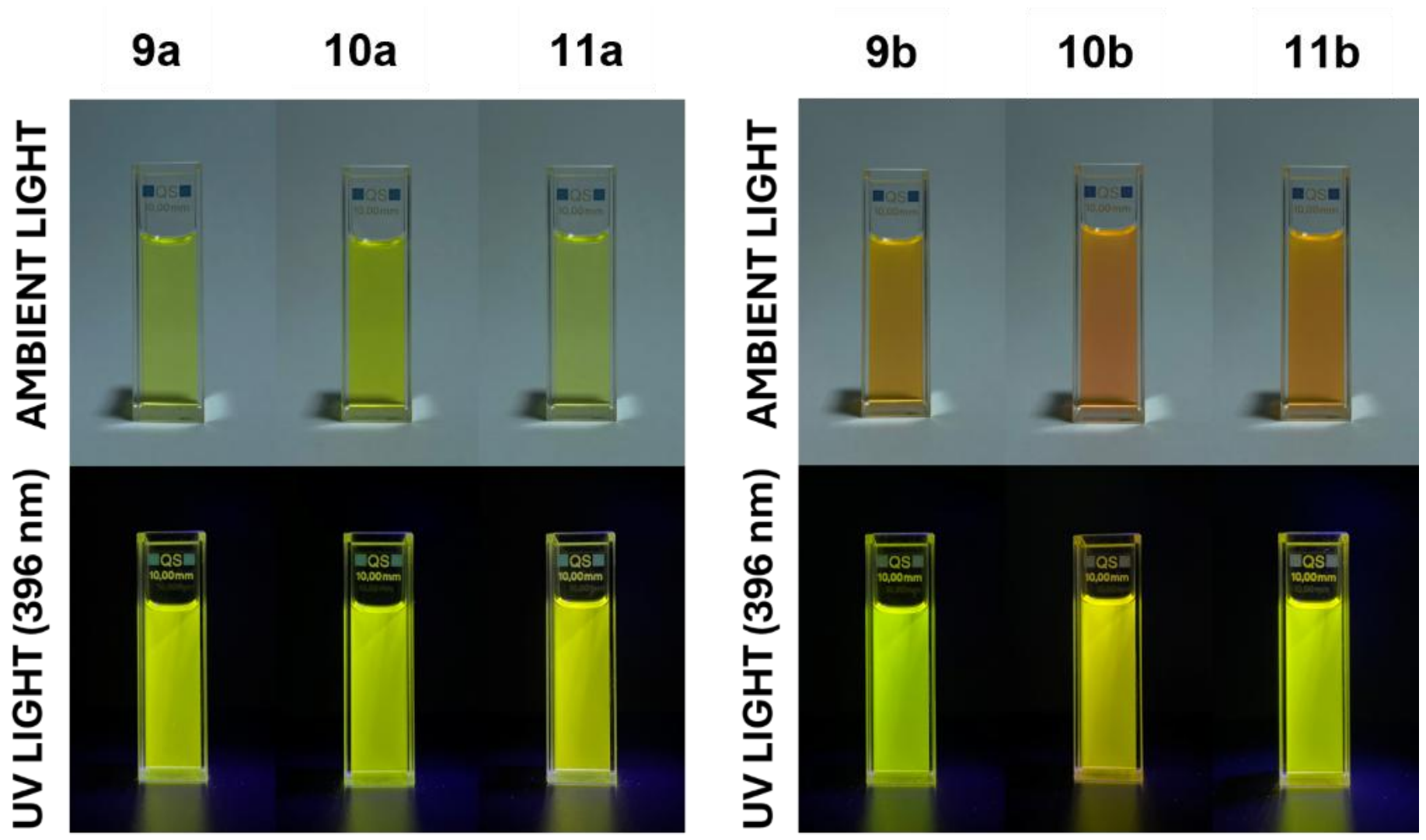

Figure S29. Photographs of dyes **9-11a** and **9-11b** in DCM under ambient light (top rows) and UV illumination (bottom rows).

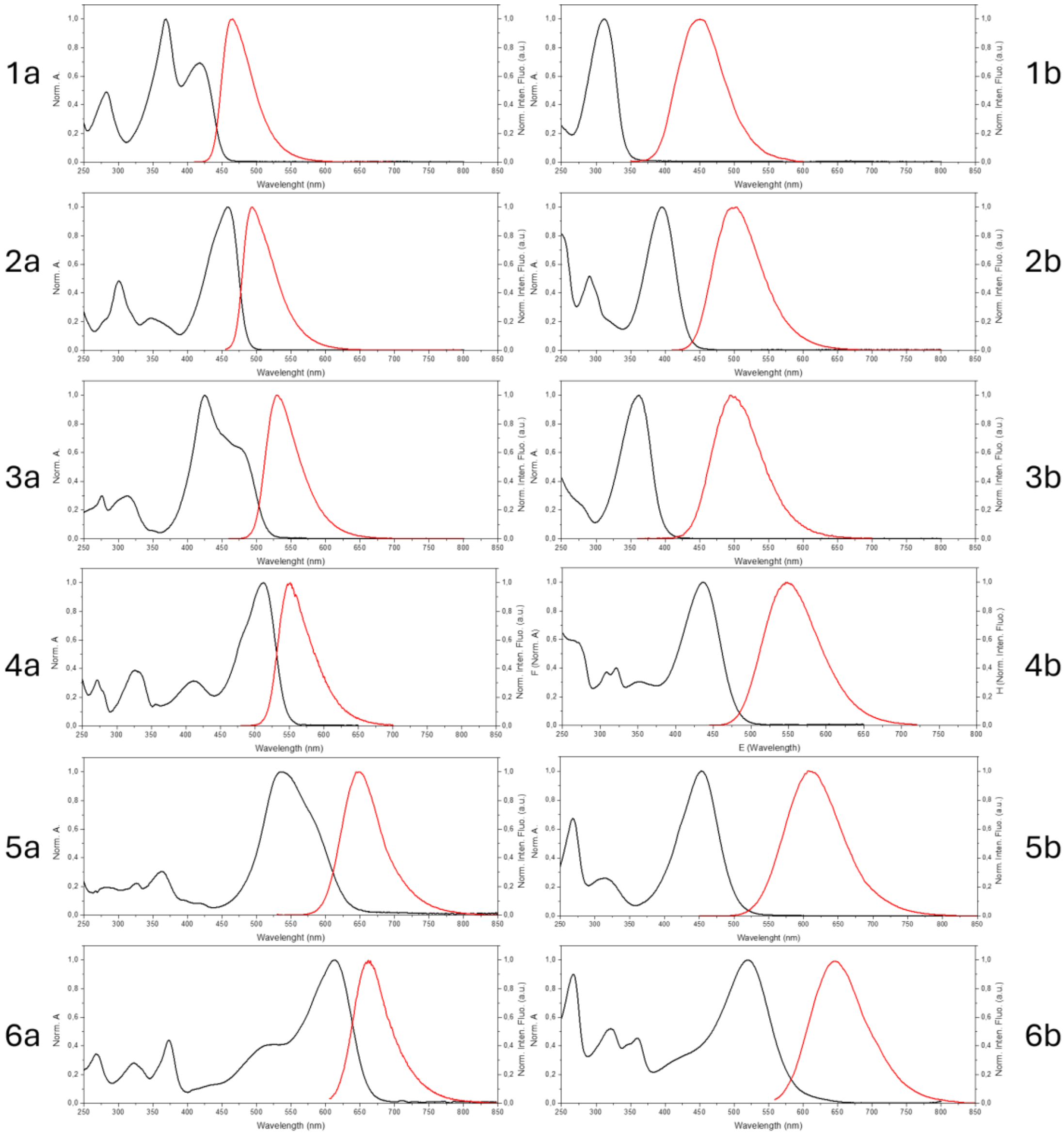

Figure S30. Normalized absorption and emission spectra of triarylpyrylium and triarylpyridinium dyes (**1-6a** and **1-6b**) in DCM.

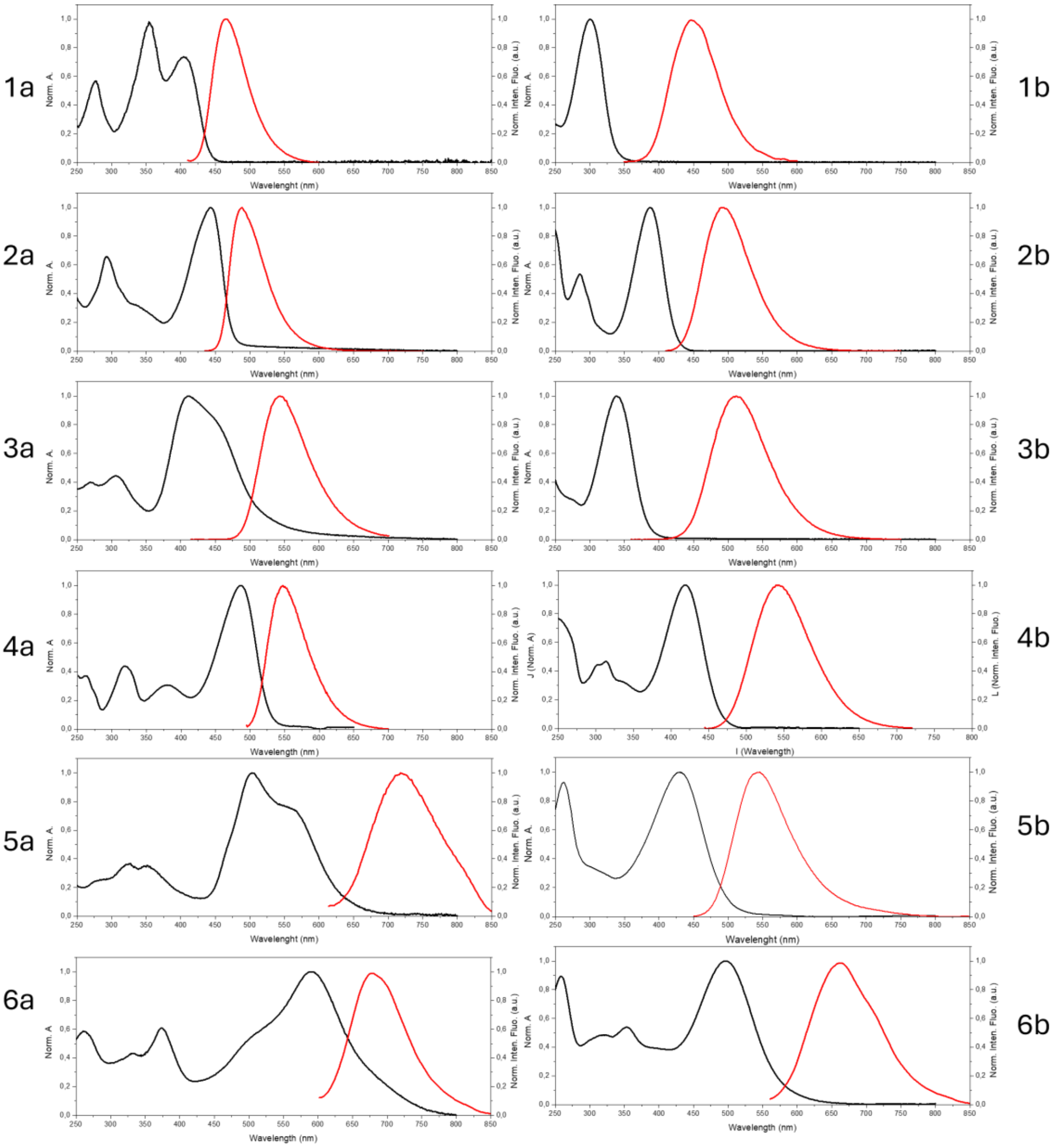

Figure S31. Normalized absorption and emission spectra of triarylpyrylium and triarylpyridinium dyes (**1-6a** and **1-6b**) in PBS.

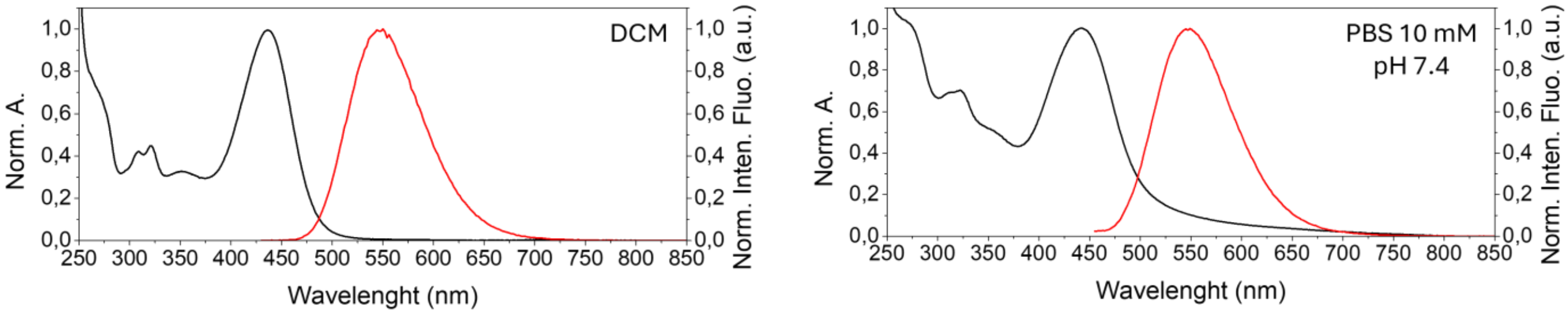

Figure S32. Normalized absorption and emission spectra of compound **7** in DCM and PBS.

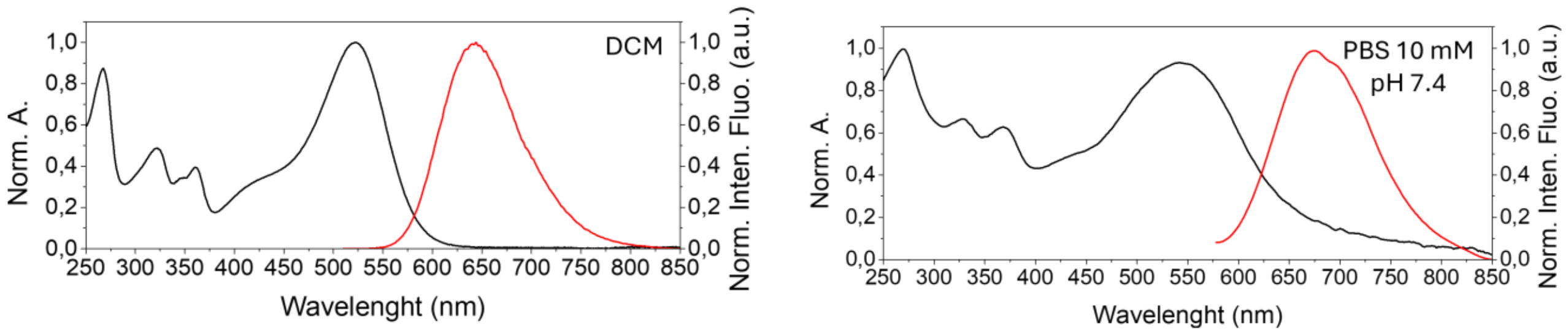

Figure S33. Normalized absorption and emission spectra of compound **8** in DCM and PBS.

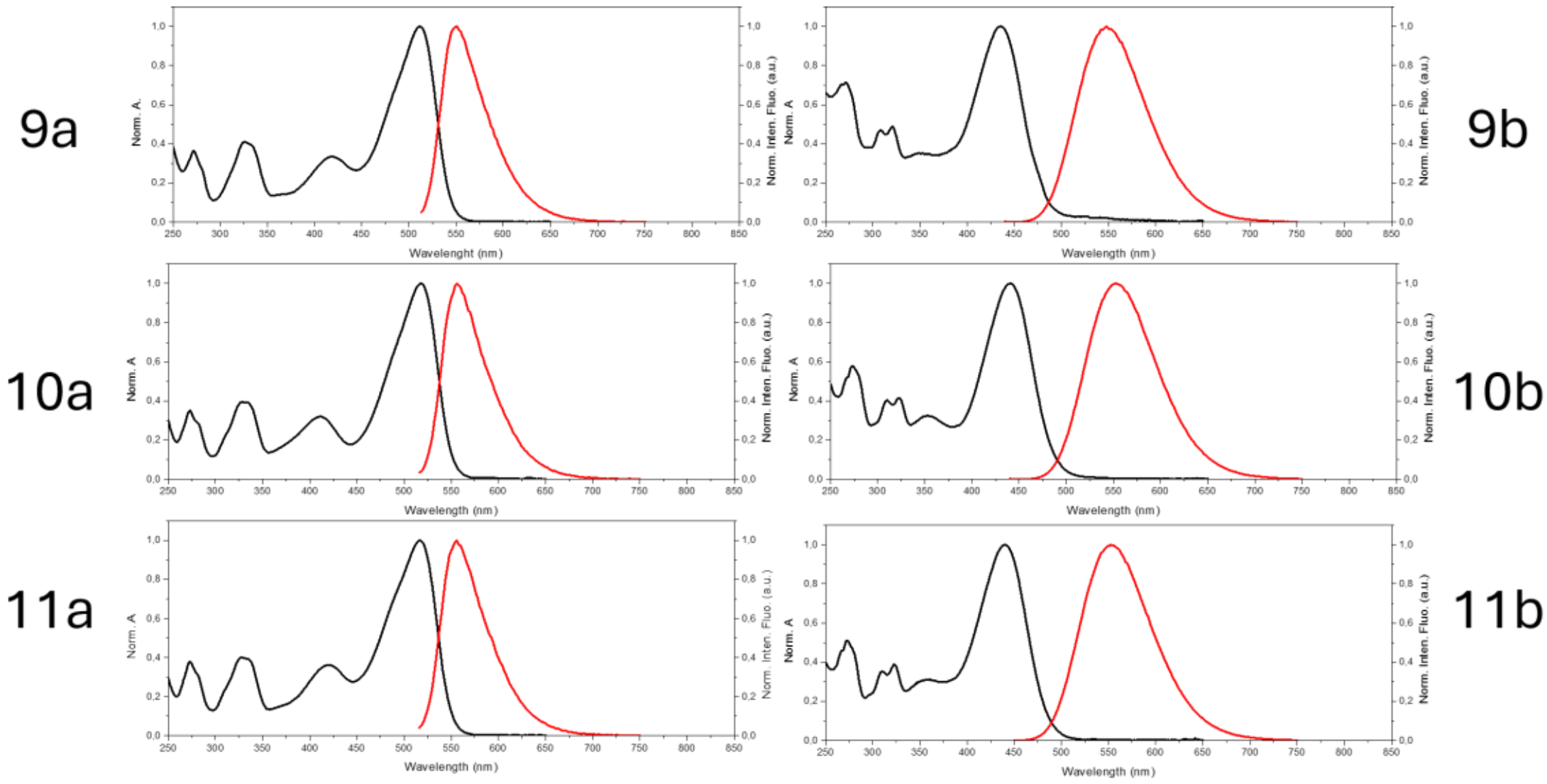

Figure S34. Normalized absorption and emission spectra of triarylpyrylium and triarylpyridinium dyes (**9-11a** and **9-11b**) in DCM.

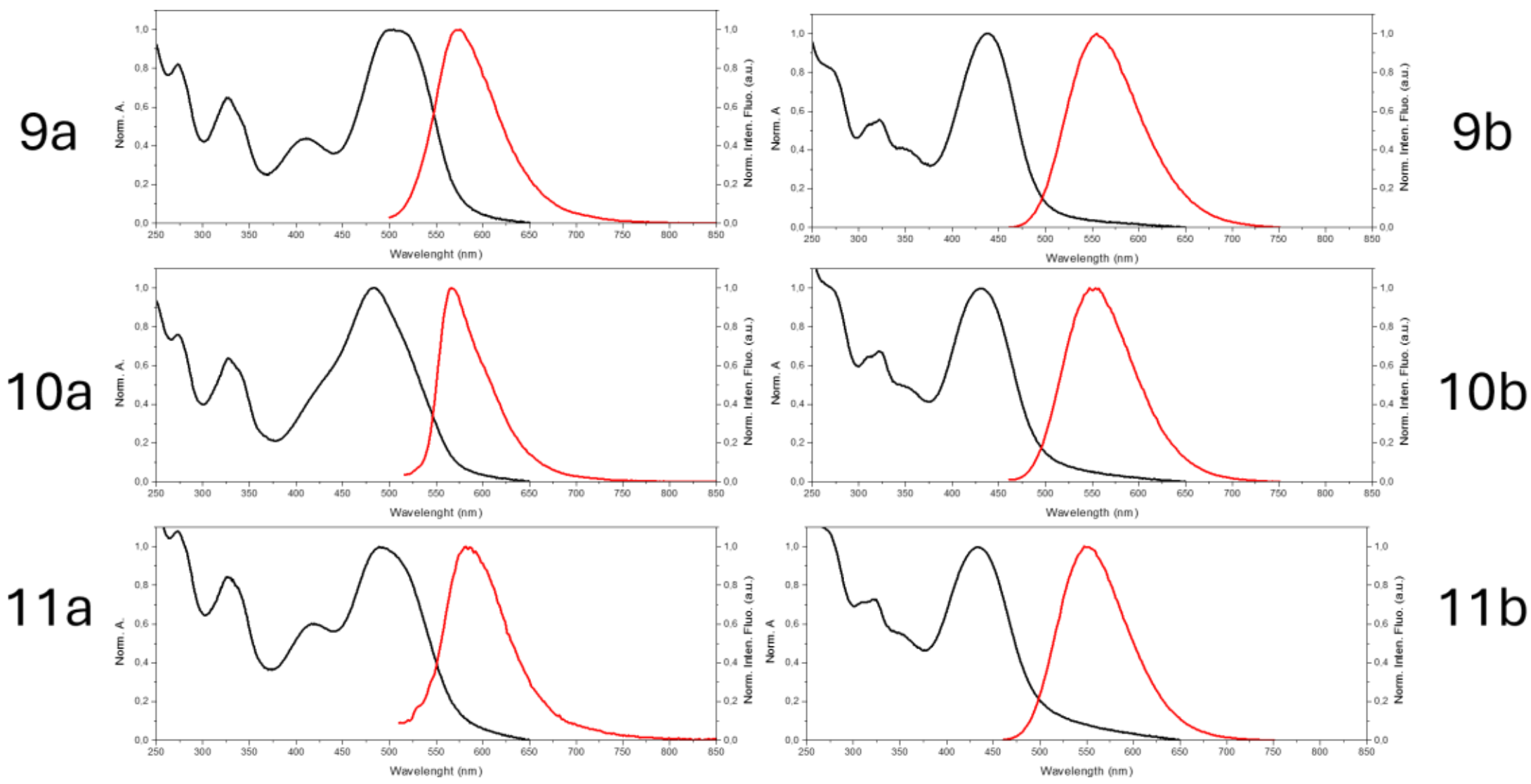

Figure S35. Normalized absorption and emission spectra of triarylpyrylium and triarylpyridinium dyes (**9-11a** and **9-11b**) in PBS.

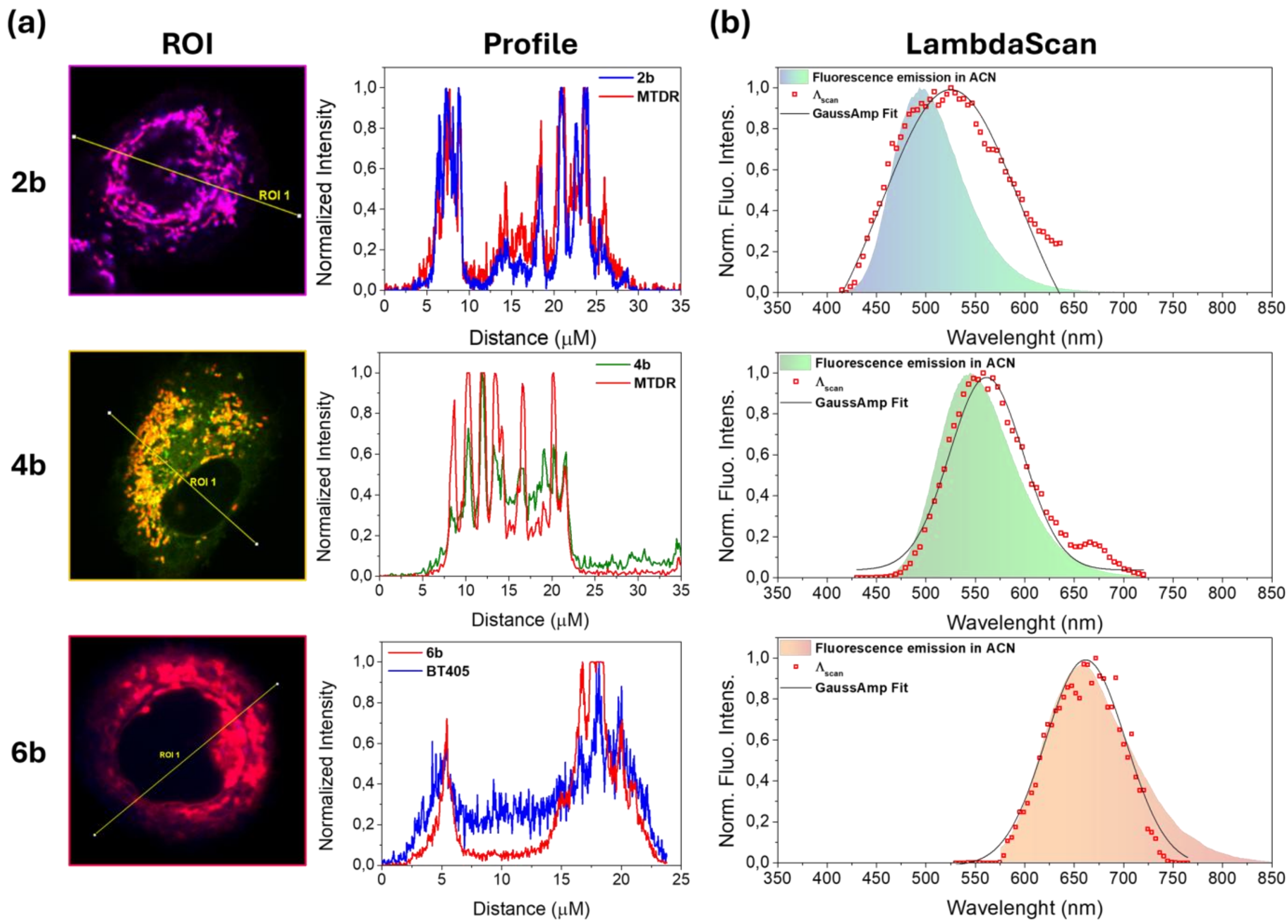

Figure S36. CLSM, line-profile analysis, and λ-Scan of compounds 2a, 4a, and 6b in A549 cells. (a) Representative images showing the ROIs (left) and the corresponding fluorescence line profiles (right) extracted along the indicated lines (normalized intensity vs distance). (b) λ-Scan emission: the area-filled curve shows the emission spectrum in ACN, red markers are the λ-Scan data, and the black line is the Gaussian fit. Scale bar: Scale bar: 20µm.

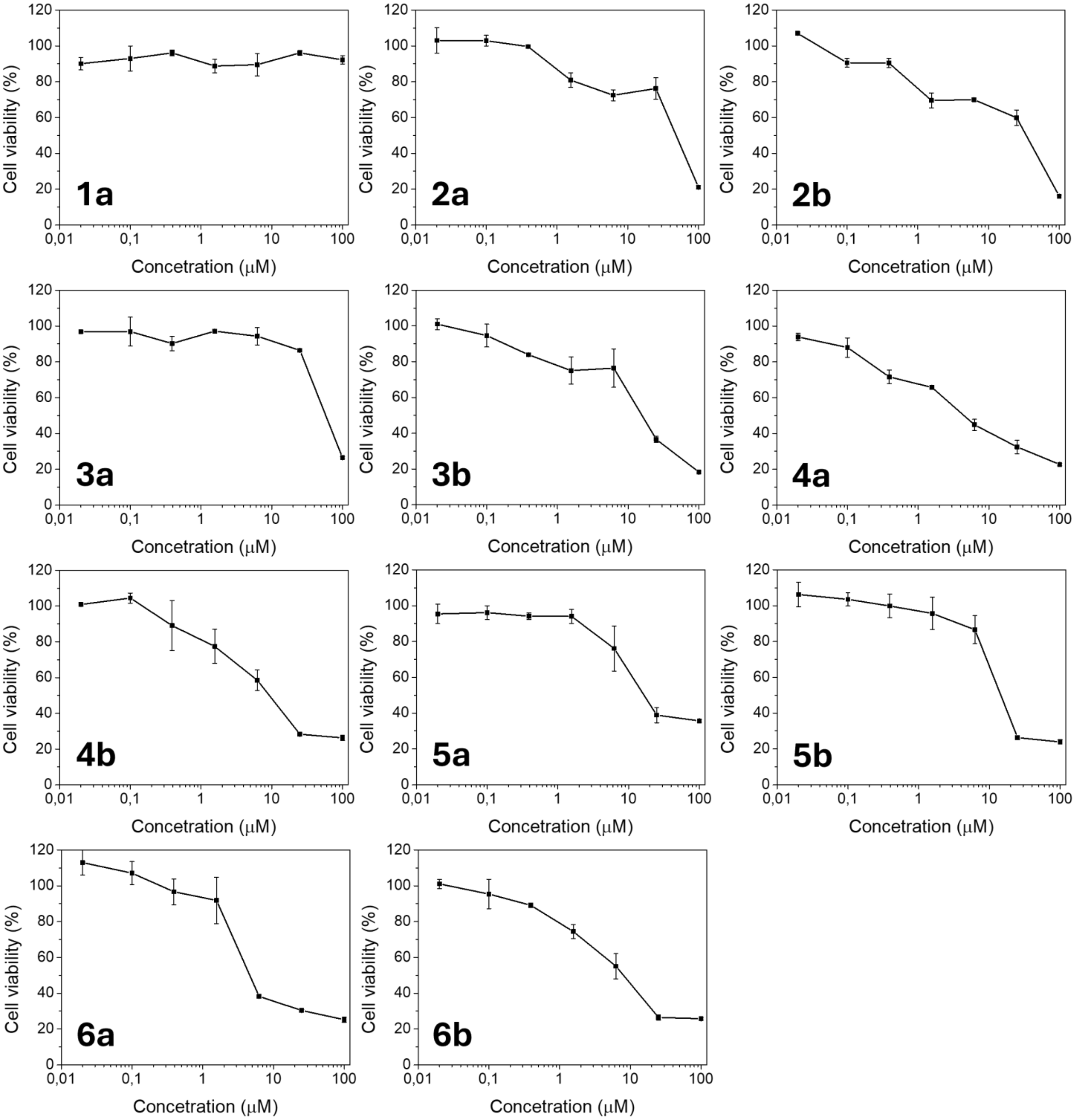

Figure S37. MTT cell viability assay performed in A549 cells after 24 h exposure to compounds of group **A** (**1a–6b**) at different concentrations. The X-axis represents the compound concentration in µM, while the Y-axis shows cell viability expressed as percentage relative to untreated control cells. Data are presented as mean ± standard deviation.

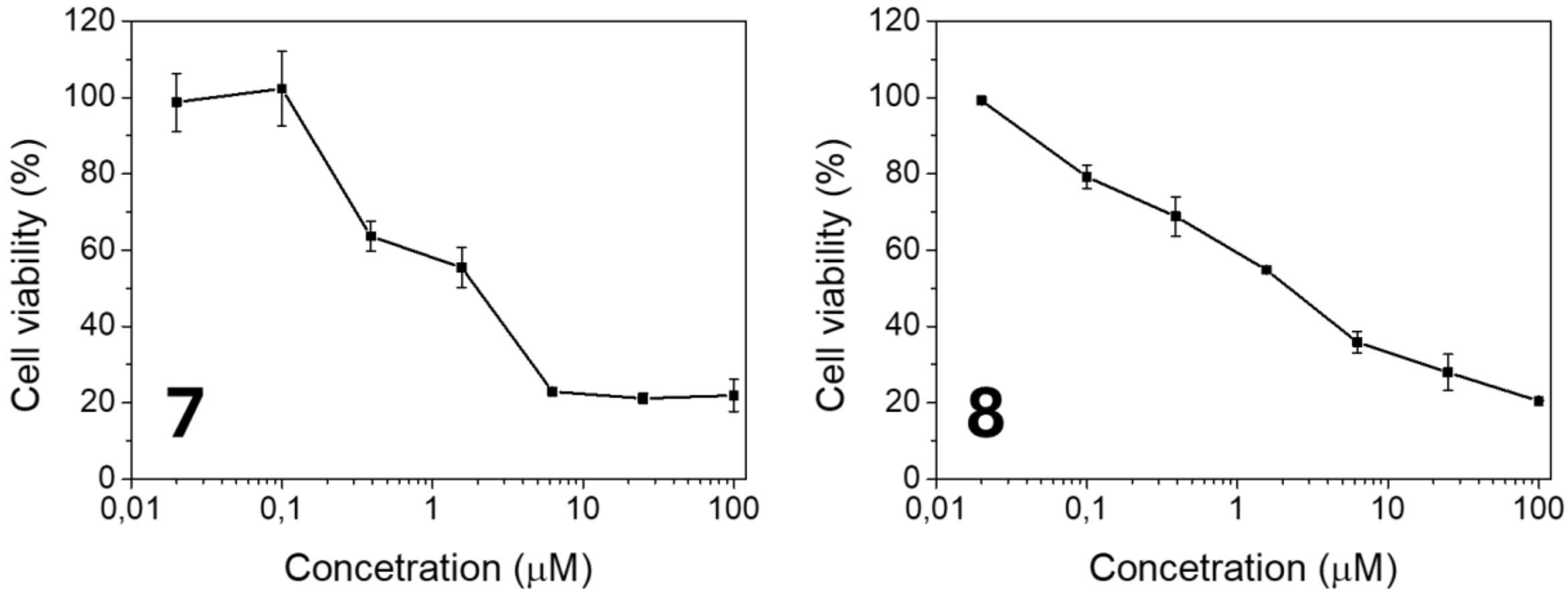

Figure S38. MTT cell viability assay performed in A549 cells after 24 h exposure to compounds of group **B** (**7** and **8**) at different concentrations. The X-axis represents the compound concentration in μM, while the Y-axis shows cell viability expressed as percentage relative to untreated control cells. Data are presented as mean ± standard deviation.

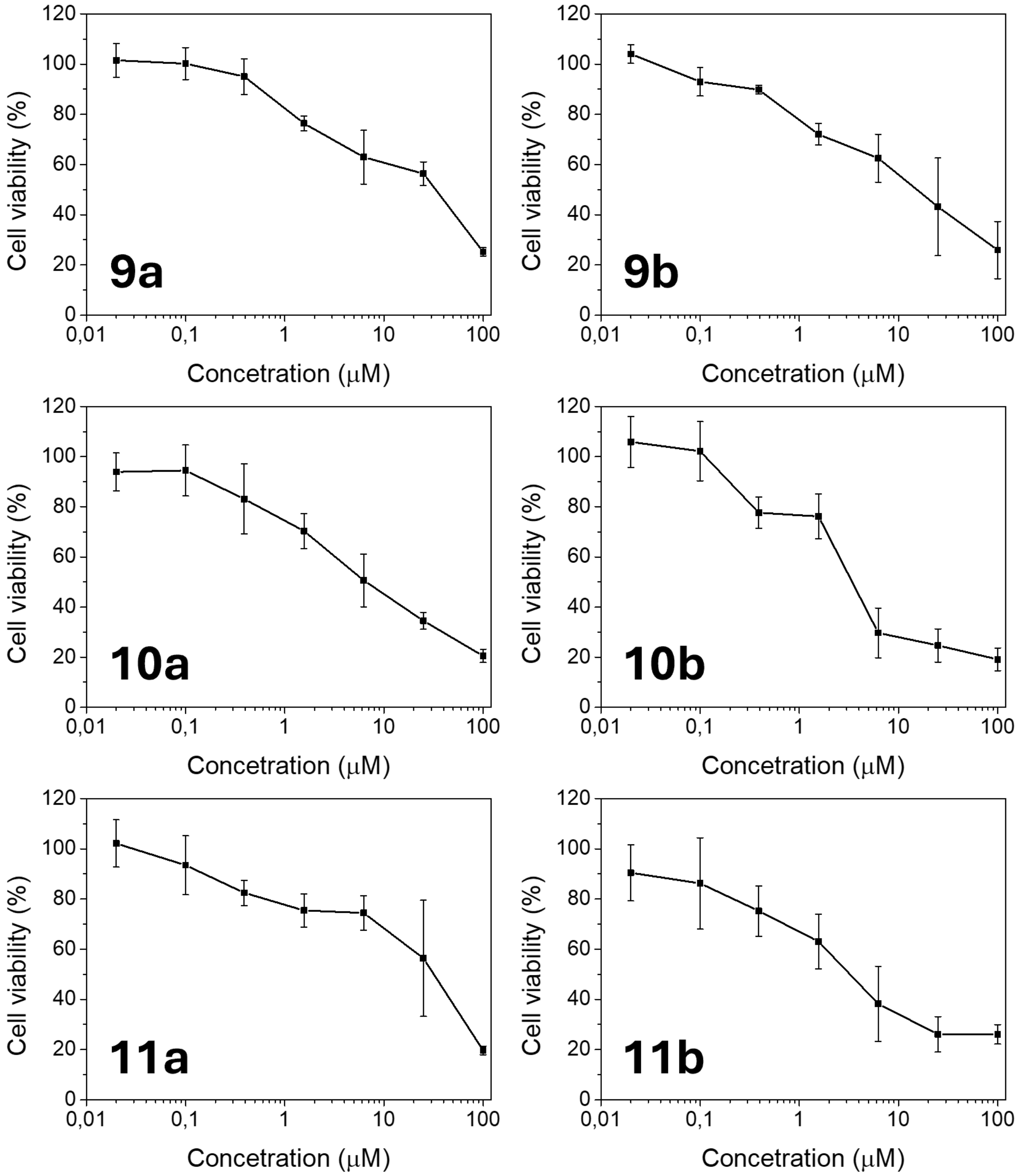

Figure S39. MTT cell viability assay performed in A549 cells after 24 h exposure to compounds of group **C** (**9a-11b**) at different concentrations. The X-axis represents the compound concentration in μM, while the Y-axis shows cell viability expressed as percentage relative to untreated control cells. Data are presented as mean ± standard deviation.

Supplemental references

(1) Neto, J. C.; Larek, A.; Miravet, J. F.; Yamaji, M.; Galindo, F. Organic, Transparent, and Flexible Films Exhibiting White-Light Emission via Polymer-Network Engineering: A Non-Dye-Centric Strategy. *Advanced Optical Materials* **2025**, *13* (27), e01380. https://doi.org/10.1002/adom.202501380.

(2) Eaton, D. F. Reference Materials for Fluorescence Measurement. *Pure and Applied Chemistry* **1988**, *60* (7), 1107–1114. https://doi.org/10.1351/pac198860071107.

(3) Seybold, P. G.; Gouterman, M.; Callis, J. Calorimetric, Photometric and Lifetime Determinations of Fluorescence Yields of Fluorescein Dyes. *Photochem Photobiol* **1969**, *9* (3), 229–242. https://doi.org/10.1111/j.1751-1097.1969.tb07287.x.

(4) Sens, R.; Drexhage, K. H. Fluorescence Quantum Yield of Oxazine and Carbazine Laser Dyes. *Journal of Luminescence* **1981**, *24–25*, 709–712. https://doi.org/10.1016/0022-2313(81)90075-2.

(5) Neto, J. C.; Lucantoni, F.; González, L. V.; Falomir, E.; Miravet, J. F.; Galindo, F. Introducing TAPY as a Versatile Alternative to TPP for Selective Mitochondrial Targeting in Cancer Cells. *Bioconjugate Chem.* **2025**, *36* (4), 697–706. https://doi.org/10.1021/acs.bioconjchem.4c00554.

(6) Molecular Probes. *Probes for Yeast Mitochondria*; Protocol MP 07530; ThermoFischer Scientific, 2001. https://assets.thermofisher.com/TFS-Assets/LSG/manuals/mp07530.pdf (accessed 2025-05-07).

(7) Ludovico, P.; Côrte-Real, M.; Sansonetty, F. Assessment of Mitochondrial Membrane Potential in Yeast Cell Populations by Flow Cytometry. *Microbiology* **2001**, *147* (12), 3335–3343. https://doi.org/10.1099/00221287-147-12-3335.

(8) Atkin, A. L. Preparation of Yeast Cells for Confocal Microscopy. In *Confocal Microscopy*; Humana Press: New Jersey, 1998; Vol. 122, pp 131–140. https://doi.org/10.1385/1-59259-722-X:131.

(9) Paddock, S. W. *Confocal Microscopy*; Humana Press: New Jersey, 1998; Vol. 122. https://doi.org/10.1385/159259722X.

(10) Daina, A.; Michielin, O.; Zoete, V. SwissADME: A Free Web Tool to Evaluate Pharmacokinetics, Drug-Likeness and Medicinal Chemistry Friendliness of Small Molecules. *Sci Rep* **2017**, *7* (1), 42717. https://doi.org/10.1038/srep42717; https://www.swissadme.ch/

(11) Wolfram, J.; Nizzero, S.; Liu, H.; Li, F.; Zhang, G.; Li, Z.; Shen, H.; Blanco, E.; Ferrari, M. A Chloroquine-Induced Macrophage-Preconditioning Strategy for Improved Nanodelivery. *Sci Rep* **2017**, *7* (1), 13738. https://doi.org/10.1038/s41598-017-14221-2.
